# Spatial proteogenomics reveals distinct and evolutionarily conserved hepatic macrophage niches

**DOI:** 10.1016/j.cell.2021.12.018

**Published:** 2022-01-20

**Authors:** Martin Guilliams, Johnny Bonnardel, Birthe Haest, Bart Vanderborght, Camille Wagner, Anneleen Remmerie, Anna Bujko, Liesbet Martens, Tinne Thoné, Robin Browaeys, Federico F. De Ponti, Bavo Vanneste, Christian Zwicker, Freya R. Svedberg, Tineke Vanhalewyn, Amanda Gonçalves, Saskia Lippens, Bert Devriendt, Eric Cox, Giuliano Ferrero, Valerie Wittamer, Andy Willaert, Suzanne J.F. Kaptein, Johan Neyts, Kai Dallmeier, Peter Geldhof, Stijn Casaert, Bart Deplancke, Peter ten Dijke, Anne Hoorens, Aude Vanlander, Frederik Berrevoet, Yves Van Nieuwenhove, Yvan Saeys, Wouter Saelens, Hans Van Vlierberghe, Lindsey Devisscher, Charlotte L. Scott

**Affiliations:** 1 Laboratory of Myeloid Cell Biology in Tissue Homeostasis and Regeneration, VIB-UGent Center for Inflammation Research, Technologiepark-Zwijnaarde 71, Ghent 9052, Belgium; 2 Department of Biomedical Molecular Biology, Faculty of Science, Ghent University, Belgium; 3 Hepatology Research Unit, Department Internal Medicine and Pediatrics, Liver Research Center, Ghent University, Belgium; 4 Gut-Liver Immunopharmacology Unit, Department of Basic and Applied Medical Sciences, Liver Research Center, Ghent University, Belgium; 5 Laboratory of Myeloid Cell Biology in Tissue Damage and Inflammation, VIB-UGent Center for Inflammation Research, Technologiepark-Zwijnaarde 71, Ghent 9052, Belgium; 6 Data Mining and Modelling for Biomedicine, VIB-UGent Center for Inflammation Research, Technologiepark-Zwijnaarde 71, Ghent 9052, Belgium; 7 Department of Applied Mathematics, Computer Science and Statistics, Faculty of Science, Ghent University, Ghent, Belgium; 8 VIB BioImaging Core, VIB-UGent Center for Inflammation Research, Technologiepark-Zwijnaarde 71, Ghent 9052, Belgium; 9 Laboratory of Immunology, Department of Translational Physiology, Infectiology and Public Health, Faculty of Veterinary Medicine, Ghent University, Belgium; 10 Institut de Recherche Interdisciplinaire en Biologie Humaine et Moléculaire (IRIBHM), Université Libre de Bruxelles (ULB), Brussels, Belgium; 11 ULB Institute of Neuroscience (UNI), Université Libre de Bruxelles (ULB), Brussels, Belgium; 12 WELBIO, Université Libre de Bruxelles (ULB), Brussels, Belgium; 13 Center for Medical Genetics Ghent, Department of Biomolecular Medicine, Ghent University, Ghent, Belgium; 14 KU Leuven Department of Microbiology, Immunology and Transplantation, Rega Institute, Laboratory of Virology and Chemotherapy, Molecular Vaccinology and Vaccine Discovery, Leuven, Belgium; 15 Laboratory of Parasitology, Department of Translational Physiology, Infectiology and Public Health, Faculty of Veterinary Medicine, Ghent University, Ghent, Belgium; 16 Laboratory of Systems Biology and Genetics, Institute of Bioengineering, School of Life Sciences, École Polytechnique Fédérale de Lausanne (EPFL), Lausanne, Switzerland; 17 Swiss Institute of Bioinformatics (SIB), Lausanne, Switzerland; 18 Oncode Institute, Department of Cell and Chemical Biology, Leiden Medical Center, Leiden, Netherlands; 19 Department of Pathology, Ghent University Hospital, Ghent 9000, Belgium; 20 Department of General and Hepatopancreatobiliary Surgery and Liver Transplantation, Ghent University Hospital, Ghent 9000, Belgium; 21 Department of Human Structure and Repair, Ghent University Hospital, Ghent 9000, Belgium; 22 Department of Gastroenterology and Hepatology, Ghent University Hospital, Ghent 9000, Belgium

**Keywords:** liver, atlas, spatial transcriptomics, CITE-seq, proteogenomic, across species, multi-omic, lipid-associated macrophage, Kupffer cell, NAFLD

## Abstract

The liver is the largest solid organ in the body, yet it remains incompletely characterized. Here we present a spatial proteogenomic atlas of the healthy and obese human and murine liver combining single-cell CITE-seq, single-nuclei sequencing, spatial transcriptomics, and spatial proteomics. By integrating these multi-omic datasets, we provide validated strategies to reliably discriminate and localize all hepatic cells, including a population of lipid-associated macrophages (LAMs) at the bile ducts. We then align this atlas across seven species, revealing the conserved program of bona fide Kupffer cells and LAMs. We also uncover the respective spatially resolved cellular niches of these macrophages and the microenvironmental circuits driving their unique transcriptomic identities. We demonstrate that LAMs are induced by local lipid exposure, leading to their induction in steatotic regions of the murine and human liver, while Kupffer cell development crucially depends on their cross-talk with hepatic stellate cells via the evolutionarily conserved ALK1-BMP9/10 axis.

## Highlights

• Spatial proteogenomic single-cell atlas of healthy and obese murine and human liver
• Validated flow cytometry and microscopy panels for all hepatic cells
• LAMs are differentially located in the lean and obese liver
• Evolutionary conserved BMP9/10-ALK1 axis is essential for KC development

## Graphical abstract

**Figure:**
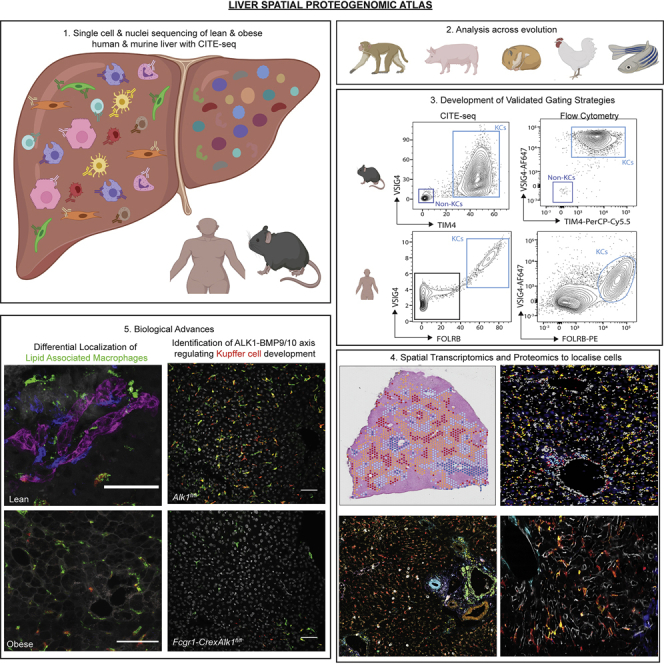

## Teaser

By combining single-cell and -nucleus sequencing with spatial mapping of RNA and proteins, this vast spatial proteogenomic atlas of healthy and obese human and mouse livers presents methods to identify and localize all hepatic cells and provides insights into hepatic myeloid cells, including identification of reliable surface markers for isolation and localization of hepatic macrophages, characterization of lipid-associated macrophages in both healthy and steatotic livers, determination of a key regulatory axis of Kupffer cell development, and identification of a conserved core gene expression signature of Kupffer cells across 7 species, including chickens and zebrafish.

## Introduction

The immense advances in single-cell transcriptomics have enabled a better understanding of the cellular composition of different organs across species. However, we still lack information regarding how these cells are organized in their distinct microenvironmental niches. Moreover, the specific cell-cell interactions determining the identity of individual cells within tissues remain to be defined (Guilliams and Scott, 2017; Lindeboom et al., 2021). While the spatial organization of hepatocytes within the liver is understood (Halpern et al., 2017), that of non-parenchymal liver cells remains unclear. This is the case for the mouse liver, but even more so for the human liver, where the identity and the precise localization of most hepatic cells is unknown. Moreover, the link between the transcriptome and the proteome has not been studied, resulting in a lack of reliable surface markers to identify these cells by flow cytometry and confocal microscopy. Here, we used proteogenomic techniques including cellular indexing of transcriptomes and epitomes by sequencing (CITE-seq) and spatial approaches to identify all cells and their specific locations within the healthy and obese livers of mice and humans. By doing so, we have developed strategies for the identification and further study of hepatic cells. Demonstrating the usefulness of this approach, we identify the evolutionary conserved and spatially restricted signals driving the distinct hepatic macrophage phenotypes.

## Results

### A practical proteogenomic atlas of the murine liver

**Figure S1:**
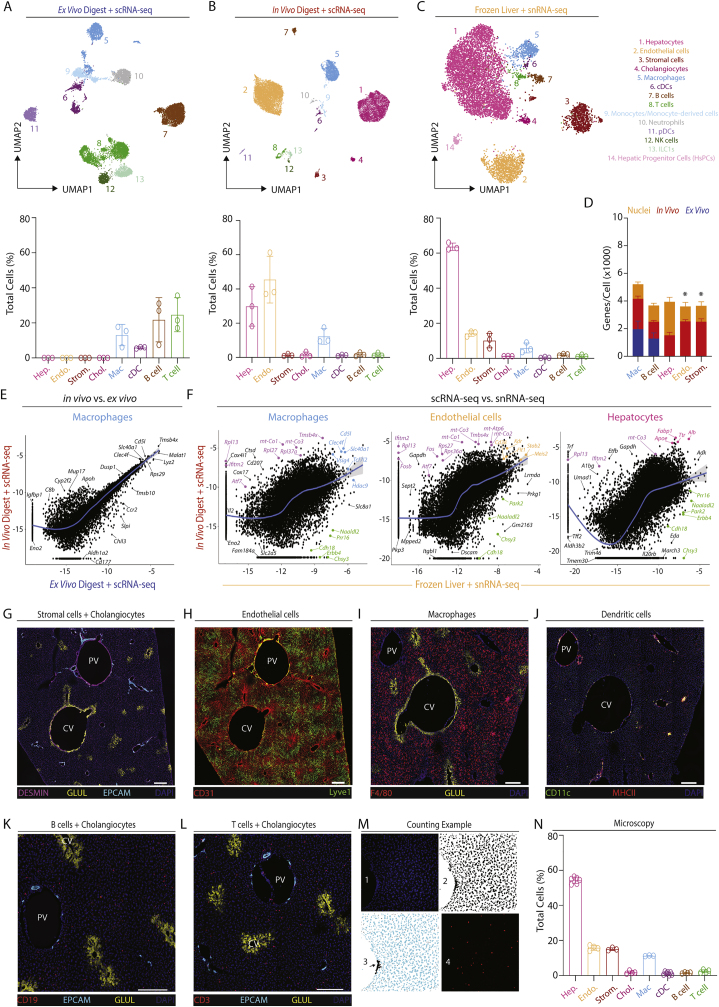
Cell types identified in transcriptomic studies depend upon cell/nuclei isolation technique used, related to Figure 1 Cells were isolated from livers of healthy C57B/l6 mice by either *ex vivo* or *in vivo* enzymatic digestion. Alternatively, livers were snap frozen and nuclei subsequently isolated following tissue homogenization by a sucrose gradient (3 mice per isolation method). Live cells/intact nuclei were identified and purified using flow cytometry. For the cells, either live CD45^+^, live CD45^−^ or live hepatocytes were sorted. 1 *ex vivo* digested sample and 1 *in vivo* digested sample were also stained with a panel of 107 (*ex vivo* cells) or 161 (*in vivo* cells) oligo-conjugated antibodies for CITE-seq analysis. FACS-purified cells/nuclei were loaded onto the 10× chromium platform and scRNA-seq, CITE-seq, or snRNA-seq performed. Following clean up and QC, cells from the same mice were pooled together in the same ratios (CD45^+^:CD45^−^:Heps) as found in the tissue as a whole before sorting, different mice were then pooled together and the data were analyzed using scVI. (A–C) UMAPs showing annotations of cell types and proportions of each cell type as a % of total cells in the UMAP isolated using (A) *ex vivo* digestion; 13,144 cells, (B) *in vivo* digestion; 24,014 cells and (C) nuclei; 8,583 nuclei. (D) Average number of genes/cell in the annotated mac, B cell, hepatocyte, endothelial, and stromal cell populations following each isolation method. ^∗^p < 0.05 one-way ANOVA with Bonferroni post-test per cell type. (E) Correlation plot showing genes captured within the mac population when the liver is digested with the *in vitro* versus the *in vivo* digestion protocol. (F) Correlation plots showing genes captured within the mac, endothelial cell, and hepatocyte populations when cells are isolated using the *in vivo* digestion protocol or nuclei are isolated. (G–L) Confocal microscopy images to determine true abundance of (G) stromal cells and cholangiocytes (H) endothelial cells, (I) macs, (J) dendritic cells, (K) B cells, and (L) T cells *in vivo*. Scale bars, 200 μm. (M) The percentage of each population was calculated based on the percentage of a given population divided by the total number of nuclei. A threshold was applied to the DAPI channel (picture 1) in ImageJ (picture 2) and nuclei were automatically counted based on the ImageJ “analyze particles” plugin (size ). Due to the density of some liver zones, some nuclei were not automatically counted (arrow, picture 3). Those were then manually counted and added to the total number of nuclei. For the populations of interest, cells were counted manually based on specific markers (for example, CD3 for T cells, picture 4). Counting was performed blinded prior to analysis of the sequencing results. (N) Proportion of indicated cell types as a % of total cells identified in confocal microscopy images. Data are from 3–7 images per cell type taken from 2–4 mice.

**Figure 1:**
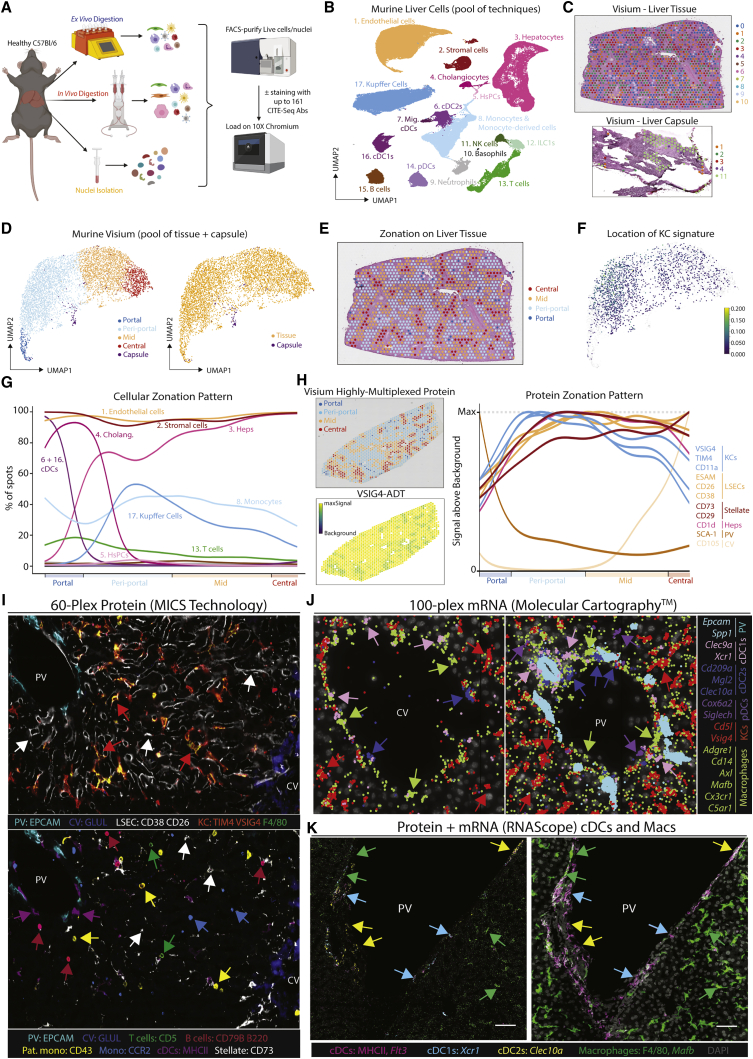
A proteogenomic atlas of the healthy murine liver (A) Hepatic cells were isolated from healthy C57B/l6 mice by *ex vivo* (5 mice, 15 samples) or *in vivo* (5 mice, 19 samples) enzymatic digestion. Alternatively, nuclei were isolated by tissue homogenization (4 mice, 12 samples). Live cells/intact nuclei were FACS-purified. For cells, total live, live CD45^+^, live CD45^−^, live hepatocytes, or myeloid cells (live CD45^+^, CD3^−^, CD19^−^, B220^−^, NK1.1^−^) were sorted. 18 samples (7 *ex vivo*, 11 *in vivo*) were also stained with a panel of 107–161 barcode-labeled antibodies for CITE-seq analysis. All datasets were pooled together and after QC 185,894 cells/nuclei were clustered using TotalVI. (B) UMAP of sc/snRNA-seq data. (C) Tissue and capsule images from Visium analysis with clusters overlaid. (D) UMAP of zonation of Visium spots (left) and origin of the cells (right). (E) Zonation pattern mapped onto tissue slice. (F and G) Indicated cell signatures from sc/snRNA-seq mapped onto the Visium zonation data. (H) mRNA zonation pattern in Visium highly multiplexed protein analysis and VSIG4-ADT expression pattern (left) and zonated expression patterns of indicated antibodies (right). (I) MICS analysis of indicated proteins. (J) Molecular Cartography of indicated genes and cell types. (K) mRNA (*Xcr1, Flt3l, Mafb*, and *Clec10a*) and protein (MHCII and F4/80) expression in the same tissue slice. Scale bars, 50 μm. PV, portal vein; CV, central vein. Arrows indicate specific cell types, colors correspond to cell type/markers. Images are representative of 2–4 mice. See also Figure S2 and Tables S1, S2, S3, and S5.

**Figure S2:**
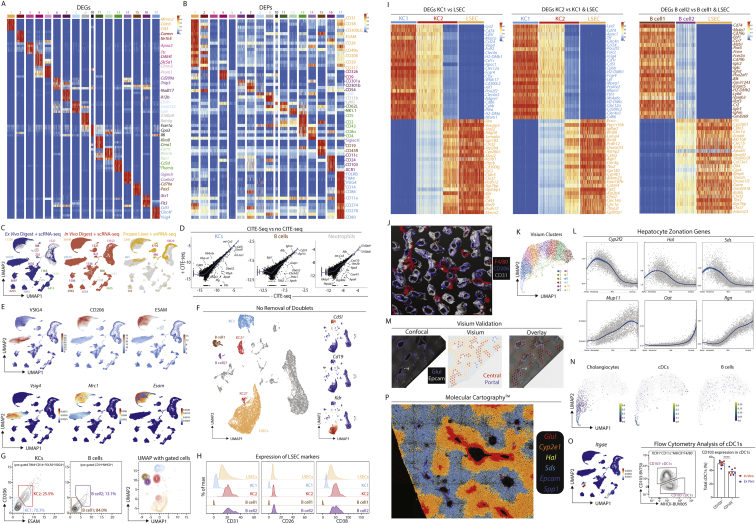
Combination of CITE-seq, scRNA-seq, snRNA-seq, and spatial analyses enables identification of all hepatic cell types including bona fide cell doublets, related to Figure 1 (A and B) Top DEGs (A) and DEPs (B) for cell types from Figure 1B. (C) Distinct profiles of cells or nuclei within the UMAP depending on isolation protocols; 71,162 cells from *ex vivo* digestions, 96,066 cells from *in vivo* digestions, and 18,666 nuclei. Numbers on plots represent numbers of cells/nuclei per population. (D) Correlation plots showing genes captured within the KC, B cell and neutrophil populations with and without addition of CITE-seq antibodies. (E) Expression of VSIG4, CD206, and ESAM (protein, top) and *Vsig4*, *Mrc1*, and *Esam* (mRNA, bottom). (F) UMAP showing clusters of cells when only minimal QC for gene number and % mitochondrial genes is performed; 17,669 cells pooled from 3 samples. Expression of *Cd5l, Cd19*, and *Kdr* by the clusters facilitating identification of cell types per annotation. (G) CITE-seq data from (F) in Flow-Jo showing expression of CD206 and ESAM in total KCs (left) and total B cells (middle). Numbers represent % of entire KC or B cell population. Identified populations were then mapped back onto the original UMAP (right). (H) Expression of CD31, CD26, and CD38 by indicated populations. (I) Heatmaps showing expression of top DEGs between KC1s and LSECs (left), KC2s and KC1s + LSECs (middle) and B cell2s and B cell1s + LSECs (right). (J) 3D reconstruction of murine liver following perfusion with antigen fix to inflate endothelial cells and staining with antibodies against CD31, CD206, and F4/80. (K) UMAP showing clusters generated from Visium analysis of liver tissue (4 samples) and liver capsule (1 sample). (L) Top unbiased genes defining zonation trajectory from portal to central vein in Visium. (M) Expression of *Glul* and *Epcam* by confocal microscopy (left), annotation of portal, periportal, mid, and central regions on same tissue section (middle) and overlay of both datasets (right). (N) Identification of cholangiocyte (left) and cDC (right) signatures on zonated Visium spots. (P) Molecular Cartography showing expression of indicated zonated hepatocyte mRNAs in liver tissue. Data are representative of 2 mice. (O) Expression of *Itgae* (encoding CD103) in the UMAP of the total liver (left) and flow cytometric analysis of total cDC1s for CD103 and MHCII expression in the healthy murine liver (right).

**Figure 2:**
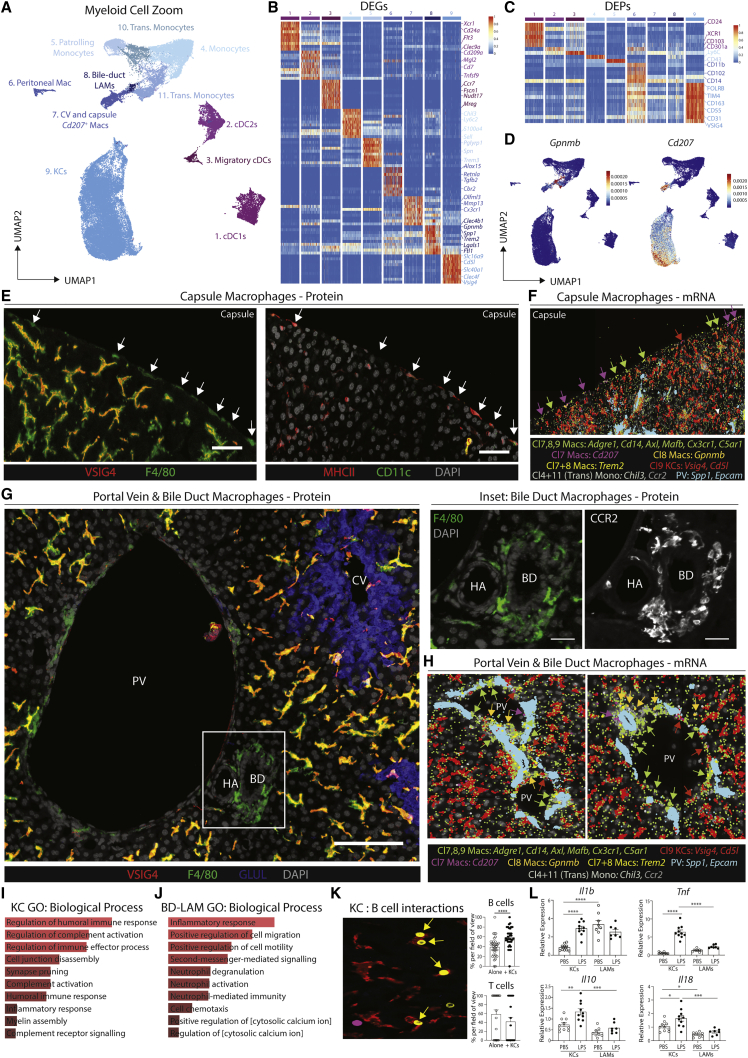
A population of macrophages reside around the bile duct in the healthy murine liver (A) UMAP of murine myeloid cells (71,261 cells/nuclei) isolated from Figure 1B and re-clustered with TotalVI. (B and C) Top DEGs (B) and DEPs (C) between cell types. (D) Expression of *Gpnmb* and *Cd207*. (E) Expression of VSIG4 and F4/80 (left) or MHCII, CD11c, and DAPI (right) by confocal microscopy. Capsule macs identified by white arrows. Scale bars, 50 μm. (F) Molecular Cartography of indicated genes at liver capsule. (G) Expression of VSIG4, F4/80, GLUL, and DAPI (left) or F4/80 or CCR2 (right, inset) by confocal microscopy. Scale bars, 100 μm. (H) Molecular Cartography of indicated genes at portal triad. PV, portal vein; CV, central vein; HA, hepatic artery; BD, bile duct. Arrows indicate specific cell types, where color corresponds to markers. Images are representative of 2–4 mice. (I and J) Top GO terms for KCs (I) and bile-duct LAMs (J). (K) Representative image showing expression of VSIG4 (red) CD19 (yellow) and CD3E (magenta) by MICS analysis (left) and % of B or T cells found with/without a KC per field of view (right). Data are pooled from multiple fields of view in 2 mice. ^∗∗∗^p < 0.001 Student’s t test. (L) Mice (29-week-old) were treated with 3.5 mg/kg LPS or PBS and 2 h later, livers were harvested without the capsule. KCs and LAMs were FACS-purified and expression of *Il1b, Tnf, IL10*, and *Il18* was examined by qPCR, compared with *b-actin*. ^∗^p < 0.05, ^∗∗^p < 0.01, ^∗∗∗^p < 0.001, ^∗∗∗∗^p < 0.0001, one-way ANOVA with Bonferroni post-test. See also Figure S3 and Table S2.

To generate a proteogenomic atlas of the liver, we first examined the optimal method for retrieving all hepatic cells. Using the murine liver, we compared single-cell RNA sequencing (scRNA-seq) using cells isolated via *ex vivo* or *in vivo* enzymatic digestion with single-nuclei RNA sequencing (snRNA-seq) (Figures S1A–S1C). We did not observe any differences in the number of genes/cell between the two digestion methods (Figures S1D and S1E), but snRNA-seq typically yielded a lower number of genes/cell (Figures S1D and S1F). This did not prevent distinct cell types from being identified in the snRNA-seq dataset as both scRNA-seq and snRNA-seq identified highly expressed genes in each population. However, expression was often higher in the scRNA-seq (Figure S1F). Additionally, we observed a signature of digestion-associated genes and snRNA-seq-associated genes across cell types (Figure S1F). While in terms of genes/cell snRNA-seq is inferior to scRNA-seq, this method best recapitulated the cell frequencies observed *in vivo* (Figures S1D–S1N). As each method has advantages and disadvantages, the optimal method to use depends on the biological question being addressed. Here, as we sought to generate a proteogenomic atlas of all hepatic cells, we thus opted to use a combination of all protocols. To investigate mRNA and protein expression at single-cell resolution, we used CITE-seq (Stoeckius et al., 2017), staining a selection of the scRNA-seq samples with 107–161 oligo-conjugated antibodies (Figure 1A). Data were pooled together for a single analysis where, with TotalVI (Gayoso et al., 2021), both the protein and mRNA profiles were considered for clustering (Figure 1B). Analysis of the differentially expressed genes and proteins (DEGs/DEPs; Figures S2A and S2B; Table S1) identified 17 cell types (Figure 1B), which were differentially represented with each isolation method (Figure S2C). Addition of antibodies in the CITE-seq analysis enabled surface markers for all cells to be identified, including VSIG4 and FOLR2 for Kupffer cells (KCs) (Figure S2B), without affecting the quality of the transcriptomic data (Figure 2D). This analysis identified one subset of KCs. However, 2 subpopulations termed KC1 and KC2 have recently been described (Blériot et al., 2021; Simone et al., 2021). As our CITE-seq analysis identified that the markers used to identify KC2s, namely CD206 and ESAM, are largely expressed by liver sinusoidal endothelial cells (LSECs) (Figure S2E), we next sought to determine if we had previously removed any potential KC2s in our initial QC steps as LSEC-KC doublets. To examine this, we generated a UMAP of samples containing CD206 and ESAM in the CITE-seq panel and performed a minimal QC filtering on number of genes and percentage of mitochondrial genes. In this UMAP, we found multiple subpopulations including 3 populations of KCs and 2 populations of B cells (Figure S2F). To determine if any of these populations could be KC2s, we harnessed the power of CITE-seq to recreate the gating strategy recently proposed to identify KC2s (Blériot et al., 2021). Converting the CITE-seq data into an FCS file and analyzing this in FlowJo identified 2 of the KC populations to be KC2s expressing CD206 and ESAM, and these cells were present in a similar ratio among KCs as reported by Blériot et al. (Figure S2G). However, employing the same gating strategy with the B cells also enabled a B cell2 population to be identified (Figure S2G). The KC2 and B cell2 populations also expressed other protein markers associated with LSECs including CD26, CD31, and CD38, suggesting that these may be doublets (Figure S2H). Consistent with this, we did not uncover any DEGs specifically expressed in the KC2s or B cell2s, rather these cells had an intermediate profile between either KC1s or B cell1s and LSECs (Figure S2I), as would be expected for doublets. Finally, we perfused the livers with antigen fix to inflate the LSECs to be able to distinguish more readily between KCs and LSECs and performed confocal microscopy. 3D reconstruction of these images indicates that CD206 expression is observed primarily in the LSECs with which the KCs are intertwined. These microscopy images also show that KCs express some CD206, however, consistent with our CITE-seq analysis, this was observed across the KC population rather than in a KC subset further suggesting the existence of only one-KC population (Figure S2J). With this in mind, we decided to continue to apply our initial strict QC controls eliminating these potential doublets from further analyses.

### Distinct spatial orientation of hepatic myeloid cell subsets

To locate the cells identified we performed spatial transcriptomics analysis using Visium. For this, we cut the liver in two distinct orientations to profile both the liver tissue and the capsule (Figures 1C and S2K). We ordered each Visium spot along a spatial trajectory, and annotated portal, periportal, mid, and central zones based on known hepatocyte zonation markers (Halpern et al., 2017; Figures 1D, 1E, and S2L) and confirmed this annotation using confocal microscopy (Figure S2M). By using the reference sc/snRNA-seq data, we then deconvolved each spot into its constituent cell types and investigated how cell abundance changed with zonation (Figures 1F, 1G, and S2N). Validating this approach, cholangiocytes mapped specifically to the portal zones (Aizarani et al., 2019), while KCs were preferentially located in periportal and mid zones (Bonnardel et al., 2019; Gola et al., 2021). While KC location is zonated, we did not identify a strong zonation pattern in the gene expression profiles of KCs (data not shown). We further identified B cells, T cells, endothelial cells (ECs), and stromal cells (SCs) across all zones, while conventional dendritic cells (cDCs) were found at the portal vein (PV), with a minor presence at the central vein (CV) (Figures 1F, 1G, and S2N).

To validate these locations at single-cell resolution, we next sought to identify the best cell-specific surface markers that would also work by confocal microscopy. As the fixation step utilized for confocal microscopy often affects the integrity of protein epitopes, it is not possible to predict which antibodies will work spatially on fixed tissue slices. Therefore, to simultaneously screen multiple antibodies to identify those working by microscopy, we performed a second Visium analysis which we complemented with 100 oligo-conjugated antibodies, chosen based on the CITE-seq results (Figure 1H). The antibodies identified to work spatially were then validated at single-cell resolution, using MACSima™ Imaging Cyclic Staining (MICS) technology and a 60-plex antibody panel (Figure 1I). Unfortunately, we could not identify useful surface markers for all populations. For example, we did not identify enough discriminatory surface markers that worked by confocal microscopy to distinguish the cDC subsets. Notably, while CD103 has previously been proposed to distinguish hepatic cDC1s from cDC2s (Eckert et al., 2015), our data demonstrate that this is only expressed by a fraction of these cells (Figure S2O). To confirm the locations of the cDC subsets, we thus turned to Molecular Cartography™ (Resolve BioSciences) that allows for 100-plex spatial mRNA analysis. Genes were selected based on the DEGs from the sc/snRNA-seq data that were also spatially resolved according to Visium. We also identified the portal-central trajectory in this dataset using cholangiocytes genes (*Epcam* and *Spp1*) and known zonated hepatocyte genes (*Glul, Cyp2e1, Hal,* and *Sds*; Figure S2P). Using expression of *Xcr1, Clec9a* (cDC1s) and *Cd209a, Mgl2*, and *Clec10a* (cDC2s), we confirmed that both cDC1s and cDC2s were localized primarily at the PV (Figure 1J). As cDC2s shared a number of genes with monocyte-derived cells (Figure S2A), we also examined the expression of general monocyte/mac (*Cd14*, *Adgre1*, *Axl*, *Mafb*, *Cx3cr1*, and *C5ar1*) and KC-specific genes (*Cd5l* and *Vsig4*) to further validate their identification as bona fide cDC2s (Figure 1J). The punctate nature of mRNA expression in these analyses combined with the dendritic shape of myeloid cells renders it difficult to convincingly determine cell boundaries and to conclude these cDC2s and macs were distinct cells. To validate this, we therefore developed a protocol that combines mRNA detection (RNAScope) with surface protein detection. Examining expression of cDC- or mac-specific mRNAs combined with protein surface markers confirmed the presence of PV cDC1, cDC2s, and non-KC macs (Figure 1K). Taken together, by combining multiple spatial transcriptomic and proteomic approaches, we located all the cells within the murine liver and identified additional heterogeneity within the myeloid cells, not revealed when examining the sc/sn-RNA-seq dataset in isolation. This highlights the power of combining single-cell and spatial proteogenomic techniques to investigate cellular heterogeneity.

### Refined analysis of myeloid cells identifies three subsets of hepatic macrophages

**Figure S3:**
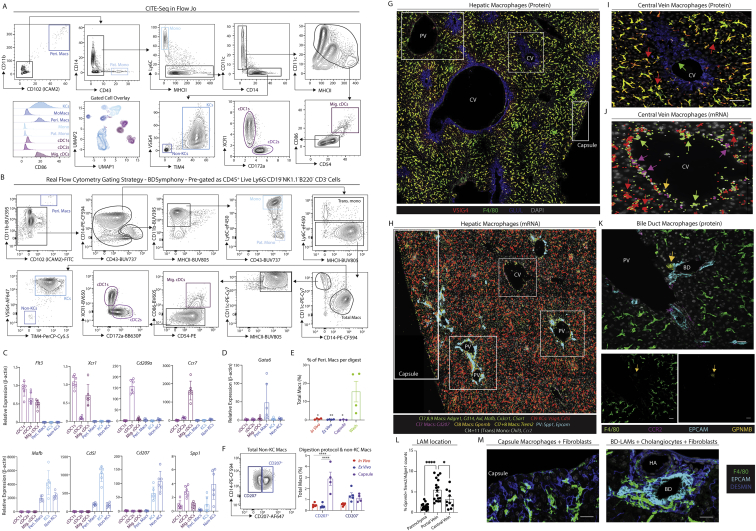
Validated flow cytometry gating strategy for murine myeloid cells, related to Figure 2 (A) CITE-seq data from the murine myeloid cells in Figure 2A were exported as an FCS file and an *in silico* gating strategy identified in FlowJo software. (B) Application of the *in silico* gating strategy with a 21-color flow cytometry panel. Myeloid cells were pre-gated as live CD45^+^ lineage cells (Ly6G^−^CD19^−^NK1.1^−^B220^−^CD3^−^). Data are representative of 3 experiments with 3–6 mice per experiment. (C) cDC1s, cDC2s, migratory cDCs (Mig. cDCs), peritoneal macs (Peri. Macs), KCs, and non-KC macs (non-KCs) were FACS-purified using gating strategy in (B), mRNA was isolated and qPCR performed to examine expression of indicated genes defining each population to validate their identity. Data are representative of 2 experiments with n = 3–6. (D) Putative peritoneal macs were FACS-purified using gating strategy in (B) and expression of *Gata6* was examined by qPCR compared with other hepatic myeloid populations. Data are from a single experiment with n = 6. (E) Peritoneal macs as a % of total macs recovered from the liver using different digestion techniques (*in vivo*, *ex vivo*, or capsule) or in supernatants in which livers were washed following removal from the mouse but prior to digestion (wash). Data are from a single experiment with n = 4. ^∗^p < 0.05, ^∗∗^p < 0.01 one-way ANOVA with Bonferroni post-test compared with wash data. (F) Expression of CD14 and CD207 within the non-KC mac population from (B) (left) and % of CD207^+^ and CD207^−^ populations among total macs in livers digested using the *ex vivo* or *in vivo* protocols or in dissected and digested liver capsule (right). Data are representative of two experiments with n = 4–5 mice per experiment. ^∗∗∗∗^p < 0.0001 mixed effects analysis with Tukey’s multiple comparison test. (G) Expression of VSIG4, F4/80, GLUL, and DAPI by confocal microscopy. Insets represent zones featured in Figures 2E, 2G, and S3I. (H) Molecular Cartography of indicated genes and cell types. Insets represent zones featured in Figures 2F, 2H, and S3J. (I) Expression of VSIG4, F4/80, GLUL, and DAPI by confocal microscopy at the central vein. Scale bars, 50 μm. (J) Molecular Cartography of indicated genes and cell types at central vein. (K) Expression of F4/80, EPCAM, CCR2, GPNMB, and DAPI by confocal microscopy at a portal vein (top) or F4/80 or GPNMB alone (bottom). Scale bars, 25 μm. (L) Quantification of % of *Gpnmb* & *Trem2* counts over *Adgre1* counts in indicated regions of tissue as assessed using Molecular Cartography data. Each dot represents an individual region. ^∗^p < 0.05, ^∗∗∗∗^p > 0.0001 one-way ANOVA with Bonferroni post-test. (M) Expression of DESMIN and F4/80 at the liver capsule and underlying parenchyma (left) or EPCAM, DESMIN and F4/80 at the bile duct by confocal microscopy. PV, portal vein; CV, central vein; HA, hepatic artery; BD, bile duct. Arrows indicate specific cell types, where color corresponds to cell type/markers. All images are representative of 2–6 mice.

To better understand the non-KC macs, we zoomed in on myeloid cells (cDCs, KCs, monocytes, and monocyte-derived cells) in our sc/snRNA-seq analysis defining 11 populations (Figures 2A–2C; Table S2). This included KCs, 3 populations of non-KC macs and cells that had a profile intermediate between monocytes and patrolling monocytes or macs, termed transitioning monocytes. Closer inspection of the non-KC macs identified cluster6 as peritoneal macs (Figure 2B). The DEGs between the remaining populations suggested that cluster7 likely resembles capsule macs (Sierro et al., 2017), expressing *Cd207 and Cx3cr1* while cluster8 resembles *Gpnmb*^*+*^*Spp1*^+^ lipid-associated macrophages (LAMs) we recently described in the fatty liver (Remmerie et al., 2020; Figures 2A–2D). Conversion of the CITE-seq data into an FCS file allowed an *in silico* gating strategy to be defined (Figure S3A). Validating this, we utilized the strategy to FACS-purify the populations and assess gene expression (Figures S3B–S3D). Fitting with a recent report (Jin et al., 2021), washing the liver prior to digestion enriched the peritoneal macs in the wash fraction, demonstrating these were contaminants on the liver surface rather than being present in the liver tissue itself (Figure S3E). While the CITE-seq markers did not discriminate between cluster7 and cluster8, adding CD207 to the panel enabled the non-KCs to be divided into CD207^+^ and CD207^−^ macs (Figure S3F). Fitting with their designation as capsule macs, the relative abundance of CD207^+^ macs was increased if we dissected and digested the capsule (Figure S3F). However, although Molecular Cartography confirmed the presence of *Cd207*^*+*^ macs in the capsule, it also revealed *Cd207*^*+*^ macs at the CV, which were rarely found at the PV (Figures 2E–2H and S3G–S3J). Thus, cluster7 consists of both capsule and CV CD207^+^ macs. This finding further demonstrates the need for spatial approaches to confirm cell identities. Molecular Cartography also identified macs at the PVs and CVs expressing *Ccr2* and *Chil3* (Figures 2G, 2H, S3H, and S3J), resembling transitioning monocytes (cluster11). Finally, a population of *Gpnmb*-expressing macs were found to be enriched around the bile ducts (Figures 2G, 2H, S3H, S3K, and S3L). As *Gpnmb* expression is cluster8 specific (Figure 2B), and these cells resemble LAMs (Remmerie et al., 2020), we termed these cells bile-duct LAMs.

**Figure S4:**
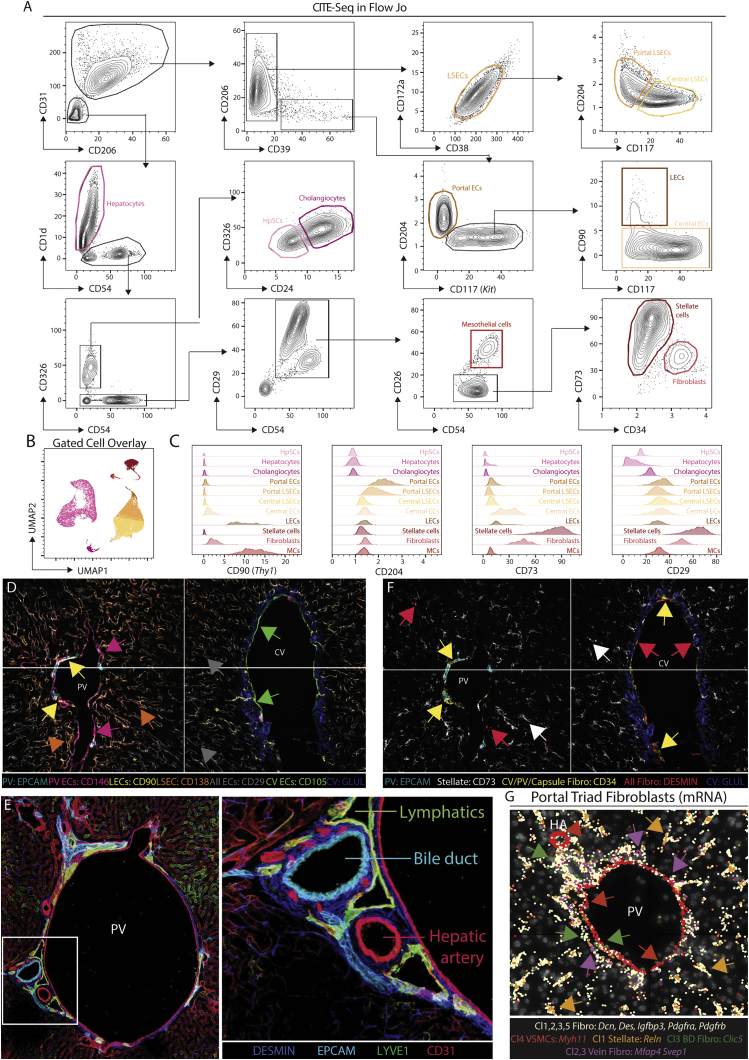
Protein markers of murine CD45^−^ cell subsets, related to Figure 3 (A) CITE-seq data from the murine CD45^−^ cells in Figure 3A were exported as an FCS file and an *in silico* gating strategy identified in FlowJo. (B) Gated cell overlay of populations identified using strategy in (A). (C) Expression of CD90, CD204, CD73, and CD29 markers by indicated cell types. (D) Expression of indicated protein markers in 60-plex MICS analysis in endothelial cells. (E) Expression of DESMIN, EPCAM, LYVE1, and CD31 at a portal triad (left) with inset (right). (F) Expression of indicated protein markers in 60-plex MICS analysis in stromal cells. (G) Molecular Cartography of indicated genes and cell types at portal vein. PV, portal vein; CV, central vein. Arrows indicate specific cell types, where color corresponds to cell type/markers. All images are representative of 2–6 mice.

### KCs and LAMs are functionally distinct in the homeostatic murine liver

We next sought to investigate the differences between KCs and LAMs. Analysis of GO terms associated with biological processes for these cells suggested that KCs may play a role in regulating humoral responses, while LAMs were more broadly associated with immune responses (Figures 2I and 2J). Consistent with this, in the 100-plex protein microscopy data we noted that a significant proportion of the B cells present were interacting with KCs, which was not observed with T cells (Figure 2K). This suggests cross-talk between these two populations, potentially linked to the high expression of the B cell chemokine *Cxcl13* by murine KCs (Table S2). To assess the inflammatory nature of LAMs compared with KCs, we FACS-purified the cells and performed qPCR analysis to examine expression of various cytokines. To enable LAM purification, we eliminated the capsule prior to digesting the tissue. Fitting with the GO analysis, LAMs expressed more *Il1b* at steady state compared with KCs (Figure 2L). However, despite this, upon *in vivo* TLR4 stimulation, they were less responsive than KCs, both in terms of pro- and anti-inflammatory cytokines (Figure 2L), possibly indicative of LPS tolerance. This may result from their location at the PV and hence exposure to blood from the intestine, although this remains to be tested. Taken together this highlights the distinct nature and functions of these cells; however, further research is required to determine the precise roles of these cells.

### Macrophage subsets reside in distinct spatial niches

**Figure 3:**
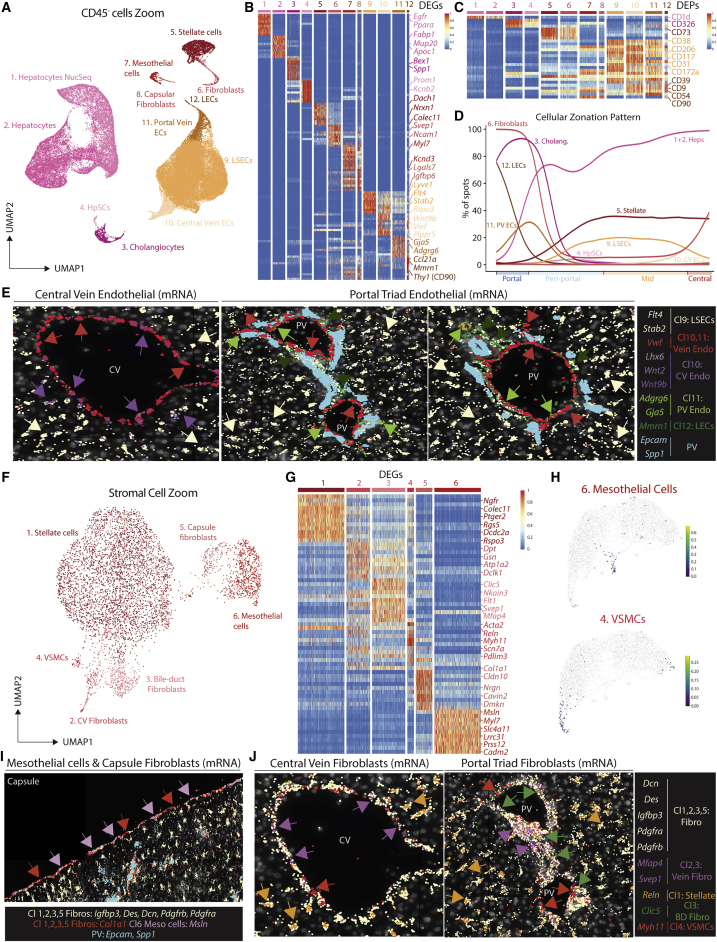
Hepatic macrophage populations reside in distinct niches (A) UMAP of murine CD45^−^ cells (83,410 cells/nuclei) isolated from Figure 1B and re-clustered with TotalVI. (B and C) Top DEGs (B) and DEPs (C) between cell types. (D) Indicated cell signatures from sc/snRNA-seq mapped onto the Visium zonation data. (E) Molecular Cartography of indicated genes at central vein (left) and 2 different portal triads (center and right). (F) UMAP of murine stromal cells (5,430 cells/nuclei) isolated from the UMAP in Figure 3A and re-clustered with scVI. (G) Top DEGs between different cell types identified. (H) Identification of mesothelial cell (top) and VSMC (bottom) signatures on zonated Visium data. (I and J) Molecular Cartography of indicated genes at the liver capsule (I) or the central vein (J; left) and portal triad (J; right). PV, portal vein; CV, central vein; HA, hepatic artery; BD, bile duct. Arrows indicate specific cell types, where color corresponds to markers. Images are representative of 2–4 mice. See also Figure S4 and Tables S3 and S4.

As all the mac populations are in close contact with CD45^−^ cells in their local environment (Bonnardel et al., 2019; Figure S3M), we further analyzed the CD45^−^ cells, identifying multiple subsets of ECs and SCs and a gating strategy to distinguish them (Figures 3A–3C and S4A–S4C; Table S3). ECs could be further subdivided into 4 distinct clusters and analysis of their locations allowed them to be identified as CV ECs (cluster10), LSECs (cluster9), PV ECs (cluster11), and lymphatic ECs (LECs; cluster12) (Figures 3D, 3E, S4D, and S4E). As Visium found fibroblasts at both the PVs and CVs (Figure 3D), and as a previous report has suggested the presence of distinct subsets within these cells (Dobie et al., 2019), we further zoomed in on the SCs to better assess their heterogeneity (Figures 3F, 3G, S4A–S4C, and S4F; Table S4). This revealed subsets of mesothelial cells and fibroblasts restricted to the capsule (Figures 3H and 3I). *Myh11*^+^ vascular smooth muscle cells (VSMCs) were localized around hepatic arteries, PVs and CVs (Figures 3F–3H, 3J, and S4G) and *Mfap4*^*+*^*Svep1*^*+*^*Clic5*^*−*^*Reln*^*−*^ fibroblasts were found to be CV fibroblasts (Figures 3G, 3H, 3J, and S4G). Finally, we identified a subset of *Clic5*^*+*^*Reln*^*+*^ fibroblasts (cluster3), which were localized around the cholangiocytes, that we termed bile-duct fibroblasts (Figures 3J and S4G). Taken together, the presence of these spatially distinct subsets of ECs and SCs highlights the uniqueness of the specific microenvironments in which the distinct mac populations reside.

### An *in silico* gating strategy for murine hepatic lymphoid cells

**Figure S5:**
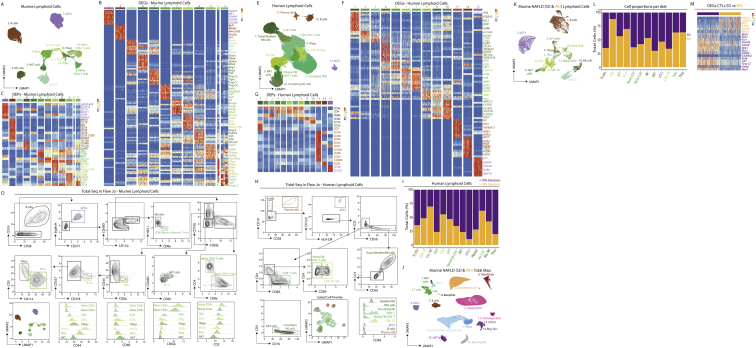
Combination of CITE-seq, scRNA-seq, snRNA-seq, and spatial analyses enables generation of a human liver atlas, related to Figure 4 (A) Murine lymphoid cells (B cells, T cells, NK cells, ILC1s, pDCs; 27,398 cells) were isolated from Figure 1B and re-clustered with TotalVI. (B and C) Top DEGs (B) and DEPs (C) for the cell types from Figure S5A. (D) CITE-seq data from Figure S5A were exported as an FCS file and an *in silico* gating strategy identified in FlowJo. (E) Human lymphoid cells (B cells, T cells, NK cells, ILC1s, pDCs; 105,790 cells) were isolated from Figure 4B and re-clustered with TotalVI. (F and G) Top DEGs (F) and DEPs (G) for the cell types from Figure S5E. (H) CITE-seq data from Figure S5E were exported as an FCS file and an *in silico* gating strategy identified in FlowJo. (I) Proportion of indicated cell types arising from patients with <10% (purple) or >10% steatosis (yellow). (J) Hepatic cells were isolated from 22 C57B/l6 mice fed either a standard diet (SD) or a western diet (WD) for 24 or 36 weeks to induce NAFLD and NASH by *ex vivo* (10 samples) or *in vivo* (12 samples) enzymatic digestion. Alternatively, livers were snap frozen and nuclei isolated by tissue homogenization (14 samples). Live cells/intact nuclei were purified using FACS. For cells, total live, live CD45^+^, live CD45^−^, live hepatocytes or myeloid cells (live CD45^+^, CD3^−^, CD19^−^, B220^−^, NK1.1^−^) were sorted. 10 samples were also stained with a panel of 107–161 barcode-labeled antibodies for CITE-seq analysis. All datasets were pooled together and after QC 121,980 cells/nuclei were clustered using TotalVI. (K) Murine lymphoid cells (B cells, T cells, NK cells, ILC1s, pDCs; 21,322 cells) from mice fed the SD or WD for 24 or 36 weeks were isolated from Figure S5J and re-clustered with TotalVI. (L) Proportion of indicated cell types arising from mice fed the SD (purple) or WD (yellow). (M) Top DEGs between CTLs isolated from mice fed the SD (purple) or WD (yellow).

Finally, in addition to providing gating strategies for myeloid and CD45^−^ cells, we also wanted to investigate if the CITE-seq data would allow us to develop similar strategies for the lymphoid populations. To examine this, we re-clustered the T cells, NK cells, ILCs, B cells, and pDCs from Figure 1B identifying 12 distinct populations (Figure S5A). Analysis of the DEGs highlighted that while B cells, NK cells, ILC1s, and pDCs were distinct populations, there was considerable overlap between the transcriptomic profiles of the T cells (Figure S5B; Table S5). However, by analyzing the DEPs we were able to define distinct subsets including naive CD4 and CD8 T cells, T_Regs_, T_H_1s, CTLs, and T_H_17s (Figures S5A and S5C; Table S5). Moreover, we were able to design a gating strategy to isolate the distinct populations (Figure S5D).

### A practical proteogenomic atlas of the healthy human liver

**Figure 4:**
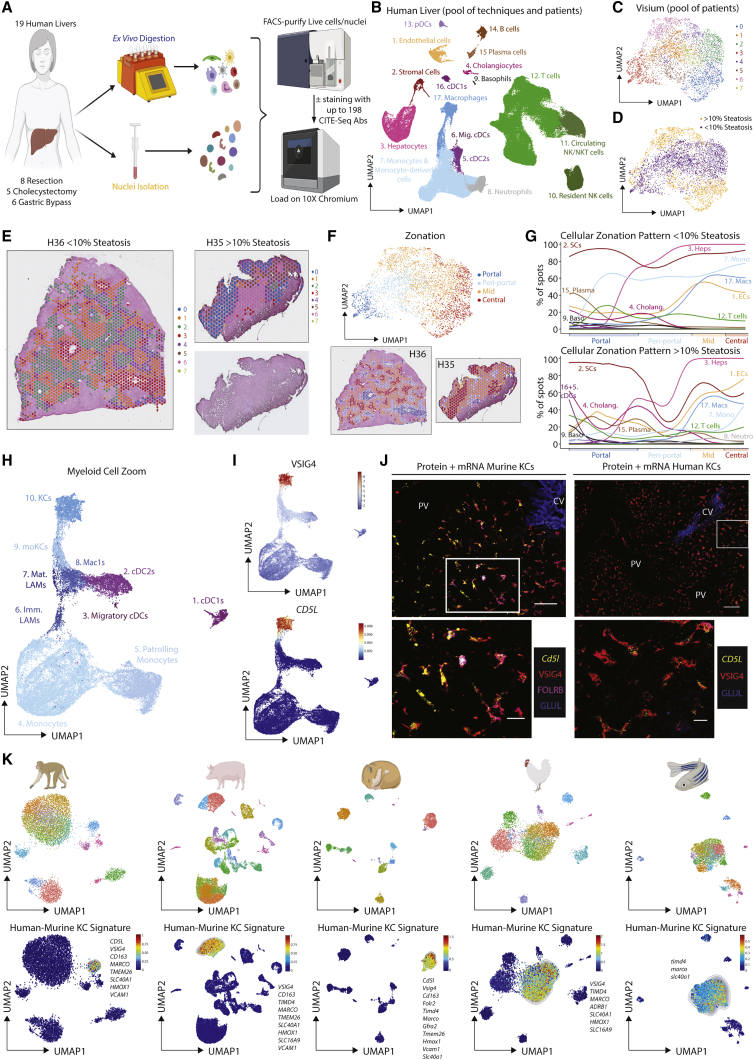
Identification of bona fide Kupffer cells across species (A) Cells/nuclei were isolated from liver biopsies (∼1–2 mm^3^; 14 cells, 5 nuclei) from patients undergoing either liver resection, cholecystectomy or gastric bypass. Live cells/intact nuclei were FACS-purified. Either total live, live CD45^+^, and live CD45^−^ or live CD45^+^, CD3^−^, and CD19^−^ cells were sorted. 7 cell samples were stained with a panel of 198 barcode-labeled antibodies for CITE-seq analysis. All datasets were pooled together and after QC, 167,598 cells/nuclei were analyzed using TotalVI. (B) UMAP of sc/snRNA-seq data. (C) UMAP of Visium data from 4 patient biopsy samples. (D) Split of Visium spots based on % steatosis. (E) Healthy and steatotic Visium liver tissue with clusters overlaid and H+E staining to identify steatotic zones. (F) Zonation of Visium data (top) with zonation pattern mapped onto liver tissue (bottom). (G) Indicated cell signatures from sc/snRNA-seq mapped onto Visium zonation trajectory, healthy (top), steatotic (bottom). (H) Myeloid cells (40,821 cells) were isolated from Figure 4B and re-clustered with TotalVI. (I) Expression of VSIG4 protein (top) and *CD5L* mRNA (bottom). (J) Expression of VSIG4, F4/80, FOLRB, and GLUL combined with *Cd5l/CD5L* on murine (left) and human (H25; right) livers. Scale bars, 50 μm. Inset in bottom panels. Scale bars, 20 μm. Images are representative of 2–4 livers. (K) Livers (2/species) were isolated from healthy macaque, pig, chicken, hamster, and zebrafish. Cells were isolated by *ex vivo* digestion for CITE-seq (pig; 198 human antibodies) or scRNA-seq (hamster, chicken, and zebrafish), or nuclei were isolated for snRNA-seq (macaque). Total live cells (hamster, chicken, and pig), DsRed^+^GFP^+^ cells (zebrafish) or nuclei (macaque) were FACS-purified. Following QC, 8,483 nuclei (macaque) or 21,907 (pig), 5,965 (hamster), 7,457 (chicken), and 4,957 (zebrafish) cells were analyzed using TotalVI (pig) or scVI (macaque, hamster, chicken, and zebrafish) (top). KCs were identified using the human-murine KC signature and the signature finder algorithm (Pont et al., 2019) (bottom). See also Figures S6 and S7 and Tables S1, S2**,**S3, S5, S6**,**S8, and S9.

**Figure S6:**
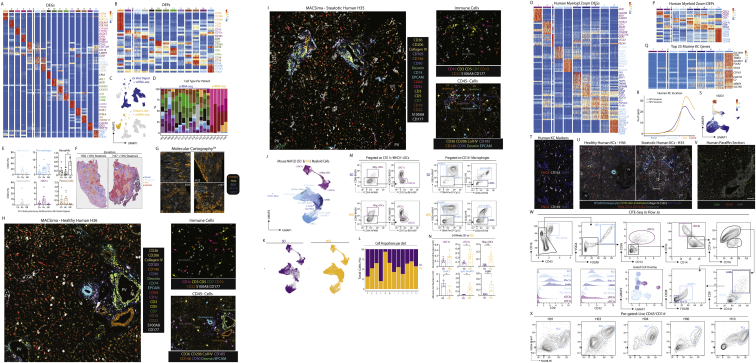
Combination of CITE-seq, scRNA-seq, snRNA-seq, and spatial analyses enables generation of a human liver atlas and identification of bona fide human KCs, related to Figure 4 (A and B) Top DEGs (A) and DEPs (B) for the cell types from Figure 4B. (C) Distinct profiles of cells or nuclei within the UMAP depending on isolation protocol used; 152,535 cells from *ex vivo* digestions and 15,063 nuclei. (D) Proportion of each cell type per patient profiled. (E) Proportion of indicated cell types as a % of total CD45^+^ cells calculated from *ex vivo* digested samples per surgery type. Ch; cholecystectomy, Re; resection, GB; gastric bypass. ^∗^p < 0.05; one-way ANOVA with Bonferroni post-test. (F) Mapping of Visium UMAP zonation patterns onto tissue sections from patient H35 and H37. (G) Expression of indicated zonation genes in patients H35–H38 assessed by Molecular Cartography. (H and I) Expression of indicated proteins by MICS 100-plex protein analysis in the healthy (H) and steatotic (I) human liver. (J) Murine myeloid cells (cDC1s, cDC2s, Mig. cDCs, Macs, monocytes, and monocyte-derived cells; 42,922 cells) from mice fed the SD or WD for 24 or 36 weeks were isolated from Figure S5J and re-clustered with TotalVI. (K) Distribution of cells in UMAP originating from SD- (purple) or WD- (yellow) fed mice. (L) Proportion of indicated cell types arising from mice fed the SD (purple) or WD (yellow). (M and N) Flow cytometry analysis of indicated cell populations in SD and WD-fed mice (24 weeks). Representative gating strategies (M) and absolute number of indicated populations (N). ^∗^p < 0.05, ^∗∗^p < 0.01 Student’s t test. Data are from 2 independent experiments with n = 5–6 per diet. (O and P) Top DEGs (O) and DEPs (P) for cell types from Figure 4H. (Q) Top 25 Murine KC genes as expressed by the human myeloid cell clusters. (R) Mapping of KC signature onto Visium trajectory for healthy (purple) and steatotic (orange) livers. (S) Expression of VSIG4 mRNA within human myeloid cells. (T) Expression of VSIG4 (red) and CD163 (gray, top) or CD169 (gray, bottom) by MICS analysis in healthy human liver. (U) Representative images showing KC location (red) as assessed by MICS analysis in the healthy (left) and steatotic (right) human liver. PV, portal vein; CV, central Vein, dashed line indicates zones of steatosis. (V) Representative image of CD68 and CD163 staining in 10–15-year-old human liver paraffin sections. Image is representative of 6 different patients. (W) *In silico* gating strategy to isolate distinct myeloid cell populations identified from CITE-seq data. (X) Expression of VSIG4 and FOLR2 by live CD45^+^ cells also expressing CD14 in indicated human liver biopsies by flow cytometry. Data are representative of 21 biopsy samples analyzed.

To determine the degree of conservation between the mac subsets and their different microenvironmental niches between the mouse and the human liver, we next generated a proteogenomic atlas of the human liver using sc/snRNA-seq and CITE-seq on 19 liver biopsies (Figures 4A, 4B, S6A, and S6B; Tables S1 and S5). Of these, most were histologically healthy with only 5 patients showing >10% hepatic steatosis in the absence of any significant fibrosis (Table S6). Cellular proportions varied according to the isolation technique used, and while there was some variability between patients, this was not linked to the surgery (Figures S6C–S6E). Further confirming the lack of fibrosis in the steatotic livers, we did not detect any increase in CTLs (Figures S5E–S5I; Table S5), which have been shown to correlate with non-alcoholic steatohepatitis (NASH) (Haas et al., 2019). A significant increase in CTLs was detected in the setting of murine NASH induced by feeding a western diet (WD) for up to 36 weeks, demonstrating that this is not due a limitation in detecting these differences using CITE-seq (Figures S5J–S5M; Table S7). As Visium reliably located murine hepatic cells, we used this to locate the cells of the human liver in 4 biopsies (Figure 4C). However, the Visium spots from patients with >10% steatosis were found to cluster separately from the healthy samples (<10% steatosis; Figures 4D and 4E). We therefore used the healthy samples to calculate a baseline zonation and then transferred this trajectory onto the steatotic samples (Figures 4F and S6F). This identified the steatosis to be predominantly present in regions expressing peri-central zonation genes like *CYP2E1*. This zonation pattern was further validated using Molecular Cartography (Figure S6G). This fits with previous clinical studies demonstrating peri-central steatosis to be most common in non-alcoholic fatty liver disease (NAFLD) patients, especially in early disease (Chalasani et al., 2008; Kleiner and Makhlouf, 2016). However, as these peri-central regions are larger than in the healthy controls, it could also imply that the presence of steatosis alters expression of the zonated hepatocyte genes, but this remains to be tested. Notably, the overall cellular distribution was not impacted by the presence of steatosis, although neutrophils and monocytes and monocyte-derived cells were preferentially localized peri-centrally in the steatotic patients correlating with the presence of steatosis (Figure 4G). MICS 100-plex protein analysis further validated the cellular distributions predicted by Visium and confirmed the increase in neutrophils in the steatotic livers (Figures S6H and S6I).

### Evolutionarily conserved transcriptomic and proteomic identity of KCs

**Figure S7:**
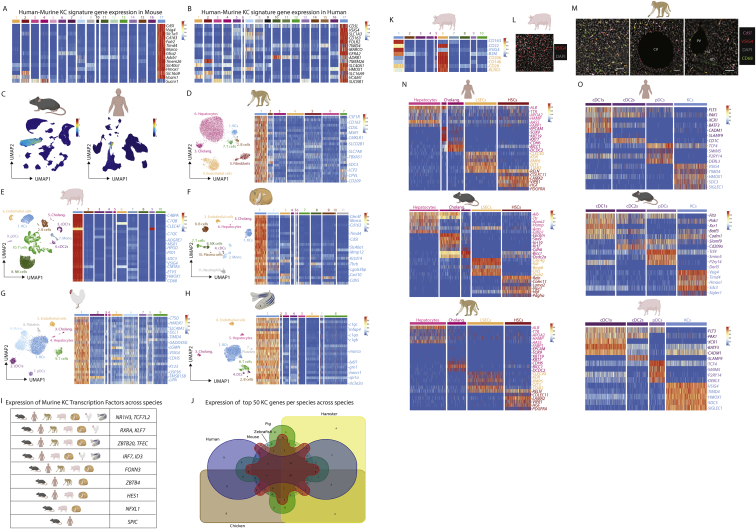
Conserved and unique features of KCs across species, related to Figure 4 (A and B) Expression of human-murine KC signature genes across cell types in mouse (A) and human (B). (C) Unbiased identification of KCs in mouse and human using the human-murine KC signature and the signature finder algorithm (Pont et al., 2019). (D–H) Annotated UMAPs from indicated species and expression of top KC-specific genes compared with other cells per species. (I) Expression of previously identified core murine transcription factors (Bonnardel et al., 2019) by KCs across species. (J) Venn diagram showing convergence and divergence of expression of top 50 KC genes per species across species, see Table S9 for genes lists per species. (K) Top DEPs (identified with cross reactive human antibodies) in the pig CITE-seq data. (L) Expression of VSIG4 in the porcine liver by confocal microscopy. (M) Expression of VSIG4, CD68 (protein), and *CD5L* (mRNA) in macaque liver. PV, portal vein; HA, hepatic artery; BD, bile duct. All images are representative of 2 livers. (N and O) Conserved expression of indicated genes across CD45^−^ (N) and CD45^+^ (O) cell types and species.

To date, no validated markers of bona fide human KCs have been described. Explaining the difficulty to accurately define human KCs, we found monocytes and macs formed a single continuum in the human sc/snRNA-seq data, preventing a simple definition of human KCs (Figure 4B). Notably, a similar continuum from monocyte to KCs was also observed in the NASH murine liver (Figures S5J and S6J–S6N, Table S8). Consistent with our previous report (Remmerie et al., 2020), we observed both long-term resident *Timd4*-expressing KCs and recently recruited *Timd4*^*−*^ monocyte-derived KCs (moKCs; Figures S6J–S6N). As the presence of such a continuum in the human liver suggests that there may also be monocyte contribution to the KC pool in the healthy human liver, we next zoomed in on myeloid cells to examine this, identifying 10 clusters (Figures 4H, S6O, and S6P; Table S2). To define the KCs, we examined expression of the top 25 murine KC genes by these clusters, which identified cluster10 to be the genuine human KCs (Figure S6Q). Unlike in mice, these were preferentially located in the mid zone (Figure S6R). Cluster9 also expressed many of these genes but lacked *TIMD4* (Figure S6Q), suggesting that these cells may be recently recruited moKCs. The presence of moKCs in the liver is consistent with reports that host-derived macs are identified in transplanted donor livers (Bittmann et al., 2003; Pallett et al., 2020) and suggests that the KC population may be a mix of embryonic and monocyte-derived cells. Although not the case at mRNA level, VSIG4 was found to be the best human KC protein marker in the CITE-seq data, while FOLR2, CD163, and CD169 were also identified as useful markers of these cells for flow cytometry and confocal microscopy on frozen and paraffin sections (Figures 4I and S6S–S6X). Co-staining human livers for VSIG4 protein and KC-specific *CD5L* mRNA and MICS 100-plex protein analysis also confirmed the mid-zonal localization of KCs (Figures 4J and S6U). To assess if KC identity was further conserved in evolution, we profiled macaque, pig, hamster, chicken, and zebrafish livers (Figure 4K). We identified the KCs in an unbiased manner by mapping the conserved human-mouse KC signature onto the datasets (Figures 4K and S7A–S7C). We then examined the main features of each KC population identified (Figures S7D–S7H; Table S9). A strong overlap in transcriptomes across species was observed likely due to the conserved expression of core KC transcription factors (Figure S7I). However, each species also harbored a number of unique KC genes (Figure S7J; Table S9). VSIG4 protein expression was also conserved in pig and macaque KCs (Figures S7K–S7M). Similarly, we were also able to identify most of the other hepatic cells across species on the basis of conserved genes (Figures S7N and S7O). cDC2s were the main exception to this, as specific cDC2 marker genes were not conserved across all species (Figure S7O).

### LAM location is altered in the steatotic liver

**Figure 5:**
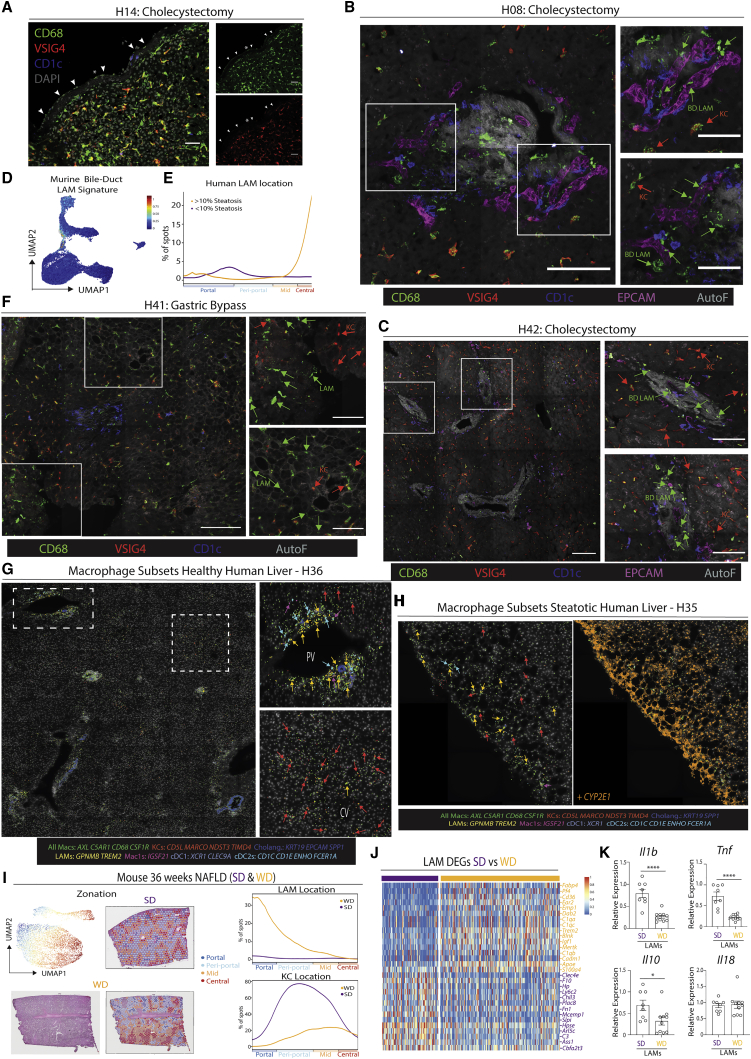
LAMs are found at the bile duct in the healthy liver, but at zones of steatosis in the obese liver (A) Expression of indicated markers at the capsule of the healthy human liver (H14) by confocal microscopy. Scale bars, 20 μm. Arrowheads indicate capsule macs. (B and C) Representative images showing expression of indicated markers at the portal triad by confocal microscopy. Insets on right. Scale bars, 100 μm. Insets scale bars, 50 μm (B). Scale bars, 150 μm. Insets scale bars, 75 μm (C). (D) Human bile-duct LAMs identified using the murine bile-duct LAM gene signature and the signature finder algorithm (Pont et al., 2019). (E) Human LAM signature from scRNA-seq mapped onto the Visium zonation data, healthy (purple), steatotic (yellow). (F) Expression of indicated markers by confocal microscopy. Insets on right. Scale bars, 150 μm. Insets scale bars, 75 μm. (G and H) Expression of indicated genes in healthy (G) and steatotic (H) human liver. Insets on right. Images are representative of 2 patients per condition. (I) Mice were fed a western diet (WD) or standard diet (SD) for 36 weeks to induce NAFLD and Visium analysis was performed. Analysis is pooled from 1 liver slice from the SD condition and 3 liver slices from the WD condition. Zonation pattern and H&E staining (left) and LAM and KC location (right). (J) Heatmap showing DEGs between LAMs from SD (purple) and WD (yellow) fed mice (24 + 36 weeks pooled). (K) LAMs were FACS-purified from the liver of mice fed the SD or WD for 24 weeks (with removal of capsule prior to digestion), RNA was isolated and expression of indicated genes was assessed by qPCR relative to β-actin. ^∗^p < 0.05, ^∗∗∗∗^p < 0.0001, Student’s t test. Data are pooled from 2 independent experiments with n = 7–10 mice per group. Images from (A–C and F) are representative of 4–5 patients per condition. PV, portal vein; CV, central vein. See also Figure S8 and Table S2.

**Figure S8:**
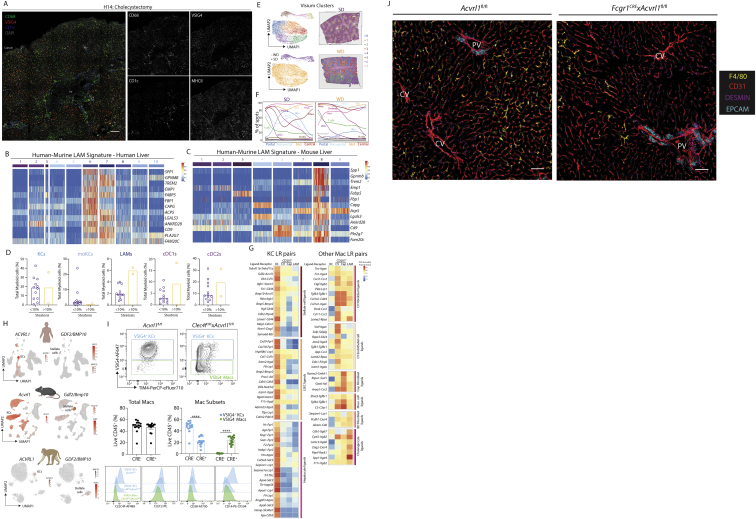
Evolutionarily conserved signals regulate LAM and KC development, related to Figures 5 and 7 (A) Confocal microscopy of healthy human liver showing expression of indicated markers. Scale bars, 200 μm. (B and C) Expression of conserved human-murine bile-duct LAM signature in human (B) and mouse (C) hepatic myeloid cells. (D) Proportion of indicated myeloid cell populations as a % of total myeloid cells in human liver biopsies profiled by scRNA-seq when divided based on presence of steatosis. (E) Mice were fed a western diet (WD) or standard diet (SD) for 36 weeks to induce NAFLD and Visium analysis was performed. Analysis is pooled from 1 liver slice from the SD condition and 3 liver slices from the WD condition. Shown are cluster and sample annotations. (F) Zonation of all cell types from Figure S5J in murine NAFLD map (SD&WD). (G) Differential NicheNet highlighting prioritized conserved (human-mouse) ligand-receptor (LR) pairs between indicated macs and their niche cells. LR pairs are grouped according to the niche cell type with highest ligand expression. (H) Expression of ALK1 (ACVRL1), BMP9 (*GDF2*), and *BMP10* in human, mouse, and macaque livers where both KCs and stellate cells were profiled. (I) Livers were harvested from *Clec4f*-Crex*Acvrl1*^fl/fl^ mice or *Acvrl1*^*fl/fl*^ controls and KCs examined (top) and quantified (middle) using VSIG4 expression. Expression of indicated KC markers by mac populations in *Clec4f*-Crex*Acvrl1*^fl/fl^ or *Acvrl1*^*fl/fl*^ control mice (bottom). Data are pooled from 2 independent experiments with n = 14 per group. Student’s t test. ^∗∗∗∗^p < 0.0001. (J) Expression of CD31 (ECs), DESMIN (stromal cells), F4/80 (Macs), and EPCAM (cholangiocytes) by confocal microscopy in *Fcgr1*-*Cre*x*Acvrl1*^fl/fl^ mice and Acvrl1^+/+^ controls. PV, portal vein; CV, central vein. Images are representative of 2 mice per group.

Alongside KCs, we also identified distinct clusters of macs in the human myeloid cells (Figure 4H). To better understand the nature of these clusters we performed confocal microscopy to examine the specific locations of CD68^+^VSIG4^−^ macs in the human liver (Figure S8A). This identified CD68^+^VSIG4^−^ macs in the liver capsule, in close proximity to central and PVs as well as at bile ducts (BDs) (Figures 5A–5C and S8A). Similar populations were also observed at the PVs and CVs and at the BDs in the healthy macaque liver (Figure S7M). Examination of the scRNA-seq data and comparison with murine signatures identified immature and mature LAMs, with immature LAMs expressing some monocyte genes (Figures 4H, 5D, S8B, and S8C). Although recently suggested to be specific to fibrotic human livers (Ramachandran et al., 2019), we identified LAMs in all patients profiled with scRNA-seq, but there was a trend toward increased proportions of LAMs in the livers with >10% steatosis (Figure S8D) consistent with the increased population of LAMs in murine NAFLD (Figures S6J–S6N). As in the healthy mouse, Visium identified human LAMs in portal zones of non-steatotic livers. However, in steatotic human livers, LAMs were primarily located peri-centrally, in zones with steatosis (Figure 5E), suggesting that monocytes are recruited to distinct locations in the healthy and obese liver where they then differentiate into LAMs. This altered location of LAMs was further validated by confocal microscopy and Molecular Cartography (Figures 5F–5H). However, this analysis did not identify any capsule macs, suggesting that these cells may be absent from our UMAP, likely as a result of the small amount of capsule tissue on a biopsy. The Mac1 population expressing *IGSF21* was present in very low numbers throughput the tissue (Figures 5G and 5H). This coupled with their similar transcriptomic profile to moKCs could suggest that these are moKC precursors as observed in the mouse, but this requires further study. Focusing on the LAMs, the change in their location in the steatotic human liver was also observed in the murine NAFLD model. Here, LAMs were found across portal, periportal, and mid zones (Figures 5I, S8E, and S8F), fitting with the presence of steatosis in these regions and consistent with our previous report (Remmerie et al., 2020). Comparison of DEGs between LAMs in standard diet (SD) and WD-fed mice identified that LAMs had a more mature phenotype in WD-fed mice, downregulating their expression of some monocyte genes and increasing their expression of prototypical mac markers, consistent with the presence of both immature and mature LAMs in the human liver (Figure 5J). Fitting with a more mature phenotype, WD-derived LAMs also expressed lower levels of *Il1b, Tnf*, and *Il10* compared with SD LAMs (Figure 5K). While further studies are required to assess the precise functions of these cells in NAFLD, this could further suggest a protective rather than a pathogenic role for LAMs (Daemen et al., 2021).

### Potential role for CD45^−^ hepatic cells in determining macrophage localization

**Figure 6:**
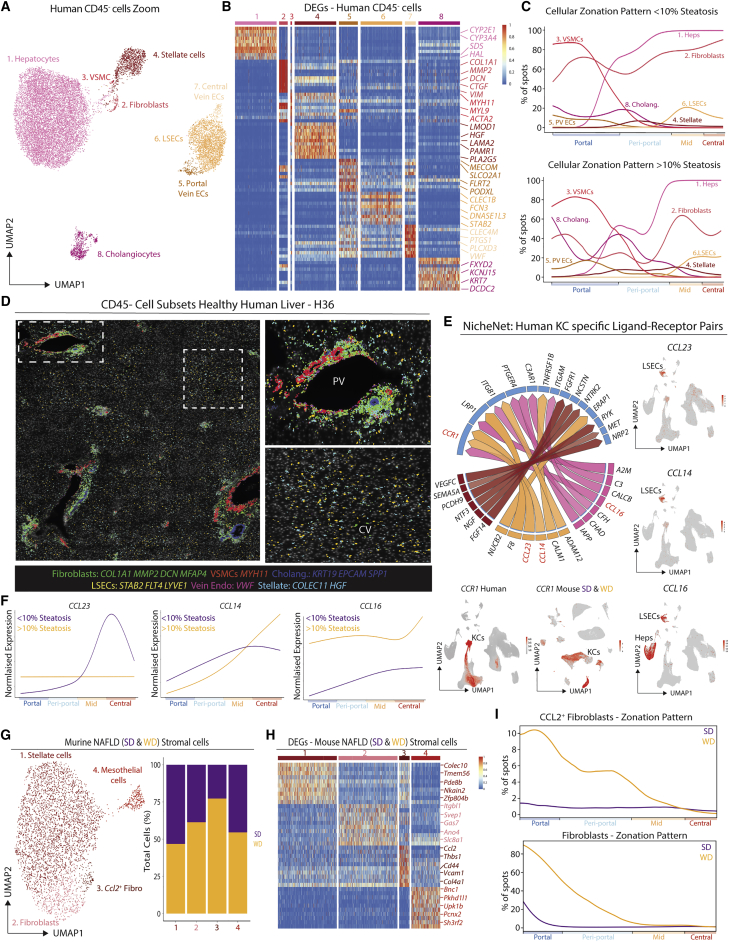
Macrophage niche cells may regulate macrophage locations in healthy versus steatotic liver (A) UMAP of human CD45^−^ cells (15,481 cells/nuclei) isolated from Figure 4B and re-clustered with scVI. (B) Top DEGs between cell types identified. (C) Indicated cell signatures from sc/snRNA-seq mapped onto the Visium zonation data, healthy (top), steatotic (bottom). (D) Molecular Cartography localizing distinct CD45^−^ cells. (E) Circos plot showing NicheNet predicted ligand-receptor pairs between KCs and LSECs, HSCs and hepatocytes uniquely expressed in the human liver (left) and UMAPs showing normalized expression of indicated chemokines in the human liver (right) and *CCR1/Ccr1* in the human and murine NAFLD liver (bottom). (F) Zonation of indicated genes in the healthy and steatotic human liver. (G) UMAP of murine NAFLD (SD and WD) stromal cells (4,025 cells/nuclei) isolated from Figure S5J and re-clustered with scVI (left) and proportions of each cell type in SD- and WD-fed mice (right). (H) Top DEGs between cell types. (I) Mice were fed a western diet (WD) or standard diet (SD) for 36 weeks to induce NAFLD, and Visium analysis was performed. Analysis is pooled from 1 liver slice from the SD condition and 3 liver slices from the WD condition. Zonation of *Ccl2*^+^ fibroblasts and fibroblasts in SD- and WD-fed mice. See also Tables S3 and S10.

Given the altered localization of KCs in the healthy murine versus human liver and LAMs in the healthy versus steatotic liver of mice and humans, we next sought to investigate how the cells of the mac niches differed in these settings. Analysis of CD45^−^ cells in the human liver identified similar (sub)populations of ECs, SCs, and hepatocytes as observed in the healthy mouse liver (Figures 6A and 6B; Table S3). However, while the 100-plex MICS analysis detected a second subset of portal fibroblasts expressing DESMIN (Figure S6H), we did not detect these cells in our UMAP, likely due to difficulties detecting DESMIN with snRNA-seq. No significant differences were found in terms of localization of the identified CD45^−^ cells that could explain the altered location of KCs compared with the mouse (Figures 6C and 6D). With this in mind, we next utilized NicheNet to examine potential ligand-receptor pairs between KCs and CD45^−^ cells present only in the human that could regulate KC location. This analysis identified *CCL23* and *CCL14* expression by LSECs and *CCL16* by hepatocytes that binds to *CCR1* expressed by human KCs but not murine KCs (Figure 6E). Crucially, these ligands were preferentially located peri-centrally (Figure 6F) and thus represent interesting targets for further study regarding the potential regulation of KC location in the human liver.

We next aimed to investigate the role of CD45^−^ cells in the regulation of LAM location in the healthy versus steatotic liver. Given the low number of steatotic human samples in our sc/snRNA-seq analysis and the variation between patients, we turned to the murine NASH model to investigate this. Zooming in on SCs (Figure 6G; Table S10), we identified that there was an increase in fibroblasts in the WD-fed mice. There was also a considerable overlap between fibroblasts and HSCs in terms of their gene expression profiles suggesting there may be a differentiation trajectory between the HSCs and the fibroblasts, although this remains to be validated *in vivo*. We also noted a sub-population of fibroblasts expressing *Ccl2*, a known ligand for CCR2 which is expressed on monocytes recruited to the liver during NAFLD (Remmerie et al., 2020), as well as *Cd44* and *Vcam1*, two genes involved in monocyte recruitment and adhesion (Johnson and Ruffell, 2009; Meerschaert and Furie, 1995). Both fibroblast populations were enriched in the periportal steatotic regions (Figure 6I), in which we also observe enrichment of the LAMs (Figure 5I). We also found high *CCL2* and *Cd44* expression in human fibroblasts (Table S3), potentially recruiting bile-duct LAMs. Together, this suggests that fibroblasts may play an important role in recruiting LAMs.

### Differential NicheNet analysis across species reveals a crucial role for the ALK1-BMP9/10 axis in KC development

**Figure 7:**
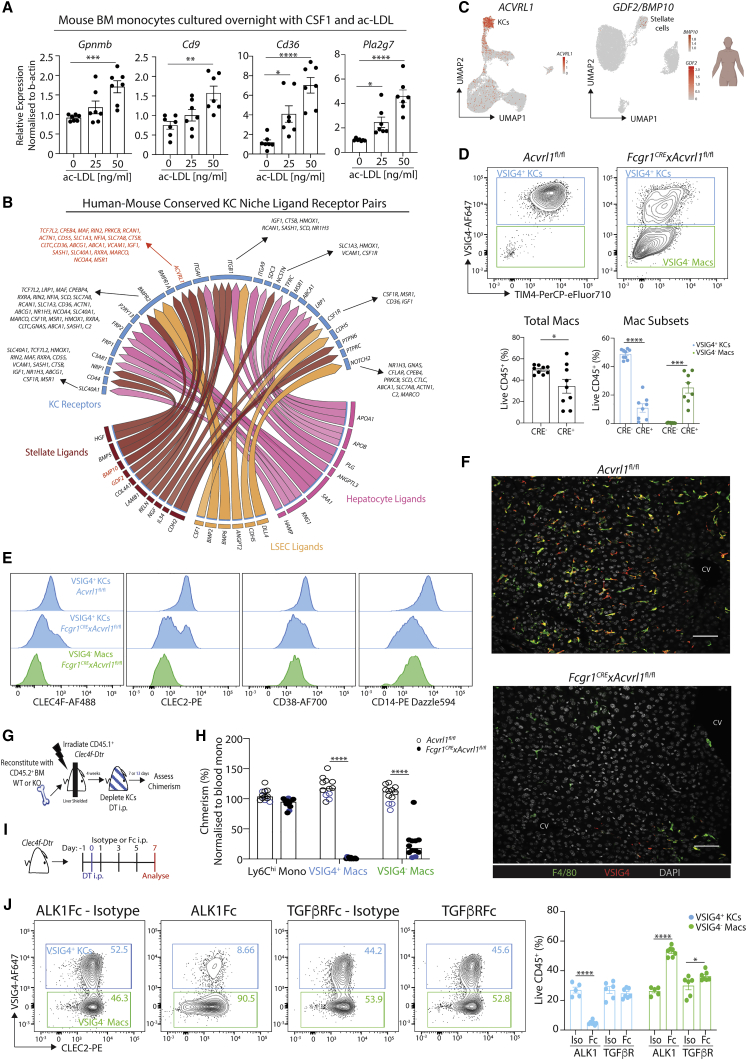
ALK1-BMP9/10 axis regulates KC development (A) Mouse BM monocytes were cultured in the presence of CSF1 and indicated concentrations of human ac-LDL, prior to analyzed for expression of indicated genes by qPCR. Data are pooled from 2 experiments. One-way ANOVA with Bonferroni post-test compared with 0 ng/mL. (B) NicheNet circos plot highlighting conserved ligand-receptor pairs and induced target genes between KCs and indicated niche cells in human and mouse. (C) Feature plots showing expression of ALK1 (*Acvrl1*) in human myeloid cells (left) and *GDF2/BMP10* in CD45^−^ cells (right). (D) Livers were harvested from *Fcgr1*-Crex*Acvrl1*^fl/fl^ mice or *Acvrl1*^*fl/fl*^ or *Acvrl1*^*+/+*^ controls and KCs examined (left) and quantified (right) using VSIG4 expression. (E) Expression of indicated KC markers by mac populations in *Fcgr1*-Crex*Acvrl1*^fl/fl^ or *Acvrl*^*fl/fl*^ control mice. Data are pooled from 3 independent experiments with n = 9 per group. Student’s t test. (F) Expression of indicated markers in livers of *Fcgr1*-Crex*Acvrl1*^fl/fl^ or *Acvrl1*^*fl/fl*^ or *Acvrl1*^*+/+*^ control mice by confocal microscopy. Scale bars, 50 μm. Images are representative of 2 mice per group. (G) Schematic of chimera experiment setup. (H) % chimerism normalized to levels in blood Ly6C^hi^ monocytes in *Clec4f-Dtr* mice 7 or 13 days after DT administration following partial irradiation and receiving either *Acvrl*^*fl/fl*^ or *Fcgr1-CrexAcvrl*^*fl/fl*^ BM 4 weeks earlier. Data are pooled from 2 independent experiments with n = 10–12 mice per group. (I) Schematic of Fc trap experiment setup. (J) Representative FACS plots showing VSIG4 and CLEC2 expression by total macs. Numbers represent % of total mac population in the indicated gate (left) and % of VSIG4^+^ and VSIG4^−^ macs among total CD45^+^ cells in the different treatment conditions. One-way ANOVA with Bonferroni post-test. ^∗^p < 0.05, ^∗∗^p < 0.01, ^∗∗∗^p < 0.001, ^∗∗∗∗^p < 0.0001. See also Figure S8.

Having identified a potential role for the mac niche cells in regulating mac subset location, we next sought to determine the involvement of these cells in regulating mac phenotypes. To assess the roles of conserved cell-cell interactions in driving mac heterogeneity across species, we performed a differential NicheNet (Browaeys et al., 2019) analysis between the distinct hepatic macs and the CD45^−^ cells present in their respective niches focusing on ligands and receptors conserved in both human and mouse. This revealed very few specific ligand-receptor pairs for LAMs (Figure S8G; data not shown), hinting that local factors such as metabolites rather than unique cell-cell interactions may drive the LAM phenotype. Indeed, this would be consistent with the presence of LAM-like cells in multiple tissues including the obese adipose tissue, the brain, the lung, and the heart (Jaitin et al., 2019; Keren-Shaul et al., 2017; Liao et al., 2020; Rizzo et al., 2020). In line with this, BM monocytes cultured with acetylated low-density lipoprotein expressed LAM-associated genes (Figure 7A), demonstrating a dominant role for lipids in inducing the LAM phenotype. Conversely, for KCs, we found multiple ligand-receptor pairs to be conserved between human and mouse (Figures 7B and S8G). One of these, an activin receptor-like kinase (ALK1)-bone morphogenic protein (BMP)9/10 circuit between KCs (ALK1; encoded by *Acvrl1*) and stellate cells (BMP)9/10 encoded by *Gdf2/Bmp10* respectively) was found to be conserved in all 7 species and was predicted to control the expression of a number of the conserved KC genes (Figures 7B, 7C, and S8H). To validate a role for this axis in KCs, we generated *Fcgr1*-Cre × *Acvrl1*^fl/fl^ mice, eliminating ALK1 specifically from CD64-expressing macs (Scott et al., 2018). This led to an almost complete loss of VSIG4^+^ KCs (Figures 8D–8F), demonstrating that evolutionarily conserved ALK1 signaling is crucial for KCs. To determine if ALK1 is required for KC maintenance we generated *Clec4f-*Cre × *Acvrl1*^fl/fl^ mice, eliminating ALK1 only from differentiated KCs (Scott et al., 2018). This revealed a relatively similar phenotype than observed in the *Fcgr1-Cre* mice, suggesting that ALK1 is also required for KC maintenance (Figure S8I). To directly test the need for ALK1 in KC development, we generated BM chimeras whereby CD45.1 *Clec4f-Dtr* mice were irradiated with their livers shielded to avoid any radio damage. These mice were then reconstituted with either CD45.2 *Acvrl1*^fl/fl^ or *Fcgr1-CrexAcvrl1*^fl/fl^ BM. 4 weeks later, mice were given a single i.p. injection of DT to deplete the KCs and 7 or 13 days thereafter chimerism within the KC population was determined (Figure 7G). We observed that, already by day 7, KO BM-derived cells were almost completely outcompeted by WT BM-derived cells within the KC pool. This was not observed in monocytes but a similar pattern was observed in VSIG4^−^ macs demonstrating that ALK1 is critically required for early KC development (Figure 7H). Finally, while NicheNet predicts that BMP9/10 from stellate cells would signal through ALK1 to induce KC development and maintenance, a prediction in line with our previous NicheNet analysis (Bonnardel et al., 2019), another recent study has suggested that transforming growth factor (TGF)β signaling would be important for KCs (Sakai et al., 2019). This prediction was made on the basis of SMAD4 signaling, which is a common downstream effector of both TGF-β Receptor and ALK1-induced signaling. As TGF-β was also found to participate in an ALK1-TGF-β receptor containing signaling complex (Goumans et al., 2003), we thus examined whether TGF-β signaling is important for KCs. To this end we utilized an ALK1-Fc trap or TGF-β type II receptor (TGFβRII)-Fc trap alongside appropriate isotype controls. ALK1-Fc selectively sequesters BMP9/10 (Desroches-Castan et al., 2021), while TGFβRII-Fc selectively interferes with TGF-β1/TGF-β3 receptor binding (Komesli et al., 2017). *Clec4f-Dtr* mice were depleted of KCs and simultaneously treated with either receptor-Fc traps or isotype controls and KC development was examined 7 days later (Figure 7I). In line with the chimera study, treatment with ALK1-Fc significantly abrogated KC development; however, blocking TGFβ signaling had only a minor effect on the proportion of VSIG4^−^ macs (Figure 7J). Altogether this validates the NicheNet prediction and demonstrates that the evolutionarily conserved ALK1-BMP9/10 axis is crucial for the development and maintenance of KCs.

## Discussion

To generate a practical cellular atlas of any human tissue and unravel the cell-cell circuits essential for the identities of cells inhabiting that tissue, four key pieces of information are required: (1) an inventory of all cells present, (2) the location of the different cells within the tissue to identify interactions between neighboring cells, (3) an alignment between the human and animal models allowing for any predicted cell-cell interactions to be perturbed, and (4) the identification of reliable antibody-based panels for the efficient screening of different patients and/or transgenic animals. Here, by integrating single-cell and spatial transcriptomic and proteomic data, we provide these 4 pieces of information for the liver and uncover evolutionarily conserved microenvironmental circuits controlling the development of hepatic macs.

Unraveling the spatial localization of all hepatic cells, we identify LAMs around the bile ducts in the healthy mouse, human, and macaque liver. However, when steatosis is present, LAMs are preferentially recruited to the steatotic regions of the liver. This spatial information at least partially invalidates the hypothesis that LAM identity is specifically induced by fibrotic SCs (Ramachandran et al., 2019). Rather, our data suggest that LAMs are induced by local lipid exposure. We also provide an alignment of the liver atlas across seven species. This reveals the conserved and unique transcriptomic programs of steady-state KCs and uncovers the spatially restricted and conserved ligand-receptor pairs between KCs and the cells constituting their niche. Underlining the need to first characterize the healthy tissue before attempting to understand how disease perturbs the cells, we identify the DLL-NOTCH interaction to be an evolutionarily conserved cross-talk between homeostatic LSECs and KCs and therefore not unique to hepatocellular carcinoma or fibrosis, as proposed (Ramachandran et al., 2019; Sharma et al., 2020). Similarly, we find that *FOLR2* expression is not specific to tumor-associated hepatic macs (Sharma et al., 2020) but is expressed by KCs in the healthy mouse and human liver. Finally, we apply a proteogenomic pipeline starting from broad oligo-conjugated antibody panels for both single-cell and spatial profiling. This is crucial as transcriptomic profiling does not always correspond with the ability to detect proteins by flow cytometry or microscopy. By screening broadly, we identify the best surface markers for the isolation and localization of hepatic macs and their respective niche cells. This allows both the validation of the spatial location at the single-cell level, and the efficient screening of transgenic mouse models for the loss of KCs. Characterization of *Fcgr1*-Crex*Acvrl1*^fl/fl^ mice using our defined panel readily demonstrates the cruciality of the ALK1-BMP9/10 axis in KC development emphasizing that mac-stromal-cell cross-talk goes much further than the exchange of growth factors (Guilliams et al., 2020; Zhou et al., 2018). Moving forward, applying these relatively cheap antibody panels to large patient cohorts or multiple transgenic mouse models should enable any perturbations disturbing liver homeostasis to be efficiently identified.

### Limitations of the study

The current study has two main limitations. First, the analysis of the human liver remains restricted by the number of human patients included (19 patients for the sc/snRNA-seq, 4 patients for Visium, and 15 patients by microscopy). While this study provides markers to cheaply and efficiently screen large patient cohorts allowing this analysis to be extended, given the heterogeneity between patients, multiple studies will need to be integrated in a single analysis if we are to be able to interrogate transcriptomic differences in a particular cell subset related to age, sex, ethnicity, or pathological parameters. Second, this study highlights that more research will be needed to fully characterize human SCs. As we could only retrieve these cells through snRNA-seq this means we have not been able to identify good surface markers through CITE-seq. Additionally, for small populations of fibroblasts we did not recover enough nuclei to truly probe their heterogeneity. Better isolation protocols are required to retrieve and enrich these cells from the human liver in order to run a broad panel of CITE-seq markers to identify the different subsets and their corresponding surface markers.

## STAR★Methods

### Key resources table

**Table:** 

REAGENT or RESOURCE	SOURCE	IDENTIFIER
**Antibodies:Flowcytometry/confocalmicroscopy**		

Rat Monoclonal CD102-FITC (3C4)	Biolegend	105606; RRID: AB_313199
Rat Monoclonal CD103-BV750 (M290)	BD Biosciences	747478; RRID: AB_2872154
Rat Monoclonal CD11b-BUV395 (M1/70)	BD Biosciences	565976; RRID: AB_2721166
Rat Monoclonal CD11b-BV605 (M1/70)	BD Biosciences	563015; RRID: AB_2737951
Rat Monoclonal MHCII-BUV805 (M5/114,15,2)	BD Biosciences	748844; RRID: AB_2873247
Rat Monoclonal MHCII-FITC (M5/114,15,2)	Thermo Fisher Scientific	11-5321-85; RRID: AB_465233
Rat Monoclonal CD14-PE Dazzle 594 (Sa14-2)	Biolegend	123325; RRID: AB_2721697
Rat Monoclonal CD43-BUV737 (S7)	BD Biosciences	612840; RRID: AB_2870162
Rat Monoclonal Ly6C-eFluor450 (HK1.4)	eBioscience	48-5932-82; RRID: AB_10805519
Armenian Hamster Monoclonal CD11c-PE-Cy7 (N418)	eBioscience	25-0114-82; RRID: AB_469590
Armenian Hamster Monoclonal CD11c-Unconjugated (N418)	Biolegend	117302; RRID: AB_313771
Rat Monoclonal CD86-BV605 (GL-1)	Biolegend	105037; RRID: AB_11204429
Rat Monoclonal CD54-PE (YN1/1.7.4)	Biolegend	116107; RRID: AB_313698
Rat Monoclonal CD54-AF488 (YN1/1,7,4)	Biolegend	116111; RRID: AB_493494
Rat Monoclonal CD172a-BB630P (P84)	BD Customs	624294
Mouse Monoclonal XCR1 - BV650 (ZET)	BioLegend	148220; RRID: AB_2566410
Rat Monoclonal Tim4-PerCP-Cy5.5 (RMT4-54)	eBioscience	46-5866-82; RRID: AB_2573781
Rat Monoclonal VSIG4-PECy7 (NLA14)	eBioscience	25-5752-82; RRID: AB_2637431
Rat Monoclonal CD19-PECy5 (1D3)	eBioscience	15-0193-82; RRID: AB_657672
Rat Monoclonal CD19-Unconjugated (1D3)	BD Biosciences	553783; RRID: AB_395047
Rat Monoclonal CD26-FITC (H194-112)	BD Biosciences	559652; RRID: AB_398295
Rat Monoclonal CD38-AF700 (90)	eBioscience	56-0381-82; RRID: AB_657740
Armenian Hamster Monoclonal CD3e-PECy5 (145-2C11)	TONBO Biosciences	55-0031; RRID: AB_2621815
Rat Monoclonal CD3-Unconjugated (17A2)	Bioceros	N/A
Rat Monoclonal CD45-BV510 (30-F11)	BioLegend	103138; RRID: AB_2563061
Mouse Monoclonal CD45.1-PE (A20)	BD Biosciences	553776; RRID: AB_395044
Mouse Monoclonal CD45.1-Unconjugated (A20)	Biolegend	110702; RRID: AB_313491
Mouse Monoclonal CD45.2-AF700 (104)	eBioscience	56-0454-82; RRID: AB_657752
Rat Monoclonal CD45R-PE-Cy5 (RA3-6B2)	BD Biosciences	553091; RRID: AB_394621
Rat Monoclonal Ly6G-BUV563 (1A8)	BD Biosciences	612921; RRID: AB_2870206
Mouse Monoclonal CD64-BV711 (X54-5/7.1)	BioLegend	139311; RRID: AB_2563846
Mouse Monoclonal NK1.1-PECy5 (PK136)	BioLegend	108716; RRID: AB_493590
Rat Monoclonal Ter119-PECy5 (TER-119)	eBioscience	15-5921-82; RRID: AB_468810
Rat Monoclonal F4/80-BV785 (BM8)	BioLegend	123141; RRID: AB_2563667
Rat Monoclonal F4/80-Unconjugated (CI:A3-1)	Bio-Rad	MCA497R; RRID: AB_323279
Rat Monoclonal F4/80-AF594 (BM8)	Biolegend	123140; RRID: AB_2563241
Rat Monoclonal CLEC2-PE (17D9)	Biolegend	146104; RRID: AB_2562382
Rat Monoclonal FOLRB-PE (10/FR2)	Biolegend	153304; RRID: AB_2721344
Rat Monoclonal FOLRB-Unconjugated (10/FR2)	Biolegend	153302; RRID: AB_2687271
Rat Monoclonal SiglecF-BUV395 (E50-2440)	BD Biosciences	740280; RRID: AB_2740019
Rat Monoclonal CD207-AF647	Imgenex	DDX0362A647; RRID: AB_1148741
Rat Monoclonal CD206-AF647	Biolegend	141712; RRID: AB_10900420
Rat Monoclonal CD326 (EPCAM)-APC (G8.8)	eBioscience	17-5791-82; RRID: AB_2716944
Goat Polyclonal Clec4F-Unconjugated	R & D Systems	AF2784; RRID: AB_2081339
Rat Monoclonal Clec4F-Unconjugated (370901)	R & D Systems	MAB2784; RRID: AB_2081338
Goat Polyclonal VSIG4-Unconjugated	R & D Systems	AF4646; RRID: AB_2257239
Goat Polyclonal Lyve1-Unconjugated	R&D Systems	AF2125; RRID: AB_2297188
Donkey Anti-Goat IgG-AF488	Thermo Fisher Scientific	A-11055; RRID: AB_2534102
Donkey Anti-Goat IgG–AF633	Thermo Fisher Scientific	A-21082; RRID: AB_2535739
Donkey Anti-Goat IgG-AF647	Thermo Fisher Scientific	A-21447; RRID: AB_2535864
Donkey Anti-Rat IgG-Cy3	Jackson ImmunoResearch	712-166-153; RRID: AB_2340669
Donkey Anti-Mouse IgG–AF555	Thermo Fisher Scientific	A-31570; RRID: AB_2536180
Donkey Anti-Rabbit IgG-AF647	Thermo Fisher Scientific	A-31573; RRID: AB_2536183
Donkey Anti-Rabbit IgG-AF680	Thermo Fisher Scientific	A-32802; RRID: AB_2762836
Goat Anti-Rabbit IgG–AF514	Thermo Fisher Scientific	A-31558; RRID: AB_2536173
Goat Anti-Hamster IgG-Cy3	Jackson ImmunoResearch	127-165-160; RRID: AB_2338989
Goat Anti-Hamster IgG-AF594	Jackson ImmunoResearch	127-585-160; RRID: AB_2338999
Goat Anti-Rat IgG-AF568	Thermo Fisher Scientific	A-11077; RRID: AB_2534121
Goat Anti-Chicken IgY-AF680	Abcam	Ab175779;
Rat Monoclonal CD31-Unconjugated (MEC13.3)	BD Biosciences	550274; RRID: AB_393571
Rabbit Polyclonal Desmin-Unconjugated	Abcam	ab15200; RRID: AB_301744
Rabbit Polyclonal Glutamine Synthetase - Unconjugated	Abcam	ab73593; RRID: AB-2247588
Goat Anti-Rabbit IgG-AF514	Thermo Fisher Scientific	A-31558; RRID: AB_2536173
Rabbit Monoclonal GPNMB- unconjugated (EPR18226-147)	Abcam	ab188222;
Polyclonal Chicken anti-GFP	Aves Labs	GFP-1010; RRID: AB_2307313
Mouse Monoclonal FOLR2-PE (94b/FOLR2)	Biolegend	391704; RRID: AB_2721336
Mouse Monoclonal CD45-APCCy7 (2D1)	Biolegend	368516; RRID: AB_2566376
Mouse Monoclonal CD14-PETexasRed (TuK4)	Thermo Fisher Scientific	MHCD1417; RRID:AB_10373552
Mouse Monoclonal CD1C-Unconjugated (L161)	Biolegend	331502; RRID:AB_1088995
Mouse Monoclonal CD163-Unconjugated (10D6)	Thermo Fisher Scientific	MA5-11458; RRID: AB_10982556
Mouse Monoclonal EPCAM-eF660 (1B7)	eBioscience	50-9326-42: RRID: AB_10598658
Rabbit Monoclonal CD68-Unconjugated (EPR20545)	Abcam	Ab213363; RRID: AB_2801637
Rabbit Monoclonal CD34-PE (EP373Y)	Abcam	Ab223930;
Anti- Mouse CD38 (REA616) FITC	Miltenyi Biotec	130-122-955; RRID: AB_2811415
Anti-Mouse CD26 (REA1196) PE	Miltenyi Biotec	130-122-775; RRID: AB_2801934
Anti-Mouse F4/80 (REA126) FITC	Miltenyi Biotec	130-117-509; RRID: AB_2727970
Anti-Mouse CD5 (53-7.3) FITC	Miltenyi Biotec	130-102-574; RRID: AB_2658608
Anti-Mouse CD79B (REA1117) PE	Miltenyi Biotec	130-119-425; RRID: AB_2751702
Anti-Mouse CD45R/B220 (RA3-6B2) FITC	Miltenyi Biotec	130-118-323; RRID: AB_2751482
Anti-Mouse CD43 (L11) PE	Miltenyi Biotec	130-102-594; RRID: AB_2661309
Anti-Mouse CCR2 (REA538) PE	Miltenyi Biotec	130-117-548; RRID: AB_2727981
Anti-Mouse MHCII (M5/114.15.2) FITC	Miltenyi Biotec	130-123-666; RRID: AB_2802055
Anti-Mouse CD73 (REA778) PE	Miltenyi Biotec	130-111-331; RRID: AB_2659153
Anti-Mouse CD146 (REA1064) FITC	Miltenyi Biotec	130-118-252; RRID: AB_2751472
Anti-Mouse CD90.2 (30-H12) PE	Miltenyi Biotec	130-120-091; RRID: AB_2751997
Anti-Mouse CD138 ( REA104) PE	Miltenyi Biotec	130-120-810; RRID: AB_2752204
Anti-Mouse CD29 (REA1074) PE	Miltenyi Biotec	130-119-165; RRID: AB_2751649
Anti-Mouse CD105 (REA1058) FITC	Miltenyi Biotec	130-118-173; RRID: AB_2733613
Anti-Human CD36 (REA760) PE	Miltenyi Biotec	130-110-877; RRID: AB_2657728
Anti-Human CD206 (REAL518) APC	Miltenyi Biotec	130-122-168; RRID: AB_2857557
Anti-Human Collagen IV (REAL567) PE	Miltenyi Biotec	130-122-866; RRID: AB_2857566
Anti-Human CD105 (REA794) PE	Miltenyi Biotec	130-112-163; RRID: AB_2654424
Anti-Human CD146 (REA773) APC	Miltenyi Biotec	130-111-323; RRID: AB_2655179
Anti-Human CD90 (REA897) PE	Miltenyi Biotec	130-114-860; RRID: AB_2726811
Anti-Human DESMIN (REA1134) PE	Miltenyi Biotec	130-119-490; RRID: AB_2857461
Anti-Human CD74 (REA1103) PE	Miltenyi Biotec	130-119-203; RRID: AB_2733836
Anti-Human EPCAM (REA764) FITC	Miltenyi Biotec	130-110-998; RRID: AB_2657493
Anti-Human CD68 (REAL566) PE	Miltenyi Biotec	130-123-368; RRID: AB_2857592
Anti-Human CD1c (REA694) APC	Miltenyi Biotec	130-110-537; RRID: AB_2656040
Anti-Human CD3 (REA1151) APC	Miltenyi Biotec	130-120-269; RRID: AB_2876933
Anti-Human CD5 (REA782) PE	Miltenyi Biotec	130-110-990; RRID: AB_2658593
Anti-Human CD7 (REA1244) PE	Miltenyi Biotec	130-124-939; RRID: AB_2819716
Anti-Human CD19 (REAL106) PE	Miltenyi Biotec	130-122-649; RRID: AB_2784034
Anti-Human CD22 (REA340) APC	Miltenyi Biotec	130-120-762; RRID: AB_2752186
Anti-Human S100A8 (REA917) PE	Miltenyi Biotec	130-115-253; RRID: AB_2726963
Anti-Human CD177 (REA258) PE	Miltenyi Biotec	130-101-525; RRID: AB_2655641
Anti-Human CD169 (REA1176) PE	Miltenyi Biotec	130-121-115; RRID: AB_2783985
Anti-Human CD163 (REA406) PE	Miltenyi Biotec	130-121-316; RRID: AB_2857545

**Antibodies:CITE-seq**		

TotalSeq-A0001 anti-mouse CD4 (RM4-5)	BioLegend	100569; RRID: AB_2749956
TotalSeq-A0002 anti-mouse CD8a (53-6.7)	BioLegend	100773; RRID: AB_2734151
TotalSeq-A0003 anti-mouse CD366 (Tim-3) (RMT3-23)	BioLegend	119729; RRID: AB_2734178
TotalSeq-A0004 anti-mouse CD279 (PD-1) (RMP1-30)	BioLegend	109123; RRID: AB_2734169
TotalSeq-A0005 anti-human CD80 (2D10)	BioLegend	305239; RRID: AB_2749958
TotalSeq-A0006 anti-human CD86 (IT2.2)	BioLegend	305443; RRID: AB_2734273
TotalSeq-A0007 anti-human CD274 (29E.2A3)	BioLegend	329743; RRID: AB_2749959
TotalSeq-A0008 anti-human CD273 (24F.10C112)	BioLegend	329619; RRID: AB_2734321
TotalSeq-A0009 anti-human CD275 (2D3)	BioLegend	309413; RRID: AB_2734278
TotalSeq-A0010 anti-human CD276 (DCN.70)	BioLegend	331607; RRID: AB_2734327
TotalSeq-A0012 anti-mouse CD117 (c-kit) (2B8)	BioLegend	105843; RRID: AB_2749960
TotalSeq-A0013 anti-mouse Ly-6C (HK1.4)	BioLegend	128047; RRID: AB_2749961
TotalSeq-A0014 anti-mouse/human CD11b (M1/70)	BioLegend	101265; RRID: AB_2734152
TotalSeq-A0015 anti-mouse Ly-6G (1A8)	BioLegend	127655; RRID: AB_2749962
TotalSeq-A0016 anti-human Galectin9 (9M1-3)	BioLegend	Barcode: ACTCACTGGAGTCTC
TotalSeq-A0020 anti-human CD270 (122)	BioLegend	318813; RRID: AB_2734293
TotalSeq-A0021 anti-human CD252 (11C3.1)	BioLegend	Barcode: TTTAGTGATCCGACT
TotalSeq-A0022 anti-human CD137L (5F4)	BioLegend	311509; RRID: AB_2734284
TotalSeq-A0023 anti-human CD155 (SKII.4)	BioLegend	337623; RRID: AB_2749963
TotalSeq-A0024 anti-human CD112 (TX31)	BioLegend	337417; RRID: AB_2749964
TotalSeq-A0026 anti-human CD47 (CC2C6)	BioLegend	323129; RRID: AB_2734305
TotalSeq-A0027 anti-human CD70 (113-16)	BioLegend	355117; RRID: AB_2749965
TotalSeq-A0028 anti-human CD30 (BY88)	BioLegend	333913; RRID: AB_2749966
TotalSeq-A0029 anti-human CD48 (BJ40)	BioLegend	336709; RRID: AB_2734342
TotalSeq-A0031 anti-human CD40 (5C3)	BioLegend	334346; RRID: AB_2749968
TotalSeq-A0032 anti-human CD154 (24-31)	BioLegend	310843; RRID: AB_2734283
TotalSeq-A0033 anti-human CD52 (HI186)	BioLegend	316017; RRID: AB_2734292
TotalSeq-A0034 anti-human CD3 (UCHT1)	BioLegend	300475; RRID: AB_2734246
TotalSeq-A0047 anti-human CD56 (5.1H11)	BioLegend	362557; RRID: AB_2749970
TotalSeq-A0050 anti-human CD19 (HIB19)	BioLegend	302259; RRID: AB_2734256
TotalSeq-A0052 anti-human CD33 (P67.6)	BioLegend	366629; RRID: AB_2734409
TotalSeq-A0053 anti-human CD11c (S-HCL-3)	BioLegend	371519; RRID: AB_2749971
TotalSeq-A0054 anti-human CD34 (581)	BioLegend	343537; RRID: AB_2749972
TotalSeq-A0056 anti-human CD269 (19F2)	BioLegend	357521; RRID: AB_2749974
TotalSeq-A0057 anti-human B2M (2M2)	BioLegend	316321; RRID: AB_2749975
TotalSeq-A0058 anti-human HLA-ABC (W6/32)	BioLegend	311445; RRID: AB_2749976
TotalSeq-A0060 anti-human CD90 (5E+10)	BioLegend	328135; RRID: AB_2734312
TotalSeq-A0061 anti-human CD117 (104D2)	BioLegend	313241; RRID: AB_2734287
TotalSeq-A0062 anti-human CD10 (HI10a)	BioLegend	312231; RRID: AB_2832627
TotalSeq-A0063 anti-human CD45RA (HI100)	BioLegend	304157; RRID: AB_2734267
TotalSeq-A0064 anti-human CD123 (6H6)	BioLegend	306037; RRID: AB_2749977
TotalSeq-A0066 anti-human CD7 (CD7-6B7)	BioLegend	343123; RRID: AB_2734345
TotalSeq-A0068 anti-human CD105 (43A3)	BioLegend	323221; RRID: AB_2750350
TotalSeq-A0069 anti-human CD201 (RCR-401)	BioLegend	351907; RRID: AB_2749978
TotalSeq-A0070 anti-human/mouse CD49f (GoH3)	BioLegend	313633; RRID; AB_2734291
TotalSeq-A0073 anti-mouse/human CD44 (IM7)	BioLegend	103045; RRID: AB_2734154
TotalSeq-A0074 anti-mouse CD54 (YN1/1.7.4)	BioLegend	116127; RRID: AB_2734177
TotalSeq-A0074 anti-mouse CD90.2 (30-H12)	Biolegend	105345; RRID: AB_2734166
TotalSeq-A0076 anti-mouse/human CD15 (SSEA-1) (MC-480)	BioLegend	125615; RRID: AB_2800603
TotalSeq-A0077 anti-mouse CD73 (TY/11.8)	BioLegend	127227; RRID: AB_2749980
TotalSeq-A0078 anti-mouse CD49d (R1-2)	BioLegend	103623; RRID: AB_2734159
TotalSeq-A0079 anti-mouse CD200 (OX2) (OX-90)	BioLegend	123811; RRID: AB_2734191
TotalSeq-A0080 anti-human CD8a (RPA-T8)	BioLegend	301067; RRID: AB_2734248
TotalSeq-A0081 anti-human CD14 (M5E2)	BioLegend	301855: RRID: AB_2734254
TotalSeq-A0083 anti-human CD16 (3G8)	BioLegend	302061; RRID: AB_2734255
TotalSeq-A0085 anti-human CD25 (BC96)	BioLegend	302643; RRID: AB_2734258
TotalSeq-A0087 anti-human CD45RO (UCHL1)	BioLegend	304255; RRID: AB_2734268
TotalSeq-A0088 anti-human CD279 (EH12.2H7)	BioLegend	329955; RRID: AB_2734322
TotalSeq-A0089 anti-human TIGIT (A15153G)	BioLegend	372725; RRID: AB_2734426
TotalSeq-A0090 Mouse IgG1, κ isotype Ctrl (MOPC-21)	BioLegend	400199; RRID: AB_2868412
TotalSeq-A0091 Mouse IgG2a, κ isotype Ctrl (MOPC-173)	BioLegend	400285;
TotalSeq-A0092 Mouse IgG2b, κ isotype Ctrl (MPC-11)	BioLegend	400373;
TotalSeq-A0093 anti-mouse CD19 (B4)	BioLegend	115559; RRID: AB_2749981
TotalSeq-A0094 anti-mouse CD3e (145-2C11)	BioLegend	Barcode: TAATGCCAGTTGTGC
TotalSeq-A0095 Rat IgG2b, κ Isotype Ctrl (RTK4530)	BioLegend	400673;
TotalSeq-A0097 anti-mouse CD25 (PC61)	BioLegend	102055; RRID: AB_2749982
TotalSeq-A0098 anti-mouse CD135 (A2F10)	BioLegend	135316; RRID: AB_2749983
TotalSeq-A0100 anti-human CD20 (2H7)	BioLegend	302359; RRID: AB_2749984
TotalSeq-A0101 anti-human CD335 (900)	BioLegend	331943; RRID: AB_2800875
TotalSeq-A0102 anti-human CD294 (BM16)	BioLegend	350127; RRID: AB_2734360
TotalSeq-A0103 anti-mouse/human CD45R/B220	BioLegend	103263; RRID: AB_2734158
TotalSeq-A0104 anti-mouse CD102 (3C4 (MIC2/4))	BioLegend	105613; RRID: AB_2734167
TotalSeq-A0105 anti-mouse CD115 (CSF-1R) (AFS98)	BioLegend	135533; RRID: AB_2734198
TotalSeq-A0106 anti-mouse CD11c (N418)	BioLegend	117355; RRID: AB_2750352
TotalSeq-A0107 anti-mouse CD21/CD35 (CR2/CR1) (7E9)	BioLegend	123427; RRID: AB_2750540
TotalSeq-A0108 anti-mouse CD23 (B3B4)	BioLegend	101635; RRID: AB_2750358
TotalSeq-A0109 anti-mouse CD16/32 (93)	BioLegend	101343; RRID: AB_2750532
TotalSeq-A0110 anti-mouse CD43 (S11)	BioLegend	143211; RRID: AB_2750541
TotalSeq-A0111 anti-mouse CD5 (53-7.3)	BioLegend	100637; RRID: AB_2749985
TotalSeq-A0112 anti-mouse CD62L (MEL-14)	BioLegend	104451; RRID: AB_2750364
TotalSeq-A0113 anti-mouse CD93 (AA4.1, early B lineage) (AA4.1)	BioLegend	136513; RRID: AB_2750375
TotalSeq-A0114 anti-mouse F4/80 (BM8)	BioLegend	123153; RRID: AB_2749986
TotalSeq-A0115 anti-mouse FcεRIα (MAR-1)	BioLegend	134333; RRID: AB_2749987
TotalSeq-A0117 anti-mouse I-A/I-E (M5/114.15.2)	BioLegend	107653; RRID: AB_2750505
TotalSeq-A0118 anti-mouse NK-1.1 (PK136)	BioLegend	108755; RRID: AB_2750536
TotalSeq-A0119 anti-mouse Siglec H (551)	BioLegend	129615; RRID: AB_2750537
TotalSeq-A0120 anti-mouse TCRb (H57-597)	Biolegend	109247; RRID: AB_2750538
TotalSeq-A0121 anti-mouse TCRgd (GL3)	Biolegend	118137; RRID: AB_2749988
TotalSeq-A0122 anti-mouse Ter-119 (Ter-119)	Biolegend	116247; RRID: AB_2749989
TotalSeq-A0123 anti-human CD326 (9C4)	BioLegend	324241; RRID: AB_2750362
TotalSeq-A0124 anti-human CD31 (WM59)	BioLegend	303137: RRID: AB_2750360
TotalSeq-A0126 anti-human CD133 (clone 7)	BioLegend	372815; RRID: AB_2750353
TotalSeq-A0127 anti-human Podoplanin (NC-08)	BioLegend	337019; RRID; AB_2749990
TotalSeq-A0128 anti-human CD140a (16A1)	BioLegend	323509; RRID: AB_2750354
TotalSeq-A0129 anti-human CD140b (18A2)	BioLegend	323609; RRID: AB_2783192
TotalSeq-A0130 anti-mouse Ly-6A/E (Sca-1) (D7)	BioLegend	108147; RRID: AB_2750535
TotalSeq-A0131 anti-human Cadherin (16G5)	BioLegend	368715; RRID: AB_2749991
TotalSeq-A0132 anti-human EGFR (AY13)	BioLegend	352923; RRID: AB_2734373
TotalSeq-A0134 anti-human CD146 (P1H12)	BioLegend	361017: RRID: AB_2750355
TotalSeq-A0136 anti-human IgM (MHM-88)	BioLegend	314541; RRID: AB_2749992
TotalSeq-A0138 anti-human CD5 (UCHT2)	BioLegend	300635; RRID: AB_2750346
TotalSeq-A0139 anti-human TCR (B1)	BioLegend	331229; RRID: AB_2734325
TotalSeq-A0140 anti-human CD183 (G025H7)	BioLegend	353745; RRID: AB_2749993
TotalSeq-A0141 anti-human CD195 (J418F1)	BioLegend	359135; RRID: AB_2749994
TotalSeq-A0142 anti-human CD32 (FUN-2)	BioLegend	303223; RRID: AB_2749995
TotalSeq-A0143 anti-human CD196 (G034E3)	BioLegend	353437; RRID: AB_2750534
TotalSeq-A0144 anti-human CD185 (J252D4)	BioLegend	356937; RRID: AB_2750356
TotalSeq-A0145 anti-human CD103 (Ber-ACT8)	BioLegend	350231; RRID: AB_2749996
TotalSeq-A0146 anti-human CD69 (FN50)	BioLegend	310947; RRID: AB_2749997
TotalSeq-A0147 anti-human CD62L (DREG-56)	BioLegend	304847; RRID: AB_2750365
TotalSeq-A0148 anti-human CD197 (G043H7)	BioLegend	353247; RRID: AB_2750357
TotalSeq-A0149 anti-human CD161 (HP-3G10)	BioLegend	339945; RRID: AB_2749998
TotalSeq-A0151 anti-human CD152 (BNI3)	BioLegend	369619; RRID: AB_2734423
TotalSeq-A0152 anti-human CD223 (11C3C65)	BioLegend	369333; RRID: AB_2749999
TotalSeq-A0153 anti-human KLRG1 (SA231A2)	BioLegend	367721; RRID: AB_2750373
TotalSeq-A0155 anti-human CD107a (H4A3)	BioLegend	328647; RRID: AB_2750351
TotalSeq-A0156 anti-human CD95 (DX2)	BioLegend	305649; RRID: AB_2750368
TotalSeq-A0158 anti-human CD134 (Ber-ACT35)	BioLegend	350033; RRID: AB_2783245
TotalSeq-A0159 anti-human HLA-DR (L243)	BioLegend	307659; RRID: AB_2750001
TotalSeq-A0160 anti-human CD1c (L161)	BioLegend	331539; RRID: AB_2734326
TotalSeq-A0162 anti-human CD64 (10.1)	BioLegend	305037; RRID: AB_2750366
TotalSeq-A0163 anti-human CD141 (M80)	BioLegend	344121; RRID: AB_2783229
TotalSeq-A0164 anti-human CD1d (51.1)	BioLegend	350317; RRID: AB_2750370
TotalSeq-A0165 anti-human CD314 (1D11)	BioLegend	320835; RRID: AB_2734298
TotalSeq-A0166 anti-human CD66b (6/40c)	BioLegend	392905; RRID: AB_2750372
TotalSeq-A0167 anti-human CD35 (E11)	BioLegend	333407; RRID: AB_2783217
TotalSeq-A0168 anti-human CD57 (QA17A04)	BioLegend	393319; RRID: AB_2810588
TotalSeq-A0169 anti-human CD366 (F38-2E2)	BioLegend	345047; RRID: AB_2800924
TotalSeq-A0170 anti-human CD272 (MIH26)	BioLegend	344525; RRID: AB_2750002
TotalSeq-A0171 anti-human/mouse/rat CD278 (ICOS) (C398.4A)	BioLegend	313555; RRID: AB_2800824
TotalSeq-A0173 anti-mouse CD206 (MMR) (C068C2)	BioLegend	Barcode: TCAACTCGGTGTTGC
TotalSeq-A0174 anti-human CD58 (TS2/9)	BioLegend	330919; RRID: AB_2750003
TotalSeq-A0175 anti-human CD96 (NK92.39)	BioLegend	338419; RRID: AB_2750004
TotalSeq-A0176 anti-human CD39 (A1)	BioLegend	328233; RRID: AB_2750005
TotalSeq-A0177 anti-human CD178 (NOK-1)	BioLegend	306413; RRID: AB_2750500
TotalSeq-A0180 anti-human CD24 (ML5)	BioLegend	311137: RRID: AB_2750374
TotalSeq-A0181 anti-human CD21 (Bu32)	BioLegend	354915; RRID: AB_2750006
TotalSeq-A0182 anti-mouse CD3 (17A2)	Biolegend	100251; RRID: AB_2750533
TotalSeq-A0184 anti-mouse CD335 (NKp46) (29A1.4)	BioLegend	137633; RRID: AB_2734199
TotalSeq-A0185 anti-human CD11a (TS2/4)	BioLegend	350615; RRID: AB_2734365
TotalSeq-A0186 anti-human IgA (HP6123)	BioLegend	Barcode: AAGATGTCCGAGCAA
TotalSeq-A0187 anti-human CD79b (CB3-1)	BioLegend	341415; RRID: AB_2750347
TotalSeq-A0188 anti-human CD66a_c_e (ASL-32)	BioLegend	342319; RRID: AB_2783223
TotalSeq-A0189 anti-human CD244 (C1.7)	BioLegend	329527; RRID: AB_2750007
TotalSeq-A0190 anti-mouse CD274 (B7-H1, PD-L1) (MIH6)	BioLegend	153604; RRID: AB_2783125
TotalSeq-A0191 anti-mouse/rat/human CD27 (LG.3A10 )	BioLegend	124235; RRID: AB_2750344
TotalSeq-A0192 anti-mouse CD20 (SA275A11)	BioLegend	150423; RRID: AB_2734214
TotalSeq-A0193 anti-mouse CD357 (GITR) (DTA-1)	BioLegend	126319; RRID: AB_2734195
TotalSeq-A0194 anti-mouse CD137 (17B5)	BioLegend	106111; RRID: AB_2783048
TotalSeq-A0195 anti-mouse CD134 (OX-40) (OX-86)	BioLegend	119426; RRID: AB_2750376
TotalSeq-A0196 anti-human CD235ab (HIR2)	BioLegend	306623; RRID: AB_2750008
TotalSeq-A0197 anti-mouse CD69 (H1.2F3)	BioLegend	104546; RRID: AB_2750539
TotalSeq-A0198 anti-mouse CD127 (IL-7Rα) (A7R34)	BioLegend	135045; RRID: AB_2750009
TotalSeq-A0200 anti-mouse CD86 (GL-1)	BioLegend	105047; RRID: AB_2750348
TotalSeq-A0201 anti-mouse CD103 (2E7)	BioLegend	121437; RRID: AB_2750349
TotalSeq-A0202 anti-mouse CD64 (FcγRI) (X54-5/7.1)	BioLegend	139325; RRID: AB_2750367
TotalSeq-A0203 anti-mouse CD150 (SLAM) (TC15-12F12.2)	BioLegend	115945; RRID: AB_2783055
TotalSeq-A0204 anti-mouse CD28 (37.51)	BioLegend	Barcode: ATTAAGAGCGTGTTG
TotalSeq-A0205 anti-human CD206 (15-2)	BioLegend	321143; RRID: AB_2750010
TotalSeq-A0206 anti-human CD169 (7-239)	BioLegend	346011; RRID: AB_2750011
TotalSeq-A0207 anti-human CD370 (8F9)	BioLegend	353807; RRID: AB_2814293
TotalSeq-A0208 anti-human XCR1 (S15046E)	BioLegend	372613; RRID: AB_2783286
TotalSeq-A0209 anti-mouse TCR (2.11)	BioLegend	141113; RRID: AB_2800654
TotalSeq-A0210 anti-mouse TCR (536)	BioLegend	137507; RRID: AB_2810404
TotalSeq-A0211 anti-mouse TCR (UC3-10A6)	BioLegend	137709; RRID: AB_2783100
TotalSeq-A0212 anti-mouse CD24 (M1/69)	BioLegend	101841; RRID: AB_2750380
TotalSeq-A0213 anti-human Notch (MHN1-519)	BioLegend	352109; RRID: AB_2783247
TotalSeq-A0214 anti-human/mouse integrin β7 (FIB504)	BioLegend	321227; RRID: AB_2750504
TotalSeq-A0215 anti-human CD268 (11C1)	BioLegend	316925; RRID: AB_2750502
TotalSeq-A0216 anti-human CD42b (HIP1)	BioLegend	303937; RRID: AB_2783163
TotalSeq-A0217 anti-human CD54 (HA58)	BioLegend	353123; RRID: AB_2750384
TotalSeq-A0218 anti-human CD62P (AK4)	BioLegend	304933; RRID: AB_2750386
TotalSeq-A0219 anti-human CD119 (GIR-208)	BioLegend	308607; RRID: AB_2750385
TotalSeq-A0221 anti-human IRF5 (11F4A09)	BioLegend	Barcode: GGTAGCCCTAGAGTA
TotalSeq-A0224 anti-human TCR (IP26)	BioLegend	306737: RRID: AB_2783167
TotalSeq-A0225 anti-mouse CD196 (CCR6) (29-2L17)	BioLegend	129825; RRID: AB_2783083
TotalSeq-A0226 anti-mouse CD106 (429 (MVCAM.A))	BioLegend	105725; RRID: AB_2783044
TotalSeq-A0227 anti-mouse CD122 (IL-2Rb) (5H4)	BioLegend	Barcode: GGTATGCGACACTTA
TotalSeq-A0228 anti-mouse CD183 (CXCR3) (CXCR3-173)	BioLegend	Barcode: GTTCACGCCGTAGACT
TotalSeq-A0229 anti-mouse CD62P (P-selectin) (RMP-1)	BioLegend	Barcode: TGTGTGCCGTAGACT
TotalSeq-A0230 anti-mouse CD8b (Ly-3) (YTS156.7.7)	BioLegend	126623; RRID: AB_2800615
TotalSeq-A0232 anti-mouse MAdCAM-1 (MECA-367)	BioLegend	120713; RRID: AB_2783058
TotalSeq-A0235 anti-mouse TCR (KJ16-133.18)	BioLegend	118415; RRID: AB_2783056
TotalSeq-A0236 Rat IgG1, κ Isotype Ctrl (RTK2071)	BioLegend	400459;
TotalSeq-A0237 Rat IgG1, λ Isotype Ctrl (G0114F7)	BioLegend	401919;
TotalSeq-A0238 Rat IgG2a, κ Isotype Ctrl (RTK2758)	BioLegend	400571;
TotalSeq-A0240 Purified Rat IgG2c, κ Isotype Ctrl (RTK4174)	BioLegend	400739;
TotalSeq-A0241 Armenian Hamster IgG Isotype Ctrl (HTK888)	BioLegend	400973;
TotalSeq-A0242 anti-human CD192 (K036C2)	BioLegend	357229; RRID: AB_2750501
TotalSeq-A0244 anti-human CD102 (CBR-IC2/2)	BioLegend	328509; RRID: AB_2750381
TotalSeq-A0245 anti-human CD106 (STA)	BioLegend	305813; RRID: AB_2800788
TotalSeq-A0247 anti-human CD267 (1A1)	BioLegend	311913; RRID: AB_2783181
TotalSeq-A0248 anti-human CD62E (HAE-1f)	BioLegend	336017; RRID: AB_2783218
TotalSeq-A0249 anti-mouse/human IRF4 (IRF4.3E4)	BioLegend	Barcode: GGATTTGTATCTCCC
TotalSeq-A0250 anti-mouse/human KLRG1 (MAFA) (2F1/KLRG1)	BioLegend	138431; RRID: AB_2800648
TotalSeq-A0351 anti-human CD135 (BV10A4H2)	BioLegend	313317; RRID: AB_2783188
TotalSeq-A0352 anti-human FCeR1a (AER-37)	BioLegend	334641; RRID: AB_2750503
TotalSeq-A0353 anti-human CD41 (HIP8)	BioLegend	303737; RRID: AB_2783162
TotalSeq-A0354 anti-mouse TCR (MR9-4)	BioLegend	139517; RRID: AB_2810408
TotalSeq-A0355 anti-human CD137 (4B4-1)	BioLegend	309835; RRID: AB_2783173
TotalSeq-A0356 anti-human CD254 (MIH24)	BioLegend	347509; RRID: AB_2750377
TotalSeq-A0357 anti-human CD43 (CD43-10G7)	BioLegend	343209; RRID: AB_2800915
TotalSeq-A0358 anti-human CD163 (GHI/61)	BioLegend	333635; RRID: AB_2750343
TotalSeq-A0359 anti-human CD83 (HB15e)	BioLegend	305339; RRID: AB_2800784
TotalSeq-A0360 anti-human CD357 (108-17)	BioLegend	371225; RRID: AB_2801016
TotalSeq-A0361 anti-human CD59 (p282 (H19))	BioLegend	304709; RRID: AB_2750371
TotalSeq-A0362 anti-human CD309 (7D4-6)	BioLegend	359919; RRID: AB_2810571
TotalSeq-A0364 anti-human CD13 (WM15)	BioLegend	301729; RRID: AB_2783151
TotalSeq-A0366 anti-human CD184 (12G5)	BioLegend	306531; RRID: AB_2800790
TotalSeq-A0367 anti-human CD2 (TS1/8)	BioLegend	Barcode: TACGATTTGTCAGGG
TotalSeq-A0368 anti-human CD226 (11A8)	BioLegend	338335; RRID: AB_2783220
TotalSeq-A0369 anti-human CD29 (TS2/16)	BioLegend	303027; RRID: AB_2783160
TotalSeq-A0370 anti-human CD303 (201A)	BioLegend	354239; RRID: AB_2800952
TotalSeq-A0371 anti-human CD49b (P1E6-C5)	BioLegend	359311; RRID: AB_2750363
TotalSeq-A0373 anti-human CD81 (5A6)	BioLegend	349521; RRID: AB_2800930
TotalSeq-A0374 anti-human CD98 (MEM-108)	BioLegend	315605; RRID: AB_2750369
TotalSeq-A0375 anti-human IgG (M1310G05)	BioLegend	410725; RRID: AB_2783329
TotalSeq-A0376 anti-mouse CD195 (CCR5) (HM-CCR5)	BioLegend	107019; RRID: AB_2783049
TotalSeq-A0377 anti-mouse CD197 (CCR7) (4B12)	BioLegend	Barcode: TTATTAACAGCCCAC
TotalSeq-A0378 anti-mouse CD223 (LAG-3) (C9B7W)	BioLegend	125229; RRID: AB_2783078
TotalSeq-A0379 anti-mouse CD62E (E-selectin) (RME-1/CD62E)	BioLegend	Barcode: CTCCCTTTGTAACAT
TotalSeq-A0381 anti-mouse Panendothelial Cell Antigen (MECA-32)	BioLegend	120507; RRID: AB_2783057
TotalSeq-A0382 anti-human CD177 (MEM-166)	BioLegend	315811: RRID: AB_2750554
TotalSeq-A0383 anti-human CD55 (JS11)	BioLegend	311317; RRID: AB_2750378
TotalSeq-A0384 anti-human IgD (IA6-2)	BioLegend	348243; RRID: AB_2783238
TotalSeq-A0385 anti-human CD18 (TS1/18)	BioLegend	302121; RRID: AB_2750382
TotalSeq-A0386 anti-human CD28 (CD28.2)	BioLegend	302955; RRID: AB_2783159
TotalSeq-A0387 anti-human TSLPR (1D3)	BioLegend	322907; RRID: AB_2800845
TotalSeq-A0388 anti-mouse CD152 (UC10-4B9)	BioLegend	106325; RRID: AB_2876417
TotalSeq-A0389 anti-human CD38 (HIT2)	BioLegend	303541; RRID: AB_2783161
TotalSeq-A0390 anti-human CD127 (A019D5)	BioLegend	351352; RRID: AB_2734366
TotalSeq-A0392 anti-human CD15 (W6D3)	BioLegend	323046; RRID: AB_2734304
TotalSeq-A0393 anti-human CD22 (S-HCL-1)	BioLegend	363514; RRID: AB_2734404
TotalSeq-A0394 anti-human CD71 (CY1G4)	BioLegend	334123; RRID: AB_2800884
TotalSeq-A0395 anti-human B7H4 (MIH43)	BioLegend	358114; RRID: AB_2734386
TotalSeq-A0396 anti-human CD26 (BA5b)	BioLegend	302720; RRID: AB_2734261
TotalSeq-A0397 anti-human CD193 (5e7)	BioLegend	310729; RRID: AB_2783174
TotalSeq-A0398 anti-human CD115 (9-4D2-1E4)	BioLegend	347325; RRID: AB_2783237
TotalSeq-A0399 anti-human CD204 (7C9C20)	BioLegend	371909; RRID: AB_2810584
TotalSeq-A0400 anti-human CD144 (BV9)	BioLegend	348517; RRID: AB_2783239
TotalSeq-A0401 anti-human CD301 (H037G3)	BioLegend	354707; RRID: AB_2810566
TotalSeq-A0402 anti-human CD1a (HI149)	BioLegend	300133; RRID: AB_2783146
TotalSeq-A0404 anti-human CD63 (H5C6)	BioLegend	353035; RRID: AB_2783249
TotalSeq-A0405 anti-human CD284 (HTA125)	BioLegend	312817; RRID: AB_2783183
TotalSeq-A0406 anti-human CD304 (12C2)	BioLegend	354525; RRID: AB_2783261
TotalSeq-A0407 anti-human CD36 (5-271)	BioLegend	336225; RRID: AB_2800892
TotalSeq-A0408 anti-human CD172a (15-414)	BioLegend	372109; RRID: AB_2783285
TotalSeq-A0409 anti-human CD85g (17G10.2	BioLegend	326411; RRID: AB_2750555
TotalSeq-A0411 Polyclonal Clec4F (Custom)	BioLegend	Barcode: TATGCTGTGGCTATG
TotalSeq-A0414 Polyclonal VSIG4 (Custom)	BioLegend	Barcode: ACGTATATCACGTTG
TotalSeq-A0415 anti-P2RY12 (S16007D)	BioLegend	848009; RRID: AB_2783419
TotalSeq-A0416 anti-mouse CD300LG (Nepmucin) (ZAQ5)	BioLegend	147105; RRID: AB_2783116
TotalSeq-A0417 anti-mouse CD163 (S16007D)	BioLegend	155303; RRID: AB_2814058
TotalSeq-A0418 anti-human CD243 (4E3.16)	BioLegend	919407: RRID: AB_2810817
TotalSeq-A0419 anti-human CD72 (3F3)	BioLegend	316205; RRID: AB_2783189
TotalSeq-A0420 anti-human CD158 (HP-MA4)	BioLegend	339515; RRID: AB_2800901
TotalSeq-A0421 anti-mouse CD49b (HMa2)	BioLegend	103523; RRID: AB_2819796
TotalSeq-A0422 anti-mouse CD172a (SIRPα) (P84)	BioLegend	144033; RRID: AB_2800670
TotalSeq-A0423 anti-human MerTK (5900H11G1E3)	BioLegend	367617; RRID: AB_2801011
TotalSeq-A0424 anti-mouse CD14 (Sa14-2)	BioLegend	123333; RRID: AB_2800591
TotalSeq-A0426 anti-mouse CD192 (CCR2) (SA203G11)	BioLegend	150625; RRID: AB_2783122
TotalSeq-A0427 anti-human FOLR2 (94b/FOLR2)	BioLegend	391707; RRID: AB_2783289
TotalSeq-A0428 anti-human TIM4 (9F4)	BioLegend	354009; RRID: AB_2783258
TotalSeq-A0429 anti-mouse CD48 (HM48.1)	BioLegend	103447; RRID: AB_2800558
TotalSeq-A0430 anti-human CD171 (L1-OV198.5)	BioLegend	371609; RRID: AB_2801019
TotalSeq-A0432 anti-human CD320 (3F4)	BioLegend	800319; RRID: AB_2801138
TotalSeq-A0433 anti-human CD325 (8C11)	BioLegend	350817; RRID: AB_2810555
TotalSeq-A0434 anti-mouse/human ReceptorD4	BioLegend	Barcode: TCTCTGCACCGCTTT
TotalSeq-A0435 anti-mouse/human GABRB3	BioLegend	Barcode: GGTGTGAGCAGTTCT
TotalSeq-A0437 anti-mouse/human CD207 (4C7)	BioLegend	Barcode: CGATTTGTATTCCCT
TotalSeq-A0438 anti-mouse/rat KKC2	BioLegend	Barcode: GAGCTTGTACCGCTT
TotalSeq-A0439 anti-mouse CD201 (RCR-16)	BioLegend	141509; RRID: AB_2800655
TotalSeq-A0440 anti-mouse CD169 (Siglec-1) (N1/12)	BioLegend	142425; RRID: AB_2783106
TotalSeq-A0441 anti-mouse CD71 (3D6.112)	BioLegend	113824; RRID: AB_2800574
TotalSeq-A0442 anti-mouse Notch (HMN1-12)	BioLegend	130617; RRID: AB_2783085
TotalSeq-A0443 anti-mouse CD41 (MWReg30)	BioLegend	133937; RRID: AB_2800635
TotalSeq-A0444 anti-mouse CXCR4 (L276F12)	BioLegend	146520; RRID: AB_2800682
TotalSeq-A0445 anti-human TMEM119 (A16075D)	BioLegend	853303; RRID: AB_2801201
TotalSeq-A0446 anti-human CD93 (VIMD2)	BioLegend	336121; RRID: AB_2750379
TotalSeq-A0448 anti-mouse CD204 (Msr1) (1F8C33)	BioLegend	154703; RRID: AB_2783126
TotalSeq-A0449 anti-mouse CD326 (Ep-CAM) (G8.8)	BioLegend	118237; RRID: AB_2800586
TotalSeq-A0450 anti-mouse IgM (RMM-1)	BioLegend	406535; RRID: AB_2783322
TotalSeq-A0551 anti-mouse CD301a (MGL1) (LOM-8.7)	BioLegend	145611; RRID: AB_2783114
TotalSeq-A0552 anti-mouse CD304 (Neuropilin-1) (3E12)	BioLegend	145215; RRID: AB_2750383
TotalSeq-A0554 anti-mouse CD309 (VEGFR2, Flk-1) (89B3A5)	BioLegend	121921; RRID: AB_2783066
TotalSeq-A0555 anti-mouse CD36 (HM36)	BioLegend	102621; RRID: AB_2800557
TotalSeq-A0556 anti-mouse CD370 (CLEC9A-DNGR1) (7H11)	BioLegend	Barcode: AACTCAGTTGTGCCG
TotalSeq-A0557 anti-mouse CD38 (90)	BioLegend	102733; RRID: AB_2750556
TotalSeq-A0558 anti-mouse CD55 (DAF) ( RIKO-3)	BioLegend	131809; RRID: AB_2783086
TotalSeq-A0559 anti-mouse CD63 (NVG-2)	BioLegend	143915; RRID: AB_2783109
TotalSeq-A0560 anti-mouse CD68 (FA-11)	BioLegend	137031; RRID: AB_2783099
TotalSeq-A0561 anti-mouse CD79b (Igβ) (HM79-12)	BioLegend	132811; RRID: AB_2783087
TotalSeq-A0562 anti-mouse CD83 (Michel-19)	BioLegend	121519; RRID: AB_2783061
TotalSeq-A0563 anti-mouse CX3CR1 (SA011F11)	BioLegend	149041; RRID: AB_2783121
TotalSeq-A0564 anti-mouse Folate Receptor β (FR-β) (10/FR2)	BioLegend	153307; RRID: AB_2800690
TotalSeq-A0565 anti-mouse MERTK (Mer) (2B10C42)	BioLegend	Barcode: AGTAGAGCAACTCGT
TotalSeq-A0566 anti-mouse CD301b (MGL2) (URA-1)	BioLegend	146817; RRID: AB_2783115
TotalSeq-A0567 anti-mouse Tim-4 (RMT4-54)	BioLegend	130011; RRID: AB_2783084
TotalSeq-A0568 anti-mouse/rat XCR1 (ZET)	BioLegend	148227; RRID: AB_2783120
TotalSeq-A0569 anti-human CD338 (5D3)	BioLegend	332021; RRID: AB_2783216
TotalSeq-A0570 anti-mouse/rat CD29 (HMβ1-1)	BioLegend	102233; RRID: AB_2783042
TotalSeq-A0571 anti-mouse IgD (11-26c.2a)	BioLegend	405745; RRID: AB_2783321
TotalSeq-A0572 anti-human C5L2 (1D(-M12)	BioLegend	342407; RRID: 2783226
TotalSeq-A0573 anti-mouse CD140a (APA5)	BioLegend	135917; RRID: AB_2783094
TotalSeq-A0574 anti-human CD235a (HI264)	BioLegend	349117; RRID: AB_2783242
TotalSeq-A0575 anti-human CD49a (TS2/7)	BioLegend	328315; RRID: AB_2783195
TotalSeq-A0576 anti-human CD49d (9F10)	BioLegend	304337; RRID: AB_2783166
TotalSeq-A0577 anti-human CD73 (AD2)	BioLegend	344029; RRID: AB_2783228
TotalSeq-A0578 anti-human CD79a	BioLegend	Barcode: CTTATCACCCGCTTT
TotalSeq-A0579 anti-human CD9 (H19a)	BioLegend	312119; RRID: AB_2783182
TotalSeq-A0580 anti-human mast cell tryptase	BioLegend	Barcode: ACTGATAGACCCGCT
TotalSeq-A0581 anti-human TCR (3C10)	BioLegend	351733; RRID: AB_2783246
TotalSeq-A0582 anti-human TCR (B6)	BioLegend	331433; RRID: AB_2800863
TotalSeq-A0583 anti-human TCR (B3)	BioLegend	331311; RRID: AB_2783207
TotalSeq-A0584 anti-human TCR (6B11)	BioLegend	342923; RRID: AB_2783227
TotalSeq-A0586 anti-human CD354 (Trem-26)	BioLegend	314910;
TotalSeq-A0588 anti-human CD202b (HIT2)	BioLegend	334213; RRID: AB_2810511
TotalSeq-A0590 anti-human CD305 (NKTA255)	BioLegend	342805; RRID: AB_2800911
TotalSeq-A0591 anti-human LOX1 (15C4)	BioLegend	358611; RRID: AB_2800987
TotalSeq-A0592 anti-human CD158b (DX27)	BioLegend	312615; RRID: AB_2800818
TotalSeq-A0593 anti-human CD203c (NP4D6)	BioLegend	324627; RRID: AB_2800849
TotalSeq-A0595 anti-mouse CD11a (M17/4)	BioLegend	101125; RRID: AB_2783036
TotalSeq-A0596 anti-mouse ESAM (1G8/ESAM)	BioLegend	136209; RRID: AB_2800642
TotalSeq-A0597 anti-human CD209 (9E9A8)	BioLegend	330119; RRID: AB_2783206
TotalSeq-A0599 anti-human CD158e1 (DX9)	BioLegend	312723; RRID: AB_2800819
TotalSeq-A0600 anti-human CD158f (UP-R1)	BioLegend	341307; RRID: AB_2800904
TotalSeq-A0801 anti-human CD337 (P30-15)	BioLegend	325221; RRID: AB_2800852
TotalSeq-A0803 anti-human CD253 (RIK-2)	BioLegend	308211; RRID: AB_2800803
TotalSeq-A0804 anti-human CD186 (K041E5)	BioLegend	356021; RRID: AB_2800961
TotalSeq-A0807 anti-mouse CD200R (OX-110)	BioLegend	123913; RRID: AB_2800594
TotalSeq-A0808 anti-mouse CD193 (J073E5)	BioLegend	144523; RRID: AB_2800673
TotalSeq-A0809 anti-mouse CD200R3 (Ba13)	BioLegend	142209; RRID: AB_2800657
TotalSeq-A0810 anti-mouse CD138 (281-2)	BioLegend	142532; RRID: AB_2800658
TotalSeq-A0811 anti-mouse CD317 (27)	BioLegend	127027; RRID: AB_2800623
TotalSeq-A0812 anti-mouse CD105 (MJ7/18)	BioLegend	120421; RRID: AB_2800587
TotalSeq-A0813 anti-mouse CD9 (MZ3)	BioLegend	124819; RRID: AB_2800600
TotalSeq-A0814 anti-human CD205 (HD30)	BioLegend	342211; RRID: AB_2800908
TotalSeq-A0816 anti-human CD271 (ME20.4)	BioLegend	345123; RRID: AB_2814273
TotalSeq-A0817 anti-human CD109 (W7C5)	BioLegend	323307; RRID: AB_2800848
TotalSeq-A0819 anti-human CD126 (UV4)	BioLegend	352813; RRID: AB_2800939
TotalSeq-A0821 anti-human CD164 (67D2)	BioLegend	324809; RRID: AB_2800850
TotalSeq-A0822 anti-human CD142 (NY2)	BioLegend	365207; RRID: AB_2801007
TotalSeq-A0824 anti-mouse P2X7R (1F11)	BioLegend	148711; RRID: AB_2800683
TotalSeq-A0825 anti-mouse CD371 (5D3/Clec12a)	BioLegend	143407; RRID: AB_2800668
TotalSeq-A0826 anti-human CD307c (H5/FcRL3)	BioLegend	374411; RRID: AB_2801022
TotalSeq-A0827 anti-mouse CD22 (OX-97)	BioLegend	126113; RRID: AB_2800614
TotalSeq-A0828 anti-human CD307d (413D12)	BioLegend	340209; RRID: AB_2800902
TotalSeq-A0829 anti-human CD307e (509f6)	BioLegend	340307; RRID: AB_2800903
TotalSeq-A0830 anti-human CD319 (162.1)	BioLegend	331821; RRID: AB_2800872
TotalSeq-A0831 anti-human CD138 (DL-101)	BioLegend	352325; RRID: AB_2800938
TotalSeq-A0834 anti-mouse CD39 (Duha59)	BioLegend	143813; RRID: AB_2800669
TotalSeq-A0835 anti-mouse CD314 (NKG2D) (CX5)	BioLegend	130215; RRID: AB_2814023
TotalSeq-A0836 anti-mouse DR3 (4C12)	BioLegend	144413; RRID: AB_2814048
TotalSeq-A0837 anti-mouse IL33RA (DIH9)	BioLegend	145317; RRID: AB_2800680
TotalSeq-A0839 anti-mouse Ly49H (3D10)	BioLegend	144715; RRID: AB_2814049
TotalSeq-A0841 anti-mouse Ly49D (4E5)	BioLegend	138309; RRID: AB_2800647
TotalSeq-A0843 anti-human CD199 (L053E8)	BioLegend	358919; RRID: AB_2810569
TotalSeq-A0844 anti-human CD45RB (MEM-55)	BioLegend	310209; RRID: AB_2810471
TotalSeq-A0845 anti-human CD99 (3B2/TA8)	BioLegend	371317: RRID: AB_2801017
TotalSeq-A0846 anti-mouse CD185 (L138D7)	BioLegend	145535; RRID: AB_2800681
TotalSeq-A0848 anti-mouse TIGIT (1G9)	BioLegend	142115; RRID: AB_2800656
TotalSeq-A0849 anti-mouse CD80 (16-10A1)	BioLegend	104745; RRID: AB_2813935
TotalSeq-A0850 anti-mouse CD49a (Hmα1)	BioLegend	142613; RRID: AB_2800659
TotalSeq-A0851 anti-mouse CD1d (1B1)	BioLegend	123529; RRID: AB_2800593
TotalSeq-A0852 anti-mouse CD226 (10E5)	BioLegend	128823; RRID: AB_2810393
TotalSeq-A0853 anti-human CD371 (50C1)	BioLegend	353613; RRID: AB_2800948
TotalSeq-A0857 anti-mouse CD34 (HM34)	BioLegend	128619; RRID: AB_2810392
TotalSeq-A0858 anti-human CD46 (TRA-2-10)	BioLegend	352415; RRID: AB_2810557
TotalSeq-A0861 anti-human CD151 (50-6)	BioLegend	350409; RRID: AB_2810554
TotalSeq-A0862 anti-human CD218a (H44)	BioLegend	313815; RRID: AB_2810476
TotalSeq-A0863 anti-human CD257 (1D6)	BioLegend	366509; RRID: AB_2810575
TotalSeq-A0866 anti-human CLEC1B (AYP1)	BioLegend	372009; RRID: AB_2814333
TotalSeq-A0867 anti-human CD94 (DX22)	BioLegend	305521; RRID: AB_2814142
TotalSeq-A0868 anti-human IgE (MHE-18)	BioLegend	325517; RRID: AB_2814186
TotalSeq-A0869 anti-human CD365 (1D12)	BioLegend	353907; RRID: AB_2814294
TotalSeq-A0870 anti-human CD150 (A12)	BioLegend	306313; RRID: AB_2814146
TotalSeq-A0871 anti-human CD162 (KPL-1)	BioLegend	328821; RRID: AB_2814192
TotalSeq-A0875 anti-mouse TLR4 (MTS510)	BioLegend	117614; RRID: AB_2810352
TotalSeq-A0876 anti-mouse CD300c_d (TX52)	BioLegend	148005; RRID: AB_2810413
TotalSeq-A0877 anti-mouse JAML (4E+10)	BioLegend	128507; RRID: AB_2810391
TotalSeq-A0881 anti-mouse CD272 (6A6)	BioLegend	139113; RRID: AB_2814041
TotalSeq-A0882 anti-mouse PIRA_PIRB (6C1)	BioLegend	144105; RRID: AB_2810412
TotalSeq-A0883 anti-mouse CD26 (H194-112)	BioLegend	137811; RRID: AB_2810405
TotalSeq-A0884 anti-mouse DLL1 (HMD1-3)	BioLegend	128315; RRID: AB_2810390
TotalSeq-A0885 anti-mouse CD270 (HMHC-1B18)	BioLegend	136307; RRID: AB_2810403
TotalSeq-A0890 anti-mouse 4_1BB (TKS-1)	BioLegend	107109: RRID: AB_2813955
TotalSeq-A0891 anti-mouse ENPP (YE1/19.1)	BioLegend	149209; RRID: AB_2814052
TotalSeq-A0892 anti-mouse CD2 (RM2-5)	BioLegend	100117; RRID: AB_2810312
TotalSeq-A0894 anti-human Ig (MHL-38)	BioLegend	316531; RRID: AB_2810479
TotalSeq-A0895 anti-mouse/human Mac2 (LM3/38)	BioLegend	125421; RRID: AB_281384
TotalSeq-A0896 anti-human CD85j (GHI/75)	BioLegend	333723; RRID: AB_2814225
TotalSeq-A0897 anti-human CD23 (EBVCS-5)	BioLegend	338523; RRID: AB_2814235
TotalSeq-A0898 anti-human Ig (MHK-49)	BioLegend	316627; RRID: AB_2814170
TotalSeq-A0899 anti-human HLA-A2 (BB7.2)	BioLegend	343331; RRID: AB_2810540
TotalSeq-A0900 anti-human CD198 (L263G8)	BioLegend	360607; RRID: AB_2810572
TotalSeq-A0901 anti-human GARP (7B11)	BioLegend	352515; RRID: AB_2814283
TotalSeq-A0904 anti-mouse CD31 (390)	BioLegend	102437; RRID: AB_2810335
TotalSeq-A0905 anti-mouse CD107a (1D4B)	BioLegend	121635; RRID: AB_2810369
TotalSeq-A0916 anti-mouse CD124 (I015F8)	BioLegend	144809; RRID: AB_2814050
TotalSeq-A0917 anti-mouse CD95 (SA367H8)	BioLegend	152614; RRID: AB_2810418
TotalSeq-A0923 anti-human NKp80 (5D12)	BioLegend	346709; RRID: AB_2814274
TotalSeq-A5106 anti-mouse Ly6D	BioLegend	Barcode: ATGTCCTACCTCAAA

**Chemicals, peptides, and recombinantproteins**		

Antigenfix	Diapath	P0014
β-mercaptoethanol	Sigma-Aldrich	M3148
Calcium chloride dihydrate	Merck	1023821000
Collagenase A	Sigma-Aldrich	11088793001
D-(-)-Fructose	Merck-Millipore	1040071000
D(+)-Saccharose	VWR International	PROL27483.294
DAPI	Invitrogen	D1306; RIDD: AB_2629482
DMEM	Invitrogen	41965-039
Dnase I	Sigma-Aldrich	04 536 282 001
Donkey Serum	Abcam	ab7475
EDTA	Westburg	51234
EGTA	Sigma-Aldrich	E3889
Eosin	VWR International	MERC 1.15935
FcBlock 2.4G2	Bioceros	N/A
FCS	Bodinco	5010
Fixable Viability due Live/Dead - eFluor506	eBioscience	65-0866-18
Fixable Viability due Live/Dead - eFluor780	eBioscience	65-0865-18
Fixation/Permeabilization Solution Kit	BD Cytofix/Cytoperm	554714
FoxP3 Transcription factor staining buffer kit	eBioscience	00-5523-00
Gentamicin	Gibco	15710-049
GlutaMAX	Thermo Fisher	35050-038
Gluteraldehyde 25%	Sigma-Aldrich	G5882
Goat Serum	Sigma-Aldrich	G9023
Hematoxylin	VWR International	MERC1.05174
HEPES	Sigma-Aldrich	H3375
Isopropanol	Vel	T0108
Methanol	Merck Millipore	13680502
Paraformaldehyde 10%	EMS	15712
Phenol Red	Sigma-Aldrich	P3532
ProLong Diamond	Thermo Fisher	P36970
Rat Serum	Sigma-Aldrich	R9759
Ribonucleoside Vanadyl Complexes	Sigma-Aldrich	R3380-5ML
Roche Protector RNAse inhibitor	Sigma-Aldrich	3335399001
RPMI 1640	Gibco	52400-025
Saponin	Sigma-Aldrich	4521
10X SDS	Sigma-Aldrich	71736-100ML
Sodium bicarbonate	Sigma-Aldrich	792519
Sodium chloride	Sigma-Aldrich	746398
Sodium Dihydrogen Phosphate Monohydrate	Sigma-Aldrich	1063461000
Sodium phosphate dibasic dihydrate	Sigma-Aldrich	71643
Tissue-Tek O.C.T	Sakura Finetek	4583
UltraPure™ Salmon Sperm DNA Solution	Thermo Fisher Scientific	15632011
Xylene	Prolabo	PROL28973.363

**Critical commercial assays**		

ALLin HS Red Taq Mastermix 2x	highQu	HQ.HSM0350
RNEasy Plus Micro Kit	QIAGEN	74034
SensiFAST cDNA Synthesis Kit	Bioline	BIO-65054
SensiFAST SYBR No-ROX Kit	Bioline	BIO-98020
Visium Spatial Gene Expression Slide and Reagent Kit	10X Genomics	1000184

**Oligonucleotides:qPCR**		

*Flt3* – qPCR FWD	IDT	GTGACTGGCCCCCTGGATAACGAG
*Flt3* – qPCR REV	IDT	TCCAAGGGCGGGTGTAACTGAACT
*Xcr1* - qPCR FWD	IDT	AGAGACACCGAACAGTCAGGCT
*Xcr1* - qPCR REV	IDT	TGTCCAGTTGCTGAAGGCTCTC
*Cd209a* - qPCR FWD	IDT	GCACTCCATCAAAGGCTTTGGC
*Cd209a* - qPCR REV	IDT	CAAACAGCTAGGAAGAGCACCTG
*Ccr7* - qPCR FWD	IDT	AGAGGCTCAAGACCATGACGGA
*Ccr7* - qPCR REV	IDT	TCCAGGACTTGGCTTCGCTGTA
*Mafb* - qPCR FWD	IDT	TGAATTTGCTGGCACTGCTG
*Mafb* - qPCR REV	IDT	AAGCACCATGCGGTTCATACA
*Cd5l* - qPCR FWD	IDT	GAGGACACATGGATGGAATGT
*Cd5l* - qPCR REV	IDT	ACCCTTGTGTAGCACCTCCA
*Cd207* - qPCR FWD	IDT	CCGAAGCGCACTTCACAGT
*Cd207* - qPCR REV	IDT	GCAGATACAGAGAGGTTTCCTCA
*Spp1* - qPCR FWD	IDT	CCATCTCAGAAGCAGAATCTCCTT
*Spp1* - qPCR REV	IDT	GGTCATGGCTTTCATTGGAATT
*Gata6* - qPCR FWD	IDT	CAGCAGGACCCTTCGAAAC
*Gata6* - qPCR REV	IDT	CCATTCATCTTGCTGTAGAGACC
*Gpnmb* - qPCR FWD	IDT	AGCACAACCAATTACGTGGC
*Gpnmb* - qPCR REV	IDT	CTTCCCAGGAGTCCTTCCA
*Pla2g7* - qPCR FWD	IDT	CTTCAAGCCCTTAGTGAGGACC
*Pla2g7* - qPCR REV	IDT	TGCGATGTCCTTTGGAGTCTGG
*Cd9* - qPCR FWD	IDT	GCTACTCGAGCCATGCCGGTCA AAGGAGGTAGC
*Cd9* - qPCR REV	IDT	CACTTGGTACCGACCATTTCTC GGCTCCTGCG
*Cd36* - qPCR FWD	IDT	GGAGCCATCTTTGAGCCTTCA
*Cd36* - qPCR REV	IDT	GAACCAAACTGAGGAATGGATCT
*Il1b* - qPCR FWD	IDT	GCAACTGTTCCTGAACTCAACT
*Il1b* - qPCR REV	IDT	ATCTTTTGGGGTCCGTCAACT
*Tnf* - qPCR FWD	IDT	TCTTCTCATTCCTGCTTGTGG
*Tnf* - qPCR REV	IDT	GGTCTGGGCCATAGAACTGA
*Il10* - qPCR FWD	IDT	AAGGCAGTGGAGCAGGTGAA
*Il10* - qPCR REV	IDT	CCAGCAGACTCAATACACAC
*Il18* - qPCR FWD	IDT	ACTGTACAACCGCAGTAATACGC
*Il18* - qPCR REV	IDT	AGTGAACATTACAGATTTATCCC
*Actb* - qPCR FWD	IDT	GCTTCTAGGCGGACTGTTACTGA
*Actb* - qPCR REV	IDT	GCCATGCCAATGTTGTCTCTTAT

**Oligonucleotides:Molecular cartography probes**		

*FLT3*	Resolve Bio.	P0C6W
*PDGFRB*	Resolve Bio.	P0D6V
*LGR5*	Resolve Bio.	P0E6T
*OTOA*	Resolve Bio.	P0N7M
*CLEC10A*	Resolve Bio.	P1C6X
*COLEC11*	Resolve Bio.	P1D6W
*GLS2*	Resolve Bio.	P1E6V
*KRT19*	Resolve Bio.	P1N6M
*SDS*	Resolve Bio.	P1N7N
*SIRPA*	Resolve Bio.	P2C6Y
*NGFR*	Resolve Bio.	P2D6X
*HAL*	Resolve Bio.	P2E6W
*GNLY*	Resolve Bio.	P2K1J
*SLC16A9*	Resolve Bio.	P2N7P
*CD1E*	Resolve Bio.	P3C6Z
*HGF*	Resolve Bio.	P3D6Y
*SLC38A1*	Resolve Bio.	P3N7Q
*ITGAX*	Resolve Bio.	P4422
*CD4*	Resolve Bio.	P4A2X
*ADAMTSL2*	Resolve Bio.	P4D6Z
*SLC40A1*	Resolve Bio.	P4N7R
*CDKN1C*	Resolve Bio.	P5A1X
*CPA3*	Resolve Bio.	P5C60
*MFAP4*	Resolve Bio.	P5D6L
*CXCR6*	Resolve Bio.	P6C61
*COL1A1*	Resolve Bio.	P6D60
*CD3E*	Resolve Bio.	P6J1Q
*TIMD4*	Resolve Bio.	P6N7T
*NCR1*	Resolve Bio.	P7A2L
*CXCL12*	Resolve Bio.	P7C62
*MARCO*	Resolve Bio.	P8C63
*MYH11*	Resolve Bio.	P8D62
*CD5L*	Resolve Bio.	P9C64
*ACTA2*	Resolve Bio.	P9D63
*IGFBP3*	Resolve Bio.	PAA11
*VSIG4*	Resolve Bio.	PAC65
*F8*	Resolve Bio.	PAD64
*NKG7*	Resolve Bio.	PC13E
*GPNMB*	Resolve Bio.	PCC66
*CMTM2*	Resolve Bio.	PDC67
*FLT4*	Resolve Bio.	PDD66
*CTSG*	Resolve Bio.	PDN7L
*CSF1R*	Resolve Bio.	PDR2R
*CD79A*	Resolve Bio.	PE13G
*EPCAM*	Resolve Bio.	PEW3P
*FCGR3B*	Resolve Bio.	PFC69
*FCGR3A*	Resolve Bio.	PFT1Q
*FOLR2*	Resolve Bio.	PFW6T
*LILRA4*	Resolve Bio.	PGC6A
*FCGBP*	Resolve Bio.	PGN61
*PLA2G7*	Resolve Bio.	PGN72
*DPT*	Resolve Bio.	PGX6T
*CLEC4C*	Resolve Bio.	PHC6C
*WNT2*	Resolve Bio.	PHD6A
*SPN*	Resolve Bio.	PHM1Z
*LHX6*	Resolve Bio.	PJD6C
*CD8A*	Resolve Bio.	PJJ11
*AXL*	Resolve Bio.	PJR2X
*VWF*	Resolve Bio.	PKD6D
*DCN*	Resolve Bio.	PKY4T
*FCN1*	Resolve Bio.	PMC6F
*RSPO3*	Resolve Bio.	PMD6E
*CCR7*	Resolve Bio.	PMH14
*HTRA3*	Resolve Bio.	PMJ68
*CCL21*	Resolve Bio.	PMM77
*CLEC12A*	Resolve Bio.	PNC6G
*GJA5*	Resolve Bio.	PND6F
*STAB2*	Resolve Bio.	PNJ69
*CD36*	Resolve Bio.	PNM78
*CD68*	Resolve Bio.	PPC6H
*LYVE1*	Resolve Bio.	PPJ6A
*IL7R*	Resolve Bio.	PPK14
*CD9*	Resolve Bio.	PPM79
*MSLN*	Resolve Bio.	PQJ6C
*ENHO*	Resolve Bio.	PQM7A
*TREM2*	Resolve Bio.	PR32Q
*SOX9*	Resolve Bio.	PRD6J
*RELN*	Resolve Bio.	PRM15
*F13A1*	Resolve Bio.	PRM7C
*SPP1*	Resolve Bio.	PSD6K
*FCER1A*	Resolve Bio.	PSM7D
*MAFB*	Resolve Bio.	PTA6P
*CD19*	Resolve Bio.	PTH1A
*FCGR1A*	Resolve Bio.	PTM7E
*C5AR1*	Resolve Bio.	PVA6Q
*GDF15*	Resolve Bio.	PVM7F
*CD1C*	Resolve Bio.	PWA6R
*FCGR2B*	Resolve Bio.	PWC2K
*WT1*	Resolve Bio.	PWD6P
*GLUL*	Resolve Bio.	PWG1E
*IGSF21*	Resolve Bio.	PWM7G
*XCR1*	Resolve Bio.	PXA6S
*CYP2E1*	Resolve Bio.	PXD6Q
*CD14*	Resolve Bio.	PXS37
*CLEC9A*	Resolve Bio.	PYA6T
*LILRB5*	Resolve Bio.	PYM7J
*THBD*	Resolve Bio.	PZA6V
*DES*	Resolve Bio.	PZC6T
*GHR*	Resolve Bio.	PZD6S
*NDST3*	Resolve Bio.	PZM7K
*Trem2*	Resolve Bio.	P0E2P
*Wt1*	Resolve Bio.	P0E4R
*Adgrg6*	Resolve Bio.	P0F4Q
*Lilra5*	Resolve Bio.	P0M7N
*Itgax*	Resolve Bio.	P1C1R
*Dcn*	Resolve Bio.	P1E4S
*Mmrn1*	Resolve Bio.	P1F4R
*Lpl*	Resolve Bio.	P1M7P
*Cd79a*	Resolve Bio.	P2662
*Cd36*	Resolve Bio.	P2A5Y
*Gpnmb*	Resolve Bio.	P2H7T
*Marco*	Resolve Bio.	P2M7Q
*Cd19*	Resolve Bio.	P3663
*Pdgfrb*	Resolve Bio.	P3E4V
*Adgre1*	Resolve Bio.	P3F4T
*Adamtsl2*	Resolve Bio.	P3K7S
*Mfap4*	Resolve Bio.	P3M7R
*Lyve1*	Resolve Bio.	P4961
*Axl*	Resolve Bio.	P4C1V
*Olfml3*	Resolve Bio.	P4E2T
*Atp6v0d2*	Resolve Bio.	P4K7T
*Mmp12*	Resolve Bio.	P4M7S
*C5ar1*	Resolve Bio.	P5K7V
*Msln*	Resolve Bio.	P5M7T
*Lhx6*	Resolve Bio.	P5T2F
*Fn1*	Resolve Bio.	P6952
*Ccr7*	Resolve Bio.	P6K7W
*Cd14*	Resolve Bio.	P7K7X
*Nrxn1*	Resolve Bio.	P7M7W
*Acta2*	Resolve Bio.	P8668
*Cd207*	Resolve Bio.	P8K7Y
*Cd5l*	Resolve Bio.	P9K7Z
*Rspo3*	Resolve Bio.	P9M7Y
*Ccr2*	Resolve Bio.	PAF40
*Slc40a1*	Resolve Bio.	PAM7Z
*Gls2*	Resolve Bio.	PAN7Y
*Sds*	Resolve Bio.	PCG40
*Clec10a*	Resolve Bio.	PCK70
*Spon2*	Resolve Bio.	PCM7L
*Pck1*	Resolve Bio.	PCN7Z
*Sirpa*	Resolve Bio.	PDH73
*Clec4f*	Resolve Bio.	PDK71
*Stab2*	Resolve Bio.	PDM70
*Krt19*	Resolve Bio.	PE07N
*Siglech*	Resolve Bio.	PEF43
*Clic5*	Resolve Bio.	PEK72
*Svep1*	Resolve Bio.	PEM71
*Cd9*	Resolve Bio.	PFH75
*Col1a1*	Resolve Bio.	PFK73
*Timd4*	Resolve Bio.	PFM72
*Pecam1*	Resolve Bio.	PFP5Z
*Cx3cr1*	Resolve Bio.	PG35J
*Ncam1*	Resolve Bio.	PGE46
*Colec11*	Resolve Bio.	PGK74
*Tmem119*	Resolve Bio.	PGM73
*Pdgfra*	Resolve Bio.	PHG45
*Cox6a2*	Resolve Bio.	PHK75
*Upk3b*	Resolve Bio.	PHM74
*Cxcl12*	Resolve Bio.	PJK76
*Wnt2*	Resolve Bio.	PJM75
*Reln*	Resolve Bio.	PK54J
*Fcgr1*	Resolve Bio.	PK86H
*Myh11*	Resolve Bio.	PK95F
*Des*	Resolve Bio.	PKK77
*Wnt9b*	Resolve Bio.	PKM76
*Sox9*	Resolve Bio.	PKT6L
*Chil3*	Resolve Bio.	PMH7A
*Dpt*	Resolve Bio.	PMK78
*Grip1*	Resolve Bio.	PND2A
*Ms4a7*	Resolve Bio.	PNH7C
*F13a1*	Resolve Bio.	PNK79
*Vsig4*	Resolve Bio.	PPH7D
*Flt3*	Resolve Bio.	PPK7A
*Spp1*	Resolve Bio.	PQD4F
*Flt4*	Resolve Bio.	PQK7C
*Epcam*	Resolve Bio.	PRD4G
*Plpp1*	Resolve Bio.	PRE4F
*Folr2*	Resolve Bio.	PRK7D
*Cd3e*	Resolve Bio.	PS56T
*Clec9a*	Resolve Bio.	PS66S
*Ghr*	Resolve Bio.	PSF4F
*Mafb*	Resolve Bio.	PSS66
*Gja5*	Resolve Bio.	PTK7F
*Cyp2e1*	Resolve Bio.	PVF4H
*Sept3*	Resolve Bio.	PW66W
*Vwf*	Resolve Bio.	PWE4K
*Lgr5*	Resolve Bio.	PWF4J
*Hal*	Resolve Bio.	PWK7H
*Prox1*	Resolve Bio.	PWS25
*Xcr1*	Resolve Bio.	PX86V
*Hgf*	Resolve Bio.	PXK7J
*Itgae*	Resolve Bio.	PY86W
*Spn*	Resolve Bio.	PYE7R
*Igfbp3*	Resolve Bio.	PYK7K
*Cd209a*	Resolve Bio.	PYM6H
*Glul*	Resolve Bio.	PZ81R
*Itgb7*	Resolve Bio.	PZK7M
*Mgl2*	Resolve Bio.	PZM6J

**Software and algorithms**		

Adobe Illustrator	Adobe	www.adobe.com
Cell Ranger	10X Genomics	https://support.10xgenomics.com/single-cell-gene-expression/software/pipelines/latest/what-is-cell-ranger
FastCAR R Package	N/A	https://github.com/LungCellAtlas/FastCAR
Seurat R Package V3	(Stuart et al., 2019)	https://satijalab.org/seurat/
Scater R Package	(McCarthy et al., 2017)	https://bioconductor.org/packages/release/bioc/html/scater.html
BioMart	N/A	https://www.ensembl.org/biomart/martview/3e2c65a5e3f783f8c9e5d648e4b64126
pheatmap R package	N/A	https://rdrr.io/cran/pheatmap/
ggplot2	(Wickham 2016)	https://ggplot2.tidyverse.org
Scanpy	(Wolf et al., 2018)	https://scanpy.readthedocs.io/en/stable/
PyTorch	N/A	https://pytorch.org
TotalVI	(Gayoso et al., 2021)	https://docs.scvi-tools.org/en/stable/user_guide/models/totalvi.html
ScVI	(Lopez et al., 2018)	https://docs.scvi-tools.org/en/stable/user_guide/models/totalvi.html
NicheNet	(Browaeys et al. 2019)	https://github.com/saeyslab/nichenetr
Enrichr	(Kuleshov et al., 2016)	http://amp.pharm.mssm.edu/Enrichr/
FlowJo v10.6.1	FlowJo	https://www.flowjo.com
GeneOntology	(Ashburner et al., 2000)	http://geneontology.org/
GraphPad Prism 9	GraphPad	https://www.graphpad.com/
Harmony	(Korsunsky et al., 2019)	https://www.github.com/immunogenomics/harmony
Ilastik	(Berg et al., 2019)	https://www.ilastik.org/
ImageJ	(Schneider et al., 2012)	https://imagej.nih.gov/ij
Qi-Tissue	Quantitative Imagining Systems	https://www.qi-tissue.com
genexyz Polylux	Resolve Biosciences	
Single-Cell Signature Explorer algorithm	(Pont et al., 2019)	https://sites.google.com/site/fredsoftwares/products/single-cell-signature-explorer
WEB-based GEne SeT AnaLysis Toolkit	(Liao et al., 2019)	http://www.webgestalt.org/
Venn Diagram Generator	PSB, VIB-Ghent University	https://bioinformatics.psb.ugent.be/webtools/Venn/
Zen Black	ZEISS Microscopy	www.zeiss.com

**Experimental models**		

Mouse: B6(C)-Ccr2 ^tm1.1Cln/J^	The Jackson Laboratory	JAX: 027619
Mouse: C57BL/6j SPF	Janvier Labs	N/A
Mouse: B6-Clec4f^hDTR/YFP-CIPHE^	(Scott et al., 2016)	N/A
Mouse: B6-Clec4f^Cre-CIPHE^	(Scott et al., 2018)	N/A
Mouse: Alk1-flox	(Park et al., 2008)	N/A

### Resource availability

#### Lead contact

Further information and requests for resources and reagents should be directed to and will be fulfilled by the lead contact, Martin Guilliams (martin.guilliams@irc.vib-ugent.be).

#### Materials availability

This study did not generate new unique reagents.

### Experimental model and subject details

#### *In vivo* animal studies

##### Mice

WT C57Bl/6J mice (Janvier) were used for this study. Male and Female mice were used for all experiments, with the exception of NAFLD experiments were only male mice were used. For NAFLD experiments, mice were put on the SD or WD at 5 weeks of age and sacrificed after either 24 or 36 weeks on the diet as indicated. *Fcgr1-Cre* mice (Scott et al., 2018) were obtained from Prof. Bernard Malissen, CIML, Marseille and *Clec4f-Cre* mice (Scott et al., 2018) were crossed with *Acvrl1*^fl/fl^ mice (Park et al., 2008) obtained from Paul Oh, Barrow Neurological Institute, Florida, USA. *Clec4f-Dtr* mice (Scott et al., 2016) were crossed to *CD45.1* mice (Janvier) for KC depletion and development experiments All mice were used between 6 and 12 weeks of age unless otherwise stated. All mice maintained at the VIB (Ghent University) under specific pathogen free conditions. All animals were randomly allocated to experimental groups. All experiments were performed in accordance with the ethical committee of the Faculty of Science, UGent and VIB.

##### Pig

Piglets (female, 10 weeks old) were purchased at a local farm and transported to the animal facilities of the Faculty of Veterinary Medicine. The animals were housed in isolation units as blood donors and had access to feed and water ad libitum. At 30 weeks of age, the animals were euthanized by intravenous injection of sodium pentobarbital 20% (60mg/2.5kg) and livers were collected. The animal study was reviewed and approved by the Ethical Committee of the Faculty of Veterinary Medicine (EC2018/55).

##### Chicken

Study animals were clinically healthy Leghorn hens of approximately 58 weeks old collected from a commercial farm. The hens were housed at the Faculty of Veterinary Medicine according to acceptable welfare standards and were observed at least twice daily for health problems. Feed and water was offered *ad libitum*. The chickens were euthanized through intravenous injection (in the wing) with sodium pentobarbital. The EC approval number of this trial was EC2019/015.

##### Macaque

Male Cynomolgus macaques (≥2 years) were sourced from China and supplied by Guangzhou Xiangguan Biotech Co., Ltd and confirmed healthy before being assigned to the study. Animal handling, husbandry and euthanasia was performed by WuxiAppTec Co., Ltd., China according to local ethical guidelines (AAALAC accredited 2010). Study animals consists of 4 groups, orally dosed once with a Janssen proprietary immune modulator or vehicle control. Vehicle control animals only were used in this study. Liver tissue samples were snap-frozen immediately after euthanasia. Samples were thawed once before shipping to Ghent for snRNA-seq analysis.

##### Hamster

Female syrian hamsters (Janvier) were housed per one or two in ventilated isolator cages at a temperature of 21°C, humidity of 55% and 12:12 dark/light cycles, with access to food and water *ad libitum* and cage enrichment. All hamsters had SPF status at arrival and manipulations were performed in a laminar flow cabinet. Housing conditions and experimental procedures were approved by the ethical committee of KU Leuven (license P015-2020). Animals were euthanized at 6-8 weeks of age by intraperitoneal injection of 200 mg/mL sodium pentobarbital and livers were collected for analysis.

##### Zebrafish

Zebrafish were maintained under standard conditions, according to FELASA guidelines (Alestrom et al., 2019). All experimental procedures were approved by the ethical committee for animal welfare (CEBEA) from the ULB (Université Libre de Bruxelles) (Protocol #594N). The following transgenic lines at 6 months of age were used: *Tg(mpeg1:EGFP)*^*gl22*^ (Ellett et al., 2011); Tg(*kdrl:Cre*)^*s89*^ (Bertrand et al., 2010); *Tg*(*actb2:loxP-STOP-loxP-DsRed*^*express*^)^*sd5*^ (Bertrand et al., 2010) enabling macs to be sorted for sequencing as DsRed, GFP double positive cells.

#### Patient studies

Patient studies were run in collaboration with Ghent University Hospital. Liver biopsies (1–2mm^3^) were isolated with informed consent from patients undergoing cholecystectomy or gastric bypass. In addition, liver biopsies were isolated from healthy adjacent tissue removed during liver resection due to colorectal cancer metastasis. In most cases, a second biopsy was also taken to evaluate liver histology. A full overview of all patient samples used in this study can be found in Table S6. Paraffin-embedded human liver samples were obtained through collaboration with Dr. Jan Lerut (Université Catholique de Louvain, UCL). All studies were performed in accordance with the ethical committee of the Ghent University Hospital (study numbers: 2015/1334 and 2017/0539).

### Method details

#### Isolation of liver cells

Liver cells were isolated by either *ex vivo* digestion (all species, except zebrafish) or *in vivo* liver perfusion (mice only) and digestion as described previously (Bonnardel et al., 2019; Scott et al., 2016). Briefly, for *ex vivo* digestion, livers were isolated, cut into small pieces and incubated with 1mg/ml Collagenase A and 10U/ml DNAse at 37^°^C for 20 mins with shaking. For *in vivo* digestion, after retrograde cannulation, livers were perfused for 1-2mins with an EGTA-containing solution, followed by a 5min (6ml/min) perfusion with 0.2mg/ml collagenase A. Livers were then removed, minced and incubated for 20mins with 0.4mg/ml collagenase A and 10U/ml DNase at 37°C. All subsequent procedures were performed at 4°C. Samples were filtered over a 100μm mesh filter and red blood cells were lysed. Samples were again filtered over a 40μm mesh filter. At this point *in vivo* digestion samples only were subjected to two centrifugation steps of 1 min at 50g to isolate hepatocytes. Remaining liver cells (leukocytes, LSECs and HSCs; *in vivo* protocol) and total cells from the *ex vivo* digests were centrifuged at 400g for 5mins before proceeding to antibody staining for flow cytometry. A combination of Collagenase A and DNase were used to digest livers in both protocols to minimize cleavage of surface epitopes.

Dissected livers from 6 months old transgenic zebrafish were triturated and treated with Liberase TM at 33°C for 20 min. Cells were then filtered through 40μm nylon mesh and washed with 2% FBS in PBS by centrifugation. Sytox Red was then added to the samples at a final concentration of 5nM to exclude nonviable cells before proceeding to flow cytometry. DsRed^+^GFP^+^ cells were then FACS-purified.

#### Isolation of liver nuclei

Nuclei were isolated from snap frozen liver tissue with a sucrose gradient as previously described (Habib et al., 2016). Briefly, frozen liver tissue is homogenized using Kimble Douncer grinder set in 1ml homogenization buffer with RNAse inhibitors. Homogenised tissue is then is then subjected to density gradient (29% cushion – Optiprep) ultracentrifugation (7700rpm, 4^°^C, 30 mins). After resuspension, nuclei are stained with DAPI and intact nuclei were FACS-purified from remaining debris.

#### Diet induced murine model of NAFLD/NASH

To induce NAFLD and NASH, mice were fed a western diet (WD) high in fat, sugar and cholesterol for 24 or 36 weeks as described previously (Remmerie et al., 2020). This consisted of 58% fat, 1% cholesterol (Research Diets; D09061703i) and drinking water was supplemented with 23.1g/L fructose (MPBio) and 18.9g/L sucrose (VWR). Control mice were fed a standard diet with 11kcal% fat with corn starch (D12328i; Research Diets).

#### Flow cytometry and cell sorting

Cells were pre-incubated with 2.4G2 antibody (Bioceros) to block Fc receptors and stained with appropriate antibodies at 4°C in the dark for 30-45 minutes. Cell viability was assessed using Fixable Viability dyes (eFluor780 or eFluor506; Thermo Fischer) and cell suspensions were analyzed with a BD FACSymphony or purified using a BD Symphony S6, BD FACSAria II or III. Nuclei were sorted on basis of DAPI positivity and size. Analysis was performed with FlowJo software (BD). Intracellular staining for CD207 was performed by fixing and permeabilizing extracellularly stained cells according to the manufacturer’s instructions using the FoxP3 Fixation/Permeabilization Kit (Thermo Fischer).

#### Confocal microscopy

Confocal staining was performed as described previously (Bonnardel et al., 2019). Immediately after sacrificing mice with CO_2_, inferior vena cava were cannulated and livers were perfused (4 mL/min) with Antigenfix (Diapath) for 5 min at room temperature. After excision, 2-3 mm slices of livers were fixed further by immersion in Antigenfix for 1h at 4°C, washed in PBS, infused overnight in 34% sucrose and frozen in Tissue-Tek OCT compound (Sakura Finetek). 20 μm-thick slices cut on a cryostat (Microm HM 560, Thermo Scientific) were rehydrated in PBS for 5 min, permeabilized with 0,5% saponin and non-specific binding sites were blocked for 30 min with 2% bovine serum albumin, 1% fetal calf serum and 1% donkey or goat serum for 30 minutes. Tissue sections were labeled overnight at 4°C with primary antibodies followed by incubation for 1h at room temperature with secondary antibodies. When two rat antibodies were used on the same section, the directly conjugated rat antibody was incubated for 1h after staining with the unconjugated and anti-rat secondary and after an additional blocking step with 1% rat serum for 30 minutes. Slides were mounted in ProLong Diamond, imaged with a Zeiss LSM780 confocal microscope (Carl Zeiss, Oberkochen, Germany) with spectral detector and using spectral unmixing and analyzed using ImageJ and QuPath software.

#### Confocal microscopy combined with RNAScope

Experiments were performed using the RNAScope Multiplex Fluorescent V2 Assay kit (ACDBio 323100). Probes targeting intronic regions for *Hs-Cd5l* (ACDBio 850511), Mfa-*Cd5l* (ACDBio 873211), Mm*-Cd5l* (ACDBio 573271), *Mm-Flt3* (ACDBio 487861), *Mm-Xcr1* (ACDBio 562371), *Mm-Mafb* (ACDBio 438531) and *Mm-Mgl2-O1* (ACDBio 822901) were custom-designed and synthesized. They were then labelled with TSA opal 520 (PerkinElmer FP1487001KT), TSA opal 540 (PerkinElmer FP1494001KT), TSA opal 570 (PerkinElmer FP1488001KT), TSA opal 620 (PerkinElmer FP1495001KT) or TSA opal 650 (PerkinElmer FP1496001KT). Tissues were fixed for 16 hours in AntigenFix (Diapath P0016), dehydrated and embedded in OCT as described above. Slices were pre-treated with hydrogen peroxide for 10 min and protease III for 20 min. The recommended Antigen retrieval step was not performed in order to preserve epitope integrity. Probes were hybridized and amplified according to the manufacturer’s instructions. Slides were then stained for protein markers as described above.

#### Visium

Mice were euthanized by means of carbon dioxide (CO_2_) overdose. The liver was excised and consequently trimmed, on ice, to smaller tissue pieces fitting the 10X Visium capture area. Trimmed tissue pieces were embedded in Tissue-Tek® O.C.T.™ Compound (Sakura) and snap frozen in isopentane (Sigma) chilled by liquid nitrogen. Embedded tissue pieces where stored at -80°C until cryosectioning.

A 10X Visium Spatial Gene expression slide was placed in the cryostat (Cryostar NX70 Thermo Fisher) 30 minutes prior to cutting. 10 μm sections where cut and placed within the capture area. Single 10X Visium Spatial Gene expression slides were stored in an airtight container at -80°C until further processing.

10X Visium cDNA libraries were generated according the manufacturer’s instructions. In short: Tissue sections where fixed in chilled Methanol. A H&E staining was performed to assess tissue morphology and quality. Tissue was lysed and reverse transcription was performed followed by second strand synthesis and cDNA denaturation. cDNA was transferred to a PCR tube and concentration was determined by qPCR. Spatially barcoded, full length cDNA was amplified by PCR. Indexed sequencing libraries where generated via End Repair, A-tailing, adaptor ligation and sample index PCR. Full length cDNA and indexed sequencing libraries were analyzed using the Qubit 4 fluorometer (Thermo Fisher) and Agilent 2100 BioAnalyzer.

#### Visium highly multiplexed protein

Liver slices were prepared as described above for the classical Visium protocol. Slices were dried for 1 min at 37°C and subsequently fixed using 1% paraformaldehyde in PBS. Next, slices were blocked for 30 min (2% BSA, 0.1ug/ul Salmon Sperm, 0.5% Saponin, 1 U/μl protector RNase inhibitor (Roche) in 3X SSC) and incubated with the oligo-conjugated antibody staining mix (2% BSA, 0.1μg/μl Salmon Sperm, 0.5% Saponin, 1 U/μl protector RNase inhibitor, 10uM polyT-blocking oligo (TTTTTTTTTTTTTTTTTTTTTTT^∗^T^∗^T^∗^/3InvdT/), in 3X SSC) for 1h at 4°C. Slides were mounted (90% glycerol, 1 U/μl protector RNase inhibitor) and imaged on Zeiss Axioscan Z1 at 20X magnification. Samples were then processed for a transcriptomic experiment as per manufacturer’s instructions (Visium, 10X Genomics) with modifications to also capture antibody tags. In short, tissue was permeabilized using Tissue Removal Enzyme (Tissue Optimization kit, 10x Genomics) for 9 minutes, as determined by a tissue optimization experiment (10X Genomics, Visium Spatial Tissue Optimization). After reverse transcription, 2 μl of 100 μM FB additive primer (CCTTGGCACCCGAGAATT^∗^C^∗^C^∗^A) per sample was added to the second strand synthesis mix. During cDNA amplification 1 μl of 0,2 μM FB additive primer (CCTTGGCACCCGAGAATT^∗^C^∗^C^∗^A) was added. After cDNA amplification, antibody products and mRNA derived cDNA were separated by 0.6X SPRI select. The purified full-length cDNA fraction was quantified by qRT-PCR using KAPA SYBR FAST-qPCR kit on a PCR amplification and detection instrument. After enzymatic fragmentation indexed sequencing libraries were generated via End Repair, A-Tailing, adaptor ligation and sample index PCR. The supernatant containing antibody product was cleaned up by two rounds of 1.9X SPRI select. Next, 45 μl of the purified antibody fraction was amplified with a 96 deep well reaction module: 95°C for 3 min; cycled 8 times: 95°C for 20 s, 60°C for 30 s, and 72°C for 20 s; 72°C for 5 min; end at 4°C. ADT libraries were purified once more with 1.6X SPRI select. Full length cDNA, indexed cDNA libraries and antibody libraries were analyzed using the Qubit 4 fluorometer (Thermo Fisher) and Agilent 2100 Bioanalyzer. The separation of the cDNA and ADT libraries were performed according to the manufacturer’s instructions (10X genomics).

#### MICS (MACSima^T^ Imaging Cyclic Staining) technology on the MACSima^T^ Imaging System by Miltenyi Biotec B.V. & Co. KG

The MACSima™ Imaging System is a fully automated instrument combining liquid handling with widefield microscopy for cyclic immunofluorescence imaging. In brief, staining cycles consisted of the following automated steps: immunofluorescent staining, sample washing, multi-field imaging, and signal erasure (photobleaching or REAlease).

Cryosectioned slices on slides were taken out of the -80°C storage and the appropriate MACSWell™ imaging frame was mounted immediately on the slide. An appropriate volume of ice-cold 4% PFA solution was added (according to the MACSWell™ imaging frames datasheet) and incubated for 10 minutes at room temperature. The slide was washed three times with MACSima Running Buffer. After washing the appropriate initial sample volume of MACSima Running Buffer was added (according to the MACSWell™ imaging frames datasheet). Right before the start of the MACSima ™ instrument a DAPI pre-staining was performed: the MACSima Running Buffer was removed from the sample to be analysed and stained for 10 min with a 1:10 dilution of a DAPI staining solution (volume depends on working volume for the different MACSwell™ formats, see datasheet). The DAPI staining solution was removed and 3 washing steps were performed (MACSima Running Buffer). Finally, the initial sample volume of MACSima Running Buffer was added. Details of the antibodies used can be found in the key resources table.

#### Molecular cartography™

##### Tissue sections

Liver was frozen and sectioned as described above for Visium analysis and liver slices were placed within capture areas on Resolve BioScience slides. Samples were then sent to Resolve BioSciences on dry ice for analysis. Upon arrival, tissue sections were thawed and fixed with 4% v/v Formaldehyde (Sigma-Aldrich F8775) in 1x PBS for 30 min at 4 °C. After fixation, sections were washed twice in 1x PBS for two min, followed by one min washes in 50% Ethanol and 70% Ethanol at room temperature. Fixed samples were used for Molecular Cartography™ (100-plex combinatorial single molecule fluorescence in-situ hybridization) according to the manufacturer’s instructions (protocol 3.0; available for download from Resolve’s website to registered users), starting with the aspiration of ethanol and the addition of buffer BST1 (step 6 and 7 of the tissue priming protocol). Briefly, tissues were primed followed by overnight hybridization of all probes specific for the target genes (see below for probe design details and target list). Samples were washed the next day to remove excess probes and fluorescently tagged in a two-step color development process. Regions of interest were imaged as described below and fluorescent signals removed during decolorization. Color development, imaging and decolorization were repeated for multiple cycles to build a unique combinatorial code for every target gene that was derived from raw images as described below.

##### Probe Design

The probes for 100 genes were designed using Resolve’s proprietary design algorithm. Briefly, the probe-design was performed at the gene-level. For every targeted gene all full-length protein-coding transcript sequences from the ENSEMBL database were used as design targets if the isoform had the GENCODE annotation tag ‘basic’ (Frankish et al., 2019; Yates et al., 2020). To speed up the process, the calculation of computationally expensive parts, especially the off-target searches, the selection of probe sequences was not performed randomly, but limited to sequences with high success rates. To filter highly repetitive regions, the abundance of *k*-mers was obtained from the background transcriptome using *Jellyfish* (Marçais and Kingsford, 2011). Every target sequence was scanned once for all *k*-mers, and those regions with rare *k*-mers were preferred as seeds for full probe design. A probe candidate was generated by extending a seed sequence until a certain target stability was reached. A set of simple rules was applied to discard sequences that were found experimentally to cause problems.

After these fast screens, every kept probe candidate was mapped to the background transcriptome using *ThermonucleotideBLAST* (Gans and Wolinsky, 2008) and probes with stable off-target hits were discarded. Specific probes were then scored based on the number of on-target matches (isoforms), which were weighted by their associated APPRIS level (Rodriguez et al., 2018), favoring principal isoforms over others. A bonus was added if the binding-site was inside the protein-coding region. From the pool of accepted probes, the final set was composed by greedily picking the highest scoring probes. Probe details are included in the key resources table.

##### Imaging

Samples were imaged on a Zeiss Celldiscoverer 7, using the 50x Plan Apochromat water immersion objective with an NA of 1.2 and the 0.5x magnification changer, resulting in a 25x final magnification. Standard CD7 LED excitation light source, filters, and dichroic mirrors were used together with customized emission filters optimized for detecting specific signals. Excitation time per image was 1000 ms for each channel (DAPI was 20 ms). A z-stack was taken at each region with a distance per z-slice according to the Nyquist-Shannon sampling theorem. The custom CD7 CMOS camera (Zeiss Axiocam Mono 712, 3.45 μm pixel size) was used.

For each region, a z-stack per fluorescent color (two colors) was imaged per imaging round. A total of 8 imaging rounds were done for each position, resulting in 16 z-stacks per region. The completely automated imaging process per round (including water immersion generation and precise relocation of regions to image in all three dimensions) was realized by a custom python script using the scripting API of the Zeiss ZEN software (Open application development).

##### Spot segmentation

The algorithms for spot segmentation were written in Java and are based on the ImageJ library functionalities. Only the iterative closest point algorithm is written in C++ based on the libpointmatcher library (https://github.com/ethz-asl/libpointmatcher).

##### Preprocessing

As a first step all images were corrected for background fluorescence. A target value for the allowed number of maxima was determined based upon the area of the slice in μm^2^ multiplied by the factor 0.5. This factor was empirically optimized. The brightest maxima per plane were determined, based upon an empirically optimized threshold. The number and location of the respective maxima was stored. This procedure was done for every image slice independently. Maxima that did not have a neighboring maximum in an adjacent slice (called z-group) were excluded. The resulting maxima list was further filtered in an iterative loop by adjusting the allowed thresholds for (Babs-Bback) and (Bperi-Bback) to reach a feature target value (Babs: absolute brightness, Bback: local background, Bperi: background of periphery within 1 pixel). This feature target values were based upon the volume of the 3D-image. Only maxima still in a z-group of at least 2 after filtering were passing the filter step. Each z-group was counted as one hit. The members of the z-groups with the highest absolute brightness were used as features and written to a file. They resemble a 3D-point cloud.

##### Final signal segmentation and decoding

To align the raw data images from different imaging rounds, images had to be corrected. To do so the extracted feature point clouds were used to find the transformation matrices. For this purpose, an iterative closest point cloud algorithm was used to minimize the error between two point-clouds. The point clouds of each round were aligned to the point cloud of round one (reference point cloud). The corresponding point clouds were stored for downstream processes. Based upon the transformation matrices the corresponding images were processed by a rigid transformation using trilinear interpolation.

The aligned images were used to create a profile for each pixel consisting of 16 values (16 images from two color channels in 8 imaging rounds). The pixel profiles were filtered for variance from zero normalized by total brightness of all pixels in the profile. Matched pixel profiles with the highest score were assigned as an ID to the pixel.

Pixels with neighbors having the same ID were grouped. The pixel groups were filtered by group size, number of direct adjacent pixels in group, number of dimensions with size of two pixels. The local 3D-maxima of the groups were determined as potential final transcript locations. Maxima were filtered by number of maxima in the raw data images where a maximum was expected. Remaining maxima were further evaluated by the fit to the corresponding code. The remaining maxima were written to the results file and considered to resemble transcripts of the corresponding gene. The ratio of signals matching to codes used in the experiment and signals matching to codes not used in the experiment were used as estimation for specificity (false positives).

##### Downstream analysis

Final image analysis was performed in ImageJ using genexyz Polylux tool plugin from Resolve BioSciences to examine specific Molecular Cartography™ signals.

#### RNA sequencing, CITE-seq, and qPCR

##### Sorting and RNA Isolation

40000-160000 cells of interest from livers of the different species were FACS-purified and pelleted by centrifugation at 400g for 5 mins. To ensure sufficient numbers of all cell types were present in our analyses, depending on the sample distinct populations of cells including Live CD45^+^, Live CD45^-^, Hepatocytes, Myeloid cells and Stromal cells were FACS-purified. When CITE-seq was to be performed, cells were then stained with 2.4G2 antibody to block Fc receptors and CITE-seq antibodies for 20mins at 4°C, before being washed in excess PBS with 2% FCS and 2mM EDTA. Antibody details are included in the key resources table. 40000-100000 nuclei were also FACS-purified based on DAPI expression. These were sorted into BSA coated tubes and pelleted by centrifuging for 3 mins at 400g and 5 mins at 600g sequentially. Cells/Nuclei were then resuspended in PBS with 0.04%BSA at ∼1000 cells/ml. Cell suspensions (target recovery of 8000-10000 cells) were loaded on a GemCode Single-Cell Instrument (10x Genomics, Pleasanton, CA, USA) to generate single-cell Gel Bead-in-Emulsions (GEMs). Single-cell RNA-Seq libraries were prepared using GemCode Single-Cell 3ʹGel Bead and Library Kit (10x Genomics, V2 and V3 technology) according to the manufacturer’s instructions. Briefly, GEM-RT was performed in a 96-Deep Well Reaction Module: 55°C for 45min, 85°C for 5 min; end at 4°C. After RT, GEMs were broken down and the cDNA was cleaned up with DynaBeads MyOne Silane Beads (Thermo Fisher Scientific, 37002D) and SPRIselect Reagent Kit (SPRI; Beckman Coulter; B23318). cDNA was amplified with 96-Deep Well Reaction Module: 98°C for 3 min; cycled 12 times : 98°C for 15s, 67°C for 20 s, and 72°C for 1 min; 72°C for 1 min; end at 4°C. Amplified cDNA product was cleaned up with SPRIselect Reagent Kit prior to enzymatic fragmentation. Indexed sequencing libraries were generated using the reagents in the GemCode Single-Cell 3ʹ Library Kit with the following intermediates: (1) end repair; (2) A-tailing; (3) adapter ligation; (4) post-ligation SPRIselect cleanup and (5) sample index PCR. Pre-fragmentation and post-sample index PCR samples were analyzed using the Agilent 2100 Bioanalyzer.

##### qPCR

RNA was extracted from 10000 sorted cells (gated using strategies shown) from livers of C57BL/6 mice using a RNeasy Plus micro kit (QIAGEN). Sensifast cDNA synthesis kit (Bioline) was used to transcribe total RNA to cDNA. Real-time RT-PCR using SensiFast SYBR No-Rox kit (Bioline) was performed to determine gene expression, therefore a PCR amplification and detection instrument LightCycler 480 (Roche) was used. Gene expression was normalized to β-actin gene expression. Primers used in the study can be found in the key resources table.

##### RNA sequencing analysis

sc/snRNA-seq libraries were loaded on an Illumina HiSeq or Illumina NovaSeq 6000 with sequencing settings recommended by 10X Genomics (26/8/0/98 – 2.1pM loading concentration, ADT and cDNA libraries were pooled in a 25:75 ratio). Visium sequencing libraries were loaded on an Illumina NovaSeq 6000 with sequencing settings recommended by 10X Genomics (28/10/10/75 – 2.1pM loading concentration). Sequencing was performed at the VIB Nucleomics Core (VIB, Leuven). The demultiplexing of the raw data was performed using CellRanger software (10x – version 3.1.0; cellranger mkfastq which wraps Illumina’s bcl2fastq). The reads obtained from the demultiplexing were used as the input for ‘cellranger count’ (CellRanger software), which aligns the reads to the mouse reference genome (mm10) using STAR and collapses to unique molecular identifier (UMI) counts. The result is a large digital expression matrix with cell barcodes as rows and gene identities as columns.

##### Preprocessing data

To remove ambient RNA, the FastCAR R package (v0.1.0) with a contamination chance cutoff of 0.05 was run on the samples separately before merging them. The UMI cut off was determined individually for the different samples, using the CellRanger web_summary output plot (see GitHub). The Scater R package (v1.14.6) was used for the preprocessing of the data. The workflow to identify the outliers, based on 3 metrics (library size, number of expressed genes and mitochondrial proportion) described by the Marioni lab (Lun et al., 2016) was followed. As a first step cells with a value x median absolute deviation (MADs) higher or lower than the median value for each metric were removed. This value was determined individually for the different datasets (see github). Secondly, the runPCA function (default parameters) of the Scater R package was used to generate a principal component analysis (PCA) plot. The outliers in this PCA plot were identified by the R package mvoutlier. By creating the Seurat object, genes that didn’t have an expression in at least 3 cells were removed. To normalize, scale and detecting the highly variable genes, the R package SCTransform (v0.2.1) was used. If batch correction (on sample level) was needed, the NormalizeData (log2 transformation), FindVariableFeatures and ScaleData functions of the Seurat R package (v3.1.2) were used in combination with the Harmony R package (v1.0). The Seurat pipeline was followed to find the clusters and create the UMAP plots. The number of principal components used for the clustering and the resolution were determined individually for the different datasets (see GitHub). On these initial UMAP plots we did multiple rounds of cleaning by removing proliferating and contaminating (e.g. doublets) cells. For non CITE-seq datasets the count data for the clean cells acquired by the previous steps were further processed with the scVI model (scvi Python package v0.6.7) (Lopez et al., 2018). Datasets including Cite-seq samples were further processed with the TotalVI model (Gayoso et al., 2021). The workflows described on scvi-tools.org were followed to generate new UMAPs, DEGs and DEPs. This information was further processed with the pheatmap R package (v1.0.12) to create heatmaps using the normalized values (denoised genes) calculated in the scVI/TotalVI workflow. The plots showing the expression of certain genes or proteins are created with the ggplot2 R package (v3.2.1) with a quantile cut off of 0.01.

For mouse all the ABs from the whitelist (181 ABs) were loaded into TotalVI, while for the other species only the added ABs were loaded into TotalVI. For the ‘human liver-pool of techniques and patients’ we noticed that the batch correction (between samples) faced difficulties for the hepatocytes and stellate cells as the cells all originated from snRNA-Seq samples, while the other cell types originated from both snRNA-seq and scRNA-seq samples. To overcome this issue we randomly allocated 30% of the hepatocytes to scRNA-seq samples which were not CITE-seq samples. We did the same for 30% of the stellate cells.

Heatmaps were made by scaling the normalized values (denoised values; calculated in the scVI/TotalVI workflow) using the scale_quantile function of the SCORPIUS R package (v1.0.7) and the pheatmap R package (v1.0.12). The plots showing the expression of certain genes or proteins were created based on the normalized values (denoised values) using a quantile cutoff of 0.99 and via either the ggplot2 R package (v3.2.1) or the scanpy.pl.umap function of the Scanpy Python package (v1.5.1).

##### Conserved human-mouse KC signature

To find the conserved human and mouse KC markers we started by identifying the human KC markers. We mapped the annotation of the human myeloid UMAP on the human pool of techniques/patients UMAP to identify the real KCs in this last UMAP. The real KCs were identified as the top part of the mac cluster. Using this new annotation we then calculated the DE genes and DE proteins for each cluster. Some genes are listed as marker for multiple clusters, only for the cluster where the gene had the highest score (raw_normalized_mean1/raw_normalized_mean2^∗^lfc_mean), the gene was kept as marker. This way we found 110 potential human KC markers. We then created a heatmap of these 110 genes (using denoised gene values scaled between 0 and 1) and filtered this heatmap by removing the genes where the scaled normalized value was higher than 0.50 in more than 30% of the cells of a certain cell type other than KCs. Except for the macs, we only removed a gene when it had a scaled normalized value higher than 0.50 in more than 70% of the macs. After this filtering we ended up with 36 human KC markers. Next we converted these human gene symbols into MGI IDs via the BioMart tool on the HGNC website (https://biomart.genenames.org/martform/#!/default/HGNC?datasets=hgnc_gene_mart). We found a MGI ID for 30 genes. We then converted these MGI IDs into mouse gene symbols via the MGI webtool (http://www.informatics.jax.org/batch/).

To identify the mouse KC markers we similarly mapped the annotation of the mouse myeloid UMAP on the mouse pool of techniques UMAP to identify the real KCs in this last UMAP. The real KCs matched with the mac cluster. Similarly as in human, the DE genes for each cluster was calculated and genes listed as marker for multiple clusters were dealt with in a similar way. This way we found 264 potential mouse KC markers. We then removed the genes that had a score (raw_normalized_mean1/raw_normalized_mean2^∗^lfc_mean) lower than 10 and ended up with 214 genes. We then created a heatmap of these 214 genes (using denoised gene values scaled between 0 and 1) and filtered this heatmap by removing the genes where the scaled normalized value was higher than 0.50 in more than 30% of the cells of a certain cell type other than KCs. After this filtering we ended up with 68 mouse KC markers. Next we converted these mouse gene symbols into MGI IDs via the MGI webtool (http://www.informatics.jax.org/batch/). We then converted these MGI IDs into human gene symbols via the BioMart tool on the HGNC website (https://biomart.genenames.org/martform/#!/default/HGNC?datasets=hgnc_gene_mart) and ended up with 60 genes.

At this point we found 30 human KC markers and 60 mouse KC markers. In a next step, we only kept the human KC markers that we identified as a Highly Variable Gene (HVG) in the mouse pool of techniques UMAP (20 genes) and the mouse KC markers that were identified as HVGs in the human pool of the techniques UMAP (30 genes). We next put these 20 mouse KC markers in SingleCellSignatureExplorer (Pont et al., 2019) to see where these genes are enriched in the mouse pool of techniques UMAP. In order to only get an enrichment in the KCs we decided to only use top 10 mouse KC markers (ordered on score), together with *Slc40a1* and *Hmox1*. We then started to add the top human KC markers as long as we keep the enrichment solely in the KCs. This way we ended up with final list of 15 human-mouse conserved KC markers.

We next converted these KC markers into the monkey, pig, chicken or zebrafish orthologs by looking up the human gene symbol on NCBI (https://www.ncbi.nlm.nih.gov/search/) and checking if there is an ortholog of the species of interest listed under the ‘Ortholog’ tab. The found orthologs were then used as input for the SingleCellSignatureExplorer tool.

##### Conversion of the CITE-seq data into a flow cytometry file

The protein normalized values (denoised values; calculated in the TotalVI workflow) were converted into an FCS file using the write.FCS function of the flowCore R package (v1.50.0).

#### Preprocessing Visium data

We first removed per sample all spots that were clear outliers compared to the location of the tissue. Each sample was then normalized individually using the SCTransform function of the Seurat R package (v3.2.3) with default parameters. All samples were then merged with the merge function of the Seurat R package (v3.2.3) with default parameters. Next, we determined the HVGs, created a PCA plot, performed clustering and created an UMAP plot as described in the spatial workflow available on the Seurat website (https://satijalab.org/seurat/articles/spatial_vignette.html). Clusters which showed high mitochondrial gene expression were removed. Spots located at the darker parts of the tissue were also removed as these parts are considered to be dead tissue or of bad quality.

#### Modelling of Visium data

##### Probabilistic graphical modeling

For modelling the cell type composition and zonation, spatial CITE-seq and transcriptomics data were analyzed using probabilistic graphical models, similar to what is used in tools such as cell2location and scVI. In brief, transcriptomics data was modelled as a NegativeBinomial distribution, parameterized with a mean μ and dispersion θ, the latter optimized as a free parameter for each gene. Visium Highly Multiplexed Protein data was modelled as a mixture of NegativeBinomials, with a $\mu_{background}$ and $\mu_{foreground}$ and a shared dispersion θ. The actual foreground/background signal within a modality was modelled as a ρ that depends on the latent space, and which is multiplied with the empirical library size to get μ. For Visium Highly Multiplexed Protein, $\rho_{background}$ was modelled as a latent variable specific for each gene. $\rho_{foreground}$ for Visium Highly Multiplexed Protein and ρ for RNA-seq were modelled as deterministic functions depending on the use case as described in the following paragraphs. The posterior of the probabilistic graphical model was inferred using black-box variational inference (Ranganath et al., 2013), in which the variational distribution was specified as a diagonal Normal distribution, transformed into the correct domain using transforms ($R\;\to R^{+}:e^{x}$, $R\;\to \Delta :\frac{e^{x_{i}}}{\sum_{i}e^{x_{i}}}$, $R\;\to \left[0,1\right]:\frac{e^{x}}{e^{x}+1}$). Free parameters within this model were optimized using gradient descent, with the ELBO as loss function and Adam as optimizer as implemented in Pytorch (Paszke et al., 2019) (pytorch.org). We used a learning rate of 0.01 for variational parameters, and 0.001 for parameters of the amortization functions.

##### Reference for deconvolution

To calculate the average expression of each gene within a cell type, we used a linear model in which both ρ and θ were modelled as a latent variable specific for each gene and cell type. The ρ for nuclei were multiplied with a gene-specific correction factor (optimized as a latent variable) that corrected for differences between scRNA-seq and snRNA-seq. Given that spatial transcriptomics data sequences the whole cell, the uncorrected ρ values were used for spatial deconvolution.

##### Deconvolution

To infer the proportions of each cell type within a spot, we used a model in which the gene expression is modelled as a linear combination of cell type proportions and average expression in each cell type:

$\rho_{spot,gene}=\nu_{spot,celltype}\times \rho_{celltype,gene}$

For $\rho_{celltype,gene}$ we adapted the values from the reference, but included

- A capture bias per gene, which corrects for technical and biological differences between spatial and sc/sn-RNA-seq. The capture bias was modelled as a latent variable with prior Normal(0,1)
- A red blood cell cell type, which was not included in the reference dataset but nonetheless had a dominant presence in the spatial data. The ρ of this cell type was set to zero for all genes except *Hbb-bt*, *Hbb-bs*, *Hba-a1*, *Hba-a2* for mouse and *HBB*, *HBA1*, *HBA2* for human, which were modelled as free parameters.
- Similarly, the expression of complement factors (*C3, C2*, *C4B/C4b*) within hepatocytes was modelled as free parameters.

A background signal shared for all spots was also modelled as follows:

$\mu_{spot,gene}=\rho_{spot,gene}\times lib\times foreground+\mu_{background}$

With foreground∈[0,1] a latent variable specific to each spot and $\mu_{background}\in R$ a latent variable specific to each gene.

A likelihood ratio test was used to assess whether a cell type was significantly present in a spot. Specifically, if x is the gene expression of all genes at a particular spot, we used Monte Carlo samples from the posterior to estimate:

$$\frac{P\left(x|\nu_{celltype}\right)}{P\left(x|\nu_{celltype}=\;0\right)}$$

A cell type was deemed significantly present if the log-likelihood was higher than 10.

##### Zonation

The zonation of spots was modelled as a univariate latent variable z∼Uniform(0,1) specific to each spot. This latent variable influenced the gene expression ρ using a spline function by using a gaussian basis function (σ=0.05) with 10 knots at uniform fixed positions. The coefficients of this spline were modelled as a latent variable specific for each gene, with prior a Gaussian random walk distribution, and the step $∼\;Normal\left(0,\;\sigma_{gene}\right)$. $\sigma_{gene}$ was determined empirically as 2 times the standard deviation of the log1p transformed expression values in the whole dataset. The variational parameters of the zonation $\mu_{z}$ and $\sigma_{z}$ were not optimized directly but were estimated using an amortization function. This amortization function used the count matrix as input, and estimated the variational parameters using the following layers: Linear (with 100 output dimensions), BatchNorm, ReLU, Linear (again with 100 output dimensions), ReLU, and a final Linear layer. This amortization function was used to transfer the zonation onto a different dataset, i.e., 1) to transfer the zonation trained on mouse spatial transcriptomics onto mouse Visium highly multiplexed protein and 2) to transfer the zonation trained on human low steatosis (<10%) onto human high steatosis (>30%).

#### Differential abundance along zonation

To determine the differential abundance of a cell type across zonation, the significant presence of a cell type within a spot ∈{0,1} was modelled using a spline function with the zonation of a cell type as input. The coefficients of this spline function were modelled as a latent variable with the step size ∼Normal(0,1). To determine differences in abundance between patients with high and low steatosis, we first modelled the zonation on human data on patients with steatosis < 10%. Potential interaction effects between zonation and steatosis status were then modelled using a spline function as before, but with a separate set of coefficients for both high and low steatosis. A likelihood ratio test was then used to determine whether this interaction was present significantly, by comparing the likelihood of this model with a model with shared coefficients.

#### Differential NicheNet

To analyze cell-cell communication in the hepatic mac niches, we applied Differential NicheNet, which is an extension of the default NicheNet pipeline to compare cell-cell interactions between different niches and better predict niche-specific ligand-receptor (L-R) pairs. It uses a flexible prioritization scheme that allows ranking L-R pairs according to several properties, such as niche- and region-specific expression of the L-R pair, ligand activity, and level of database curation. This in contrast to the default NicheNet pipeline which prioritizes expressed L-R pairs solely based on ligand activity predictions. All analyses were conducted according to the Differential NicheNet tutorial (https://github.com/saeyslab/nichenetr/blob/master/vignettes/differential_nichenet.md). As input to the Differential NicheNet pipeline, we used the data after normalization via SCTransform and integration of scRNA-seq and snRNA-seq according to the Seurat procedure for integration (Stuart et al., 2019).

For the mouse analyses, Differential NicheNet was first performed for the following 3 niche comparisons: 1) KCs versus central vein macs; 2) KCs versus capsule macs; 3) KCs versus LAMs. Following sender cell types were considered for these niches: KC niche: periportal hepatocytes, periportal LSECs, and periportal stellate cells; Central vein mac niche: central vein ECs and central vein fibroblasts; Capsule mac niche: mesothelial cells and capsule fibroblasts; LAM niche: cholangiocytes and bile duct fibroblasts.

Because of the preferentially periportal localization of KCs in the mouse liver, we also included a ‘region specificity' factor in the Differential NicheNet prioritization framework. This was done to increase the ranking of ligands that are more strongly expressed in periportal than pericentral niche cells. Periportal sender cells were determined after subclustering based on the following markers: *Hal* and *Sds* for hepatocytes; *Mecom*, *Msr1*, and *Efnb2* for LSECs; *Ngfr*, *Igfbp3*, and *Dach1* for stellate cells.

In the heatmap (Figure S8G), we show the prioritization scores of the top 40 ligands (and their highest scoring receptor) in the KC niche (score averaged over the 3 analyses), and of all the non-KC niche L-R pairs with a prioritization score ≥ the score of the lowest scoring KC L-R pair of this top 40. For each L-R pair/niche combination, we only displayed the score of the sender cell with the highest score (e.g. for the *Csf1*-*Csf1r* interaction in the KC niche, the score is shown for the LSEC-KC interaction because that score was higher than for Stellate–KC and Hepatocyte–KC; in the LAM niche, the score of *Csf1*-*Csf1r* is shown for the bile duct fibroblast – LAM interaction and not for the cholangiocyte–LAM interaction, etc.).

Because of the strong concordance between the top-ranked L-R pairs in these 3 non-KC mac niches, it was decided to also conduct a subsequent analysis in which the KC niche is compared against all non-KC hepatic mac niches combined. For this final ‘KC versus all non-KC mac analysis’, KCs were compared to central vein macs, capsule macs, and LAMs together, with the same sender cell types as described here above (but now analyzed together).

For the human analyses, Differential NicheNet was performed to compare the KC niche with the non-KC mac niches (similarly as the final analysis in mouse). For the KC niche, all hepatocytes, LSECs, and stellate cells were selected as sender cells; and KCs as receiver cells. For the non-KC mac niche, cholangiocytes, fibroblasts, and central vein ECs were considered as the sender cells; Mat. LAMs, Imm. LAMs, and Mac1s as the receiver cells (Figure 4H).

To find KC-niche-specific L-R pairs that are conserved across mouse and human, the individual mouse and human prioritization scores were averaged to form a ‘conservation score’. The 40 ligands (and maximally 3 of their highest scoring receptors) with the highest conservation score were selected for further analysis (note: the L-R pair should be expressed by the same sender-receiver pair in both species). In the circos plot (Figure 6C; Gu et al., 2014), only a subset of these top L-R pairs is shown to keep the figure clearly interpretable. Following ligands were not shown: *ITGA9*, *SEMA6D*, *JAM3*, *ITGB1* (stellate cells); *ITGA9*, *F8*, *CD274*, *HSP90B1* (LSECs); *C5*, *F9*, *F2*, *FGA*, *TF*, *TTR*, *COL18A1*, *COL5A3*, *SERPINA1*, *SERPINC1* (hepatocytes). The depicted target genes are KC-specific in both mouse and human, and a top-predicted target according to the NicheNet ligand-target regulatory potential scores. *NR1H3* was manually added as a *NOTCH2* target based on recent studies (Bonnardel et al., 2019).

#### Staining of human liver paraffin sections

Resected human liver was fixed in 4% formalin for 24-48h and subsequently embedded in paraffin. Samples were stored for 10-15 years at RT before analysis. Sections of 6 μm thick were cut using a Microm HM360 and mounted on a polarized glass slide. These sections were deparaffinized in xylene and rehydrated in a graded ethanol series. Antigen retrieval was performed by immersing the samples for 5 min in pH 8.3 TRIS-EDTA at 98°C. Slides were then cooled to RT and washed in PBS. Confocal staining was performed as described above.

#### Isolation and culture of BM monocytes with acetylated LDL

BM was isolated from the tibia and femur of mice by centrifugation. Red blood cells were lysed and single cell suspensions were stained with antibodies for flow cytometry. BM monocytes were sorted as live CD45+ CD11b+ Ly6G- Ly6C+ CD115+ cells using a BD FACSAria III. Monocytes were resuspended in DMEM/F12 media supplemented with 10% FCS, 30ng/ml CSF1, 2mM Glutamine and 100U/ml penicillin and streptomycin. 150,000 monocytes were seeded in each well of an adherent 24-well plate pre-coated with bovine collagen type I and cultured overnight (37C, 5% CO2). The following day 0, 25 or 50ng/ml of ac-LDL was added. Ac-LDL was kindly provided by Sophie Janssens, Ghent, Belgium who received the material from Wilfried Le Goff, Paris, France. 14 hours later cells were harvested and live F4/80+ cells were FACS-purified in RLT plus buffer containing 1% β-mercaptoethanol. RNA isolation, cDNA synthesis and qPCR were performed as described above.

#### Generation of bone marrow chimeras

Bone marrow chimeras were generated as described previously (Scott et al., 2016). Briefly, 6-12 week old *Clec4f-Dtr* mice (CD45.1) were anaesthetized by intraperitoneal administration of Ketamine (150 mg/kg) and Xylazine (10 mg/kg). Mice were lethally irradiated with 8 Gy, with the livers being protected with a 3-cm-thick lead cover. Once recovered from the anesthesia, mice were reconstituted by intravenous administration of 5-10×10^6^ BM cells from CD45.2 *Acvrl1*^fl/fl^ or *Fcgr1-CrexAcvrl1*^*fl/f*^*l* mice. 4 weeks after reconstitution mice were administrated a single dose of 500ng DT via intraperitoneal injection to deplete KCs. Chimerism was assessed 7 or 13 days later by flow cytometry and compared with chimerism levels in blood Ly6C^hi^ monocytes.

#### Administration of Fc traps

Clec4f-Dtr mice were administered 10mg/kg ALK1Fc, TGFβRIIFc or appropriate isotype controls (hIgG1 and mIgG2a; Acceleron Pharma) by intraperitoneal injection on days -1, 2, 3 and 5. On Day 0 mice were also administered a single dose of 500ng DT i.p. to deplete KCs. Livers were harvested at day 7 to assess KC development.

### Quantification and statistical analysis

In all experiments, data are presented as mean ±SEM and/or individual data points are presented unless stated otherwise. Statistical tests were selected based on appropriate assumptions with respect to data distribution and variance characteristics. Details of the precise test used for each analysis can be found in the figure legends. Statistical significance was defined as p<0.05. Sample sizes were chosen according to standard guidelines. Number of animals/patients is indicated as ‘‘n’’. The investigators were not blinded to the group allocation, unless otherwise stated.

### Additional resources

The sc/snRNA-sequencing, CITE-seq FCS files and spatial transcriptomics datasets will be made available for visualization, analysis and download at www.livercellatlas.org.

## Data Availability

The datasets generated during this study have been deposited in the Gene Expression Omnibus public database under accession number GSE192742.

## Supplemental information

Table S1. Differentially expressed genes (DEGs) and proteins (DEPs) per cell type in total mouse and human liver cell atlases, related to Figures 1, 4, S2, and S6

Table S2. Differentially expressed genes (DEGs) and proteins (DEPs) per cell type in mouse and human myeloid cell atlases, related to Figures 2, 4, 5, S3, S6, and S8

Table S3. Differentially expressed genes (DEGs) and proteins (DEPs) per cell type in mouse and human CD45− cell atlases, related to Figures 3, 6, and S4

Table S4. Differentially expressed genes (DEGs) per cell type in mouse stromal cell atlas, related to Figures 3 and S4

Table S5. Differentially expressed genes (DEGs) and proteins (DEPs) per cell type in mouse lymphoid cell atlas, related to Figures 1, 4, and S5

Table S6. Clinical data from patient liver biopsies used in the study, related to Figure 4

Table S7. Differentially expressed genes (DEGs) and proteins (DEPs) per cell type in mouse NAFLD lymphoid cell atlas, related to Figures 5 and S5

Table S8. Differentially expressed genes (DEGs) and proteins (DEPs) per cell type in mouse NAFLD myeloid cell atlas, related to Figures 5 and S6

Table S9. Differentially expressed genes (DEGs) and proteins (DEPs) per cell type in liver cell atlases from macaque, pig, hamster, chicken, and zebrafish, related to Figures 4 and S7

Table S10. Differentially expressed genes (DEGs) per cell type in mouse NAFLD stromal cell atlas, related to Figure 6
